# The Relationship Between Time Spent on Social Media and Adolescent Cigarette, E-cigarette, and Dual Use: A Longitudinal Analysis of the UK Millennium Cohort Study

**DOI:** 10.1093/ntr/ntae057

**Published:** 2024-04-04

**Authors:** Amrit Kaur Purba, Marion Henderson, Andrew Baxter, Anna Pearce, S Vittal Katikireddi

**Affiliations:** MRC/CSO Social and Public Health Sciences Unit, University of Glasgow, Glasgow, UK; MRC/CSO Social and Public Health Sciences Unit, University of Glasgow, Glasgow, UK; School of Social Work and Social Policy, University of Strathclyde, Glasgow, UK; MRC/CSO Social and Public Health Sciences Unit, University of Glasgow, Glasgow, UK; MRC/CSO Social and Public Health Sciences Unit, University of Glasgow, Glasgow, UK; MRC/CSO Social and Public Health Sciences Unit, University of Glasgow, Glasgow, UK

## Abstract

**Introduction:**

To estimate the effect of social media use in 14 year olds on risk of and inequalities in cigarette, e-cigarette, and dual use at 17 years, using the UK-representative Millennium Cohort Study (born 2000–2002).

**Aims and Methods:**

The relationship of time spent on social media (using questionnaires [*n* = 8987] and time-use-diaries [*n* = 2520]) with cigarette, e-cigarette, and dual use was estimated using adjusted odds ratios (AORs) or relative risk ratios (ARRRs). Effect modification was examined (using parental education as an indicator for socioeconomic circumstances) by comparing adjusted risk differences within low and high-parental education groups. Analyses accounted for prespecified confounders (identified via directed acyclic graphs), baseline outcome measures (to address reverse causality), sample design, attrition, and item-missingness (through multiple imputation).

**Results:**

Time spent on social media was associated with increased risk of cigarette, e-cigarette, and dual use in a dose–response manner. Social media use for ≥2 hours/day (vs. 1–<30 minutes) was associated with increased cigarette (AOR 2.76 [95% confidence interval 2.19 to 3.48]), e-cigarette (3.24 [2.59 to 4.05]), and dual use (ARRR 4.11 [2.77 to 6.08]). The risk of cigarette use among 30 minutes–<1 hour/day users (vs. non-users) were smaller in those with high versus low parental education (ARDs 1.4% vs. 12.4%). Similar findings were observed across the higher time categories. Analyses using time-use-diaries, in complete case samples, and with additional adjustment for baseline outcome measures generally revealed similar findings.

**Conclusions:**

After accounting for observed confounders and potential reverse causality, findings suggest social media use increases the risk of cigarette, e-cigarette, and dual use in a dose–response manner. Guidance addressing adolescent online safety should be prioritized.

**Implications:**

This study’s identification of a dose–response relationship and differential effects across socioeconomic groups, could assist in the development of guidance on time spent on social media. The adverse effects of social media use on adolescent cigarette, e-cigarette, and dual use supports legislation aimed at promoting adolescent online safety. Study findings strengthen calls to prohibit social media marketing of nicotine-related products and importantly highlight the need to increase awareness and understanding of the underlying algorithms which drive adolescent exposure to nicotine-related content on social media to ensure they are functioning in a way that best serves the adolescent population.

## Introduction

Tobacco use generally commences in adolescence.^1^ The higher prevalence of tobacco use in adolescent populations with greater deprivation is a key driver of health inequalities.^2,3^ E-cigarettes have provided a potential harm-reduction alternative to adult tobacco smoking.^4^ However, increased promotion and use of e-cigarettes among adolescents, combined with the recognized impact of nicotine on the developing adolescent brain, has prompted concern that e-cigarettes are creating a new generation of nicotine-dependent individuals, and may offer a gateway to future tobacco smoking.^4,5^

Use of new media, specifically social media has become almost ubiquitous among adolescents.^6^ Through its ability to encourage personal expression, improve information access, and strengthen connections, it can present several benefits to adolescent health and development.^6^ In contrast, time spent on social media, may influence adolescents’ exposure to user-generated nicotine-related content (eg, peer posts showcasing nicotine use), which may shape adolescents’ attitudes toward cigarette and e-cigarette use and result in increased uptake.^7–11^ Moreover, increased (and often unregulated) social media marketing by e-cigarette or tobacco corporations targeting adolescents may prompt uptake (eg, via cartoon-based strategies to promote use). These potential influences underpin the need to investigate social media’s potential role as a risk factor for adolescent nicotine use.^12,13^

Yet, the majority of evidence examines U.S. populations and causality remains unclear,^14^ with the potential for reverse causation (where those who use cigarettes or e-cigarettes may be more inclined to use social media) remaining largely unaddressed.^8,9^ Furthermore, in the absence of real-time objective social media data obtained from social media corporations, research has relied on retrospective estimates of time spent via self-report questionnaires. Time-use-diaries, which may be subject to less recall and response bias, might offer an alternative approach.^15^ Time-use-diaries ask participants to recount small time windows (eg, 10 minutes) and allow the summation of total time engaging with specific activities as well as investigation into the time of day these activities occur.^16^ Time-use-diaries could thus complement, and extend more frequently used self-report measures.

Given the preventable inequalities in tobacco use, related diseases, and deaths, another area which warrants investigation is understanding how the relationship between time spent on social media and adolescent nicotine-related product use may vary across different socioeconomic groups. In line with the differential susceptibility pathway, it is plausible that social media could produce greater increases in nicotine-related product use in those more socioeconomically disadvantaged (compared to those more advantaged), which may result in a widening of health inequalities.^15,17^

For policymakers to make informed decisions on social media regulation and guidance, more accurate assessments of these relationships are required. We aimed to estimate the effect of time spent on social media (assessed via self-report questionnaire and time-use-diary) at 14 years on the risk of cigarette, e-cigarette, and dual use at 17 years using the UK-representative Millennium Cohort Study (MCS). We also examined if the effects of social media differed by socioeconomic circumstance (SEC), using the highest parental education as a proxy measure.

## Materials and Methods

We followed the strengthening the reporting of observational studies in epidemiology (STROBE) guidance,^18^ and a published statistical analysis plan^19^ developed with input from a Policy Advisory Group (members included patient/public representatives and stakeholders from policy, non-governmental, and academic sectors); with deviations reported in Supplementary Appendix-A.

### Study Characteristics

**Figure 1.**
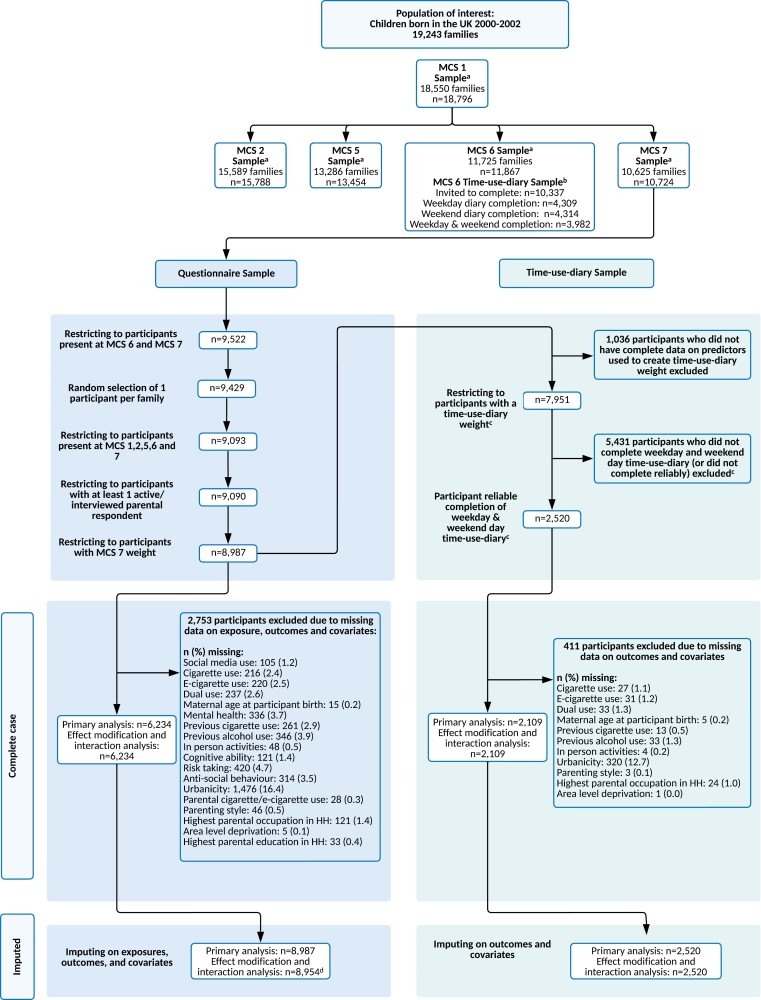
STROBE study flow diagram. ^a^MCS weights used to extrapolate back to population of interest. ^b^ Time-use-diary weight created to extrapolate back to MCS 6 entire sample and combined with MCS 7 weight to extrapolate to population of interest. All predictors used to create time-use-diary weight were existing confounders to be used in analysis. ^c^ Participants who had ≥ 5 ‘no activity recorded’ slots on a weekday, weekend, or both days were deemed as having unreliable diary accounts. ^d^ To facilitate the inclusion of interaction between social media use and highest parental education in the imputation model for the effect modification and interaction sample, *n* = 33 with missing data on highest parental education were excluded prior to imputation. HH = Household; *n* = Number of participants, and MCS = Millennium Cohort Study.

The MCS is a UK-representative cohort study of children born between September 2000 and January 2002.^20^ Families were selected through child benefit records, and contacted via opt-out letters from the Department for Work and Pensions. To over-represent children living in Wales, Scotland and Northern Ireland, disadvantaged areas, and areas with high proportions of ethnic minority groups (in the case of England), a disproportionately stratified clustered sampling design was used.^20^ This study used data for participants and their caregivers who were present in the initial survey (when participants were approximately 9 months old (*n* = 18 796)), and subsequently when participants were 3 (response rate: 78.0%), 11 (69.1%), 14 (76.3%), and 17 years of age (74.6%).^20–22^ Triplet households were excluded. Where households contained two participants, one was randomly selected for inclusion in the analysis (Figure 1). Data were downloaded from the UK Data Service, Universities of Essex and Manchester (October 2021–January 2022). Ethics approval was granted for the MCS surveys; no further approval was required for the current analysis.^20–22^ Further information on the MCS is available from: http://www.cls.ioe.ac.uk/mcs.

### Measures

#### Outcomes

At 17 years (data collected 2018) participant cigarette and e-cigarette use was assessed via a self-report online questionnaire.

##### Cigarette Use

Participants were asked to select one of the six statements which best described their smoking status: “I have never smoked cigarettes,” “I have only ever tried smoking cigarettes once,” “I used to smoke sometimes but I never smoke a cigarette now,” “I sometimes smoke cigarettes now, but I do not smoke as many as one a week,” “I usually smoke between one and six cigarettes a week” and “I usually smoke more than six cigarettes a week.” A dichotomous outcome variable was generated: “never smoked or tried cigarettes once” and “current or former cigarette use” due to low frequencies in categories representing current and former use (questionnaire imputed sample: 22.2% and 7.0%; time-use-diary imputed sample: 19.0% and 6.2%).

##### E-cigarette Use

Similar to the variable recording cigarette use, participants could select one of the six statements which best described their use of e-cigarettes: “I have never tried an e-cigarette or vaping device,” “I have only ever tried an e-cigarette or vaping device once,” “I used to use an e-cigarette or vaping device sometimes, but I never use an e-cigarette or vaping device now,” “I sometimes use an e-cigarette or vaping device now, but I don’t use an e-cigarette or vaping decide as often as one a week,” “I usually use an e-cigarette or vaping device between one and six times a week,” “I usually use an e-cigarette or vaping device more than six times a week.” Similar to cigarette use, responses were collapsed into a dichotomous variable with categories: “never used an e-cigarette or tried once” and “current or former e-cigarette use,” due to low frequencies in categories representing current and former use (questionnaire imputed sample: 11.7% and 11.2%; time-use-diary imputed sample: 10.7% and 9.7%).

##### Current Dual Use of Cigarettes and E-cigarettes

A composite variable was generated with categories: “never used both cigarettes or e-cigarettes or tried once,” “current or former cigarette or e-cigarette user,” and “current dual user.”

#### Exposures

At 14 years (data collected 2015) participant time spent on social media was assessed via a self-report questionnaire and a time-use-diary.

##### Time Spent on Social Media on a Normal Weekday During Term Time

Participants were asked “on a normal weekday during term time, how many hours do you spend on social networking or messaging sites or apps on the internet such as Facebook, Twitter, and WhatsApp?” via a self-report online questionnaire. Participants were given eight options to select from, ranging from “no social media use” to “≥7 hours.” Due to low frequencies in the higher time categories, data were collapsed into the following: “no social media use,” “1–<30 minutes,” “30 minutes–<1 hour,” “1–<2 hours,” and “≥2 hours.” For the primary analyses, “1–<30 minutes” was used as the reference category, based on the threshold of potential harm in comparable studies,^23^ and because non-users are likely to be highly atypical (in 2022, 91% of 12–15-year-old UK adolescents used social media, increasing to 97% in adolescents aged 16–17 years).^24^

##### Average Time Spent on Social Media Across a Normal Weekday and Weekend Day

The time-use-diary was completed by participants for two 24-hour periods (one randomly selected weekday and weekend day, either during term time, or during school holidays) when participants were 14 years old (data collected 2015). Participants could complete the diary via an online web form, a mobile/tablet application or a paper form, and were asked to complete the diary in real time (where possible). They could select 1 of the 44 activities for each 10-minute activity slot (144 activity slots within 24 hours); thus, the diary did not allow for multitasking. Social media use was assessed via the activity code “browsing and updating social networking sites (eg, Twitter, Facebook, BBM, and Snapchat).”^25^ Further detail on time-use-diary completion, can be found in the MCS time-use-diary documentation.^25^ Adopting a similar approach to Atkin et al.,^26^ diaries with ≥5–10-minute activity slots with “no activity” were excluded as these were deemed unreliable, as were participants who did not provide data on both a weekday and weekend day. Using the time-use-diary data a variable representing average time spent on social media across a weekday and weekend day was generated, categorized as: “no social media use,” “1–<30 minutes” (reference category), “30 minutes–<1 hour,” “1–<2 hours,” and “≥2 hours.”

#### Confounders

**Figure 2.**
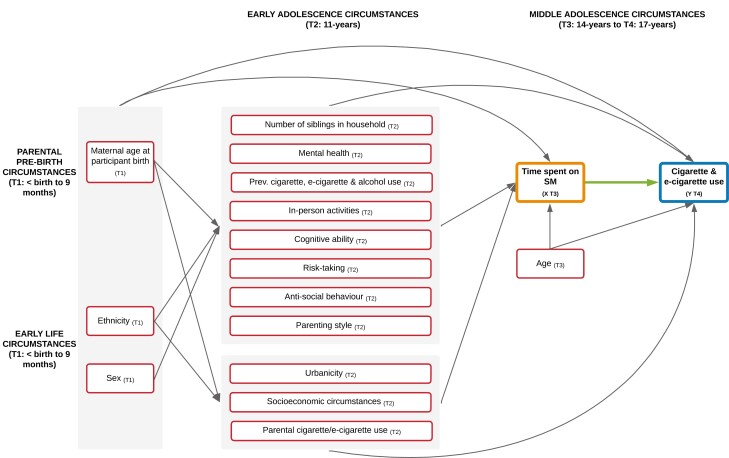
Saturated directed acyclic graph illustrating the hypothesized relationship between social media use at 14 years and cigarette and e-cigarette use at 17-years-old and the minimal sufficient adjustment set. Observed confounders: Rectangular red node (includes confounders where proxy variables are used). Exposure X: Orange node. Outcome Y: Blue node. Green arrow indicates focal relationship under investigation. Inward and outward arrows from gray-shaded areas pertain to all nodes within the shaded area. Confounders (information on the specific variables used to represent each confounder can be found in Supplementary Appendix B): Parental pre-birth and early life circumstances (T1: < birth to 9 months): Maternal age at participant birth, ethnicity (6-category Census class), and sex. Early adolescence circumstances (T2: 11 years): Number of siblings of participant in household, mental health (Strengths and Difficulties Questionnaire Total Difficulties), previous alcohol use, previous cigarette use (also used as a proxy measure for previous e-cigarette use), in-person activities, cognitive ability (British Ability Scales II Verbal Similarities), risk-taking (Cambridge Gambling Task), antisocial behavior, urbanicity (Office for National Statistics Rural Urban Classification), parenting style, parental cigarette use (also used as a proxy measure for parental e-cigarette use), and socioeconomic circumstances (household income [Organization for Economic Co-operation and Development Income Equivalised Quintiles], family structure, parental occupation [National Statistics Socioeconomic Classification], area-level deprivation [Indices of Multiple Deprivation], and parental education [National Vocational Qualification]). Middle adolescence circumstances (T3: 14 years): Age. Not shown: Baseline cigarette use (T3: 14 years), baseline e-cigarette use (T3: 14 years) and previous social media use (T2: 11 years) adjusted for in sensitivity analyses. Socioeconomic circumstances are not included in adjustment set for effect modification and interaction analysis models. SM = Social media, and T-= Timepoint.

We prepared directed acyclic graphs, used to identify confounding variables that require conditioning when estimating causal effects, with support from our Policy Advisory Group, subject knowledge, and the existing evidence base.^27^ The directed acyclic graphs produced highlighted our assumptions regarding the causal relationship between variables of interest, and informed our statistical approach. The DAG presented in Figure 2 presents the minimally sufficient adjustment set, identified using DAGitty software. Confounders included measured pre-birth (ie, maternal age at participant birth), early life (ie, ethnicity), early adolescence (ie, mental health, cognitive ability, and SEC), and mid-adolescence circumstances (ie, age).

#### Socioeconomic Circumstance as an Effect Modifier

Parental education was used as proxy measure for SEC as it is relatively stable over time, it is strongly correlated with health behaviors, and is related to other measures of SEC (eg, income).^28,29^ Using the highest National Vocational Level of both parents in the household (where relevant) when the participant was 11 years old (data collected 2012), a dichotomous variable was generated representing “high parental education” (ie, International Standard Classification of Education [ISCED] 3 or English A/AS/S levels or higher) and “low parental education” (ie, ISCED 2 or English O level/General Certificate of Secondary Education [GSCE] grades A–C or lower).

Supplementary Appendix-B provides details on all variables, their measurement, original format, and treatment within this study.

## Statistical Analysis

Descriptive statistics explored the association between social media (14 years) and cigarette, e-cigarette, and dual use (17 years), and confounders. MCS weights accounted for the clustered sampling design and attrition. Weights were created for the time-use-diary analyses (Supplementary Appendix C). Statistical analysis was performed using *Stata.V16.*

### Effect of Social Media Use on Cigarette, E-cigarette, and Dual Use (Primary Analysis)

Within imputed samples, odds ratios (ORs) were estimated using logistic regression to examine the association between social media use and the binary outcomes of cigarette and e-cigarette use, before and after adjusting for confounding. Relative risk ratios were estimated using multinomial logistic regression for dual use.

#### Additional/Sensitivity Analyses

Analyses were repeated in complete case samples and stratified by sex. We conducted sensitivity analyses using three-category cigarette and e-cigarette use variables, with current and former users separated. We compared findings from the time-use-diary to the questionnaire exposure variable by limiting it to weekday social media use. To further account for possible reverse causation, we adjusted for cigarette and e-cigarette use at 14 years. These were not included in the primary analysis since they may sit on the causal pathway and therefore represent an overadjustment.

### Differential Effect of Social Media Use on Cigarette and E-cigarette Use by Socioeconomic Circumstances

Within imputed samples, to examine if parental education might buffer against the risk of social media use (on a normal weekday [assessed via questionnaire] and across a normal weekday and weekend day [assessed via time-use-diary]) on cigarette and e-cigarette use, effect measure modification was assessed. This was achieved by calculating risk differences (RD’s; absolute differences in cigarette/e-cigarette use by social media time category, within high and low parental education groups [baseline: “high parental education”]). We used linear regression with robust standard errors^30^ which accurately estimates RDs when modeling binary outcomes.^31^ We opted to examine effect measure modification on an additive scale since it is considered of greater public health relevance.^32^

#### Additional/Sensitivity Analyses

As per recommendations from Knol and VanderWeele,^32^ and the STROBE guidance^18^ interaction was also assessed, where “high parental education and no social media use” served as the baseline (stratum with the lowest risk of cigarette or e-cigarette use); interactions and effect modification are statistically equivalent but present the results in a different way.^30^ Analyses were repeated using risk ratios (estimated in Poisson regression models, with robust standard errors), which assess effect modification and interaction on the multiplicative scale (Supplementary Appendix D)^33^￼ and using complete case samples.

The reference category of “1–<30 minutes” used when assessing the effect of social media use on cigarette, e-cigarette, and dual use (in the primary analysis) was not used when assessing the differential effect of social media use on cigarette, e-cigarette, and dual use. Instead, the reference category of “no social media use” was used, as per the recommendations from Knol and VanderWeele,^30^ which recommend using the reference category with the lowest risk of the outcome under study.

### Imputation

Multiple imputations by chained equations were performed in 20 datasets, under a missing-at-random assumption.^34^ Estimates were combined using Rubin’s rules.^35^ Imputation models were performed separately for each exposure, as they have different samples and to accommodate different weights (Supplementary Appendix C). Models included relevant outcomes, confounders, and variables used to account for sample design and attrition to 17 years. For effect modification and interaction samples, models included an interaction between social media use and parental education. Supplementary Appendix E details the regression models used for imputation.

## Results

The final imputed questionnaire sample consisted of 8987 participants (69.4% [*n* = 6234] complete data), and the final imputed time-use-diary sample consisted of 2520 participants (83.7% [*n* = 2109] complete data).

In the questionnaire imputed sample, 28.9% of participants were cigarette users, 23.7% were e-cigarette users, and 8.2% were dual users (Supplementary Appendix F). Prevalences were similar in the time-use-diary imputed sample (25.3%, 21.0%, and 7.2%, respectively). Generally, the prevalence of cigarette use was similar for males and females, though males were more likely to report e-cigarette and dual use. The proportion of social media non-users was considerably smaller for the questionnaire measure (8.4%) compared to the time-use-diary measures (weekday use: 63.8% and average use across weekday and weekend: 49.0%).

### Effect of Social Media Use on Cigarette, E-cigarette, and Dual Use

Questionnaire-reported time spent on social media on a normal weekday was associated with increased risk of cigarette, e-cigarette, and dual use in a dose–response manner (Table 1 and Supplementary Appendix G, Table-G1). Those who used social media for ≥ 2 hours/day (vs. 1–<30 minutes) were at a greater risk for cigarette (adjusted OR [AOR] 2.76 [95% CI: 2.19 to 3.48]), e-cigarette (AOR 3.24 [2.59 to 4.05]), and dual use (adjusted RRR [ARRR] 4.11 [2.77 to 6.08]). No meaningful sex differences were identified (Supplementary Appendix G, Table-G1).

Considering time-use-diary data, average time spent on social media across a weekday and weekend day was associated with increased risk of cigarette and e-cigarette use. In several cases, this was consistent with a dose–response relationship, though estimates were slightly weaker when compared to questionnaire data (Table 1 and Supplementary Appendix G, Table-G2). Those who used social media for ≥ 2 hours (vs. 1–<30 minutes) were at a greater risk of cigarette (AOR 2.63 [1.68 to 4.12]) and e-cigarette use (AOR 1.77 [1.07 to 2.93]). The effect of time spent on social media on risk of dual use was confined to the higher time categories, where social media use for 30 minutes–<1 hour was not associated with an elevated risk of dual use (ARRR 1.42 [0.71 to 2.86]), while use for 1–<2 hours (2.24 [1.14 to 4.41]) and ≥ 2 hours (2.37 [1.18 to 4.76]) were.

**Table 1.** The Relationship Between (A) Time Spent on Social Media on a Normal Weekday (Questionnaire) and (B) Average Time Spent on Social Media Across a Normal Weekday and Weekend Day (Time-Use-Diary) With Cigarette, E-cigarette Use (Adjusted Odds Ratios), and Dual Use (Adjusted Relative Risk Ratios)

	Questionnaire imputed sample (*n* = 8987)			Time-use-diary imputed sample (*n* = 2520)		
	Weighted prevalence % (observed *n* with outcome)	OR (95% CI)	AOR (95% CI)^a^	Weighted prevalence % (observed *n* with outcome)	OR (95% CI)	AOR (95% CI)^a^
*A. Current or former cigarette use (ref: Never used cigarette or tried once)*						
No social media use	14.9 (94)	0.83 (0.56 to 1.22)	0.82 (0.57 to 1.18)	23.5 (239)	1.28 (0.93 to 1.76)	1.15 (0.83 to 1.60)
1-<30 min	17.4 (175)	1.00	1.00	19.3 (94)	1.00	1.00
30 min–<1 h	22.9 (275)	1.41 (1.05 to 1.89)	1.48 (1.11 to 1.97)	27.8 (95)	1.60 (1.09 to 2.35)	1.78 (1.22 to 2.60)
1–<2 h	26.9 (395)	1.74 (1.35 to 2.26)	1.78 (1.38 to 2.29)	31.8 (74)	1.95 (1.29 to 2.94)	1.87 (1.23 to 2.84)
≥2 h	37.2 (1445)	2.80 (2.23 to 3.52)	2.76 (2.19 to 3.48)	39.1 (58)	2.67 (1.68 to 4.27)	2.63 (1.68 to 4.12)
*B. Current or former e-cigarette use (ref: Never used e-cigarette or tried once)*						
No social media use	13.9 (88)	1.01 (0.66 to 1.53)	0.94 (0.63 to 1.39)	20.4 (204)	1.19 (0.80 to 1.79)	1.04 (0.71 to 1.51)
1–<30 min	13.8 (157)	1.00	1.00	17.7 (73)	1.00	1.00
30 min–<1 h	20.9 (262)	1.65 (1.24 to 2.20)	1.79 (1.34 to 2.39)	21.5 (72)	1.28 (0.80 to 2.03)	1.54 (1.00 to 2.38)
1–<2 h	22.4 (351)	1.80 (1.42 to 2.29)	2.06 (1.61 to 2.64)	25.2 (62)	1.57 (0.98 to 2.50)	1.56 (1.01 to 2.40)
≥2 h	29.8 (1200)	2.65 (2.14 to 3.29)	3.24 (2.59 to 4.05)	27.0 (46)	1.72 (1.00 to 2.98)	1.77 (1.07 to 2.93)

	Weighted prevalence % (observed *n* with outcome)	RRR (95% CI)	ARRR (95% CI)^a^	Weighted prevalence % (observed *n* with outcome)	RRR (95% CI)	ARRR (95% CI)^a^
*C. Current or former cigarette or e-cigarette use (ref: Never used cigarette or e-cigarette or tried once)*						
No social media use	15.0 (95)	0.82 (0.56 to 1.19)	0.78 (0.55 to 1.12)	22.1 (232)	1.21 (0.83 to 1.74)	1.05 (0.75 to 1.46)
1–<30 min	17.7 (186)	1.00	1.00	19.3 (83)	1.00	1.00
30 min–<1 h	23.8 (302)	1.49 (1.13 to 1.97)	1.58 (1.21 to 2.07)	28.4 (101)	1.68 (1.09 to 2.60)	1.96 (1.31 to 2.93)
1–<2 h	24.8 (383)	1.63 (1.27 to 2.10)	1.74 (1.36 to 2.22)	28.7 (71)	1.82 (1.11 to 2.99)	1.75 (1.10 to 2.78)
≥2 h	33.5 (1350)	2.63 (2.11 to 3.28)	2.79 (2.23 to 3.48)	32.5 (49)	2.19 (1.28 to 3.77)	2.17 (1.34 to 3.52)
*C. Current dual use (ref: Never used cigarette or e-cigarette or tried once)*						
No social media use	4.2 (29)	0.93 (0.46 to 1.88)	0.88 (0.45 to 1.72)	6.9 (67)	1.28 (0.78 to 2.10)	1.19 (0.68 to 2.09)
1–<30 min	4.3 (46)	1.00	1.00	5.7 (28)	1.00	1.00
30 min–<1 h	6.0 (68)	1.52 (0.92 to 2.52)	1.69 (1.03 to 2.77)	6.3 (19)	1.25 (0.63 to 2.47)	1.42 (0.71 to 2.86)
1–<2 h	8.7 (125)	2.35 (1.54 to 3.59)	2.72 (1.79 to 4.13)	10.3 (22)	2.20 (1.21 to 3.99)	2.24 (1.14 to 4.41)
≥2 h	10.5 (405)	3.39 (2.31 to 4.98)	4.11 (2.77 to 6.08)	10.0 (17)	2.26 (1.17 to 4.36)	2.37 (1.18 to 4.76)

Questionnaire imputed sample: *n* = 8987 (weighted sample: *n* = 6175). Time-use-diary imputed sample: *n* = 2520 (weighted sample: *n* = 5005).

^a^Adjusted for sex, ethnicity, parental cigarette use, parental e-cigarette use, parenting style, previous cigarette use, previous e-cigarette use, antisocial behavior, previous alcohol use, urbanicity, age, number of siblings in household, maternal age at participant birth, in-person activities, cognitive ability, mental health, risk-taking, and socioeconomic circumstances (family structure, household income, highest parental education in household, highest parental occupation in household, and area-level deprivation). AOR = Adjusted odds ratio; ARRR = adjusted relative risk ratio; CI = confidence interval; H = hour; Min = minutes; and Ref = reference category.

For cigarette and e-cigarette use associations were potentially stronger for females (Supplementary Appendix G, Table-G2). For example, females who used social media for ≥2 hours had a greater risk of cigarette (AOR 3.62 [2.13 to 6.18] vs. 1.69 [0.63 to 4.51]) for males) and e-cigarette use (2.21 [1.21 to 4.05] vs. 1.50 [0.58 to 3.88] for males). For dual use, general estimates were stronger for females, with one exception, where the effect of social media use for 1–<2 hours was considerably higher in males (ARRR 3.60 [1.24 to 10.4] vs. 1.71 [0.67 to 4.34] for females), albeit with wide confidence intervals.

#### Additional/Sensitivity Analyses

Analyses repeated in complete case samples showed similar estimates to those in imputed samples; however, on occasion were slightly larger (Supplementary Appendix G, Tables-G3-G4). When separating current and former cigarette and e-cigarette users, generally the patterning of results was similar to when examining current and former users jointly (Supplementary Appendix G, Table-G5).

A comparison of questionnaire and time-use-diary measures of time spent on social media on a normal weekday, revealed smaller estimates for the time-use-diary measure compared to the questionnaire (Supplementary Appendix G, Table-G6).

Following additional adjustment for baseline outcome measures, estimates were similar or only slightly weaker than those without baseline adjustment, with dose–response relationships generally persisting (although not always in the time-use-diary data; Supplementary Appendix G, Tables-G7-G8).

### Differential Effect of Social Media Use on Cigarette and E-cigarette Use by Socioeconomic Circumstances

The effects of questionnaire-reported social media use were generally larger in the higher parental education groups (vs. lower), despite non-users in high parental education groups reporting a lower baseline prevalence of cigarette and e-cigarette use (Table 2 and Supplementary Appendix H, Table-H1). For example, the risk of cigarette use among 30 minutes–<1 hour/day users (vs. non-users) with low parental education were smaller than those with high (adjusted risk differences 1.4% [−9.2% to 11.9%] vs 12.4% [6.9% to 18.0%]). In other words, the absolute difference in the adjusted risk differences between these two groups (ie, the measure of additive effect measure modification) was −11.1% (−22.7% to 0.5%), indicating modification on the additive scale. Similar findings were observed for those who used social media for 1–<2 hours/day (additive effect modification measure: −11.6% [−23.0% to −0.1%]) and ≥ 2 hours/day (additive effect modification measure: −10.5% [−21.3% to 0.3%]). Patterns for e-cigarette use in the higher time categories also showed greater effects in the high parental education groups; however, were considerably less pronounced with wide confidence intervals (Table 2 and Supplementary Appendix H, Table-H2).

**Table 2.** Participant (1) Cigarette and (2) E-cigarette Use According to Time Spent on Social Media, Within Strata of Parental Education Within the Questionnaire Imputed Sample (*n* = 8954)

	(1) Current or former cigarette use		(2) Current or former e-cigarette use	
	High parental education	Low parental education	High parental education	Low parental education
*Weighted prevalence % (observed n with outcome/without outcome)*				
No social media use	10.1 (43/391)	22.1 (49/246)	9.4 (43/391)	18.0 (42/253)
1–<30 min	13.0 (95/617)	23.4 (78/352)	9.6 (75/636)	20.4 (81/349)
30 min–<1 h	23.7 (173/651)	22.8 (99/402)	19.3 (152/672)	25.3 (109/393)
1–<2 h	26.8 (237/701)	27.0 (154/465)	19.3 (188/751)	25.1 (160/459)
≥2 h	36.8 (768/1484)	38.6 (677/1272)	27.8 (590/1662)	31.7 (607/1343)
*Unadjusted RD (95% CI; p-value) for time spent on social media within strata of parental education* ^a^				
No social media use	Ref	Ref	Ref	Ref
1–<30 min	2.9 (−1.9 to 7.8; 0.239)	1.3 (−12.1 to 14.7; 0.848)	0.3 (−4.3 to 4.8; 0.913)	2.5 (−8.3 to 13.2; 0.652)
30 min–<1 h	13.6 (7.7 to 19.4; <0.0001)	0.6 (−11.8 to 13.0; 0.921)	9.9 (4.9 to 14.9; <0.0001)	7.4 (−3.5 to 18.2; 0.182)
1–<2 h	16.7 (12.1 to 21.3; <0.0001)	4.9 (−7.6 to 17.4; 0.442)	10.0 (5.2 to 14.7; <0.0001)	7.2 (−3.9 to 18.3; 0.203)
≥2 h	26.7 (22.0 to 31.4; <0.0001)	16.4 (5.0 to 27.8; 0.005)	18.5 (14.2 to 22.8; <0.0001)	13.8 (3.6 to 23.9; 0.008)
*Unadjusted measure of additive effect modification (95% CI; p-value)* ^b^				
No social media use	Ref		Ref	
1–<30 min	−1.6 (−15.8 to 12.6; 0.823)		2.2 (−9.1 to 13.5; 0.701)	
30 min–<1 h	−12.9 (−26.3 to 0.5; 0.059)		−2.5 (−14.2 to 9.1; 0.669)	
1–<2 h	−11.8 (−25.1 to 1.4; 0.081)		−2.8 (−14.7 to 9.2; 0.647)	
≥2 h	−10.3 (−22.4 to 1.9; 0.097)		−4.7 (−15.7 to 6.3; 0.399)	
*Adjusted* ^c^ *RD (95% CI; p-value) for time spent on social media within strata of parental education* ^a^				
No social media use	Ref	Ref	Ref	Ref
1–<30 min	2.0 (−2.6 to 6.6; 0.386)	2.6 (−8.4 to 13.5; 0.646)	−0.6 (−5.0 to 3.7; 0.772)	3.0 (−5.8 to 11.9; 0.501)
30 min–<1 h	12.4 (6.9 to 18.0; <0.0001)	1.4 (−9.2 to 11.9; 0.799)	9.6 (4.6 to 14.6; <0.0001)	9.8 (0.4 to 19.3; 0.041)
1–<2 h	14.9 (10.5 to 19.3; <0.0001)	3.4 (−7.5 to 14.3; 0.541)	10.4 (5.6 to 15.2; <0.0001)	8.1 (−1.6 to 17.9; 0.101)
≥2 h	24.6 (20.0 to 29.2; <0.0001)	14.2 (3.9 to 24.5; 0.007)	21.6 (17.4 to 25.8; <0.0001)	18.0 (9.0 to 27.0; <0.0001)
*Adjusted* ^c^ *measure of additive effect modification (95% CI; p-value)* ^b^				
No social media use	Ref		Ref	
1–<30 min	0.5 (−11.2 to 12.2; 0.929)		3.7 (−5.7 to 13.0; 0.441)	
30 min–<1 h	−11.1 (−22.7 to 0.5; 0.061)		0.2 (−9.8 to 10.3; 0.965)	
1–<2 h	−11.6 (−23.0 to −0.1; 0.048)		−2.3 (−12.4 to 7.9; 0.662)	
≥2 h	−10.5 (−21.3 to 0.3; 0.057)		−3.6 (−13.2 to 6.1; 0.463)	

Questionnaire imputed sample: *n* = 8954 (weighted sample: *n* = 6976).

^a^RDs represent the absolute difference in current or former cigarette or e-cigarette use by time spent on social media, within the low and high parental education groups (eg, in the high parental education group, the RD of 2.9, indicates the absolute difference in cigarette use for participants who used social media for 1–<30 minutes compared to those who did not use social media).

^b^Measure of effect modification on an additive scale represents the size of the absolute difference between the RDs for participant current or former cigarette or e-cigarette use by time spent on social media, within the low parental education group compared with baseline (high parental education group; eg, for participants who used social media for 1–<30 minutes [compared to those who did not use social media], the measure of effect modification on the additive scale [−1.6] represents the absolute difference between the RDs for those within the low parental education group [RD = 1.3] compared with the high parental education group [RD = 2.9]).

^c^Adjusted for: ethnicity, sex, parental cigarette use, parental e-cigarette use, parenting style, previous cigarette use, previous e-cigarette use, antisocial behavior, previous alcohol use, urbanicity, age, number of siblings in household, maternal age at participant birth, in-person activities, cognitive ability, mental health, and risk-taking. Values may not add up due to rounding. CI = confidence interval; H = hour; Min = minute; *n* = number of participants; RD = risk differences, and Ref = reference category.

The risk of cigarette use for 1–<30-minute users (vs. non-users), based on time-use-diary data, was 20.4% (−31.1% to −9.8%) lower among participants with low parental education versus high (Supplementary Appendix H, Table-H1). For ≥2 hours use, the opposite was seen—effects were larger for those with low parental education versus high (13.9% [−1.2% to 29.1%] vs. 10.2% [−1.7 to 22.0]; additive effect modification measure: 3.7% [−14.8% to 22.3%]). For e-cigarette use, no discernible patterns were observed (Supplementary Appendix H, Table-H2).

#### Additional/Sensitivity Analyses

Investigation of interaction effects produced consistent findings (Supplementary Appendix H, Tables-H1-H2). Analyses repeated on complete case samples, generally revealed similar findings (Supplementary Appendix H, Tables-H3-H4). Analyses using risk ratios showed no evidence of effect modification on the multiplicative scale (Supplementary Appendix H, Tables-H5-H8).

## Discussion

### Summary of Findings

In a UK-representative cohort, adolescent social media use at 14 years may increase the risk of cigarette, e-cigarette, and dual use at 17 years in a dose–response manner. These findings persisted in a series of analyses to examine a range of biases including missing data and reverse causation.

The influence of social media use on cigarette use appeared to be stronger for social media users (vs. non-users) with high compared to low parental education. However, this was driven by differences in the prevalence of cigarette use in non-users (high parental education: 10.1% and low parental education: 22.1%) as opposed to the higher time categories where the risk of cigarette use was very similar (36.8% and 38.6% in the high and low parental education groups, respectively). This implies the protective effects of low social media use may be greater in the higher parental education group, as opposed to the harmful effects of high social media use. These associations were robust to adjustment for confounders and while the patterning of results for the e-cigarette use was relatively similar, the degree of modification was smaller.

### Comparison With Other Findings

The current analysis corroborates the finding that increased social media use is associated with cigarette and e-cigarette use.^11,–14,16^ A recent systematic review identified frequent social media use (studies = 8) and exposure to tobacco marketer-generated content on social media (studies = 3) increased likelihood of tobacco use.^36^ While exposure to e-cigarette marketer-generated content was associated with increased use of e-cigarettes (studies = 4).^36^ Limitations highlighted, and addressed in our study, include failure to assess dual use, insufficient adjustment for confounding, and a lack of longitudinal analyses, hindering the ability to assess the potential for reverse causality.^36^ Moreover, no study sought to explore if relationships differed by socioeconomic circumstance (SEC), and thus may widen inequalities.^36^

Existing research, mainly conducted in the United States, demonstrates increased cigarette and e-cigarette use among adolescents who spend time on social media and/or report exposure to social media cigarette or e-cigarette-related marketing,^37,38^ with similar findings observed in China, France, New Zealand, and Thailand.^39–42^ The generalizability of these studies to the UK is impeded by differences in cigarette/e-cigarette social media marketing regulations, the legal age of consumption, and cigarette, e-cigarette, and social media use prevalence.^43^

The UK-based research is limited to the cross-sectional analysis of data from multiple countries participating in the Health Behavior in School-aged Children Survey (where UK-specific estimates are not reported)^44,45^ and one study which explicitly examines UK adolescents using the MCS.^10^ This latter study found social media use for ≥1–<5 hours/day and ≥5 hours/day (vs. <1 hour/day) at 14 years was associated with increased odds of smoking at 17 years (AOR 1.38 [1.05 to 1.81] and 1.91 [1.41 to 2.59], respectively).^10^ This study did not investigate e-cigarette or dual use. The growing body of evidence illustrating the positive associations between e-cigarette and dual use, and substance use and poor academic performance, and the increased (often unregulated) marketing of nicotine-related products on social media, highlights the need to understand social media’s role as a potential risk factor for e-cigarette and dual use.^46^

In our analysis of the same dataset, we adjusted for a wider range of confounding factors not considered in this previous study including in-person activities, risk-taking and parenting style, estimated impacts on e-cigarette and dual use, explored the potential for reverse causation, and considered potential impacts on health inequalities.

### Strengths and Limitations

We adjusted for a comprehensive range of confounders, informed by the creation of directed acyclic graphs. The potential for reverse causality was investigated, finding effects generally persisted when accounting for baseline measures of our outcomes. Our investigation of parental education as an effect modifier helps to understand the inequalities in social media harms which is important given that adolescents from less disadvantaged backgrounds are at a greater risk of cigarette and e-cigarette use.^47,48^

While we aimed to implement the best possible analyses, there are issues intrinsic to the data, which must be considered. The MCS limited its assessment of social media to time spent and did not consider other aspects (eg, exposure to nicotine-related content) or social media characteristics (eg, platform, type of content viewed). Although we used time-use-diaries to address the potential recall bias in the questionnaire measure, time-use-diary completion was low (38.5%), resulting in a small sample which was potentially less representative. The failure of the time-use-diary to capture multi-tasking, its potential completion during the school holidays, and its possible retrospective completion may have influenced reporting and consequently resulted in lower reported levels of social media use compared to the questionnaire measure (findings mirrored in previous research using the time-use-diary).^16,49^ This underestimation may, in part, explain the weaker associations observed in the time-use-diary data. However, it is impossible to verify this in the absence of a gold standard measure. Using multiple devices to holistically track social media use over multiple days could help to overcome these issues, though achieving this with population-representative cohorts could be resource-intensive. Exposure and outcome measures, although completed individually with confidentially emphasized, were self-reported, thus social-desirability bias remains possible.

Although we adjusted for numerous confounders, the potential for residual or unmeasured confounding remains. Despite including indicators for all proposed confounders as far as the data allow, there may be some not fully represented by our set of measured variables and others which we have not identified. This could lead to bias of unclear direction, substantially affecting the results. Furthermore, we recognize although dose–response relationships were observed, their presence may have arisen from confounding.^50^

### Implications for Policy, Practice, and Further Research

Our findings suggest that the potentially negative effects of social media use on cigarette use are greater among more advantaged groups. However, confidence intervals were wide, reducing the ability to draw definitive conclusions. Furthermore, unmeasured or residual confounding may be a concern here. For example, we found cigarette use prevalence among social media non-users was far lower in those whose parents had high (vs. low) academic qualifications. The reasons behind nonuse of social media, which may range from parenting strategies to the availability of relevant resources (eg, device access) likely vary across socioeconomic groups and could have different implications for health behaviors. Future research should explore this further. The use of more accurate validated social media measures to determine the degree to which causal relationships between specific aspects of social media use, such as exposure to nicotine-related products, and cigarette, e-cigarette, and dual-use exist should be considered. This could include the identification of the relative contributions of exposure to user and marketer-generated content, which would support the implementation of targeted policies and interventions.

Due to the rapid adoption of social media in adolescents, and the benefits it can present (eg, increased social capital, identity, and aspirational development),^6^ it may be a critical environment in which to intervene through online health interventions. For example, nicotine-prevention messages to prevent or stop nicotine product use may help tobacco control in adolescents. Yet, it is acknowledged the current lack of appropriate regulation of harmful nicotine-related social media content may undermine such positive public health messaging. Overall, our findings strengthen calls for guidance on time spent on social media and restrictions on nicotine-related content (including commercial/influencer marketing and user-generated content) on social media. Importantly, there is a need for increased awareness of the algorithms driving adolescent exposure to such content on social media, thus facilitating their interrogation and redesign to ensure they function in a way which best serves adolescents.

## Conclusion

Adolescent social media use for ≥30 minutes daily might increase risk of cigarette, e-cigarette, and dual use and this risk may increase in a dose–response manner. Guidance on time spent on social media, legislation securing adolescent online safety, including regulation of nicotine-related content on social media should be prioritized.

## Supplementary Material

Supplementary material is available at *Nicotine and Tobacco Research* online.

ntae057_suppl_Supplementary_Appendix

## Data Availability

Original MCS data are held by the UK data Service and are available on request from (https://ukdataservice.ac.uk/). Datasets accessed are listed below: Millennium Cohort Study: First Survey, 2001-2003- DOI: 10.5255/UKDA-SN-4683-5. Millennium Cohort Study: Second Survey, 2003-2005- DOI: 10.5255/UKDA-SN-5350-5. Millennium Cohort Study: Fifth Survey, 2012- DOI: 10.5255/UKDA-SN-7464-5. Millennium Cohort Study: Sixth Survey, 2015- DOI: 10.5255/UKDA-SN-8156-7. Millennium Cohort Study: Seventh Survey, 2018- DOI: 10.5255/UKDA-SN-8682-2. The analytic code is available in an online public repository: https://doi.org/10.5281/zenodo.7664236°https://github.com/AmritKPurba/Social_media_healthrisk_behaviours

## Transparency

The lead author (AKP) affirms that the manuscript is an honest, precise, and transparent account of the study reported, with no important aspects omitted. Any discrepancies from the study as planned (and, published in the statistical analysis plan) have been explained.

## Dissemination to Participants and Related Patients and Public Communities

The study findings will be disseminated via conference presentations, press releases, and social media outlets. The authors will disseminate findings to media organizations and press councils, and also national health organizations that are instrumental in the development of social media recommendations for adolescents. Additionally, the study findings will be disseminated via advisory group members working across academic, governmental, and charity sectors.
