# Supplementary material for: The Relationship Between Time Spent on Social Media and Adolescent Cigarette, E-cigarette, and Dual Use: A Longitudinal Analysis of the UK Millennium Cohort Study
Source: Nicotine Tob Res. 2024 Apr 4;27(4):693–704. doi: 10.1093/ntr/ntae057 (PMC11931205; doi:10.1093/ntr/ntae057)
Supplement: ntae057_suppl_Supplementary_Appendix [file ntae057_suppl_supplementary_appendix.docx]

# Supplementary appendix to

# The relationship between time spent on social media and adolescent cigarette, e-cigarette and dual use: a longitudinal analysis of the UK Millennium Cohort Study

Amrit Kaur Purba (PhD)

Marion Henderson (PhD)

Andrew Baxter (PhD)

Anna Pearce (PhD)

S Vittal Katikireddi (PhD)

# Table of contents

[APPENDIX-A. Deviations from the published statistical analysis plan 2](#_Toc137132993)

[APPENDIX-B. Variables used in analysis 3](#_Toc137132994)

[APPENDIX-C. Weights used in analyses 13](#_Toc137132995)

[Questionnaire analyses 13](#_Toc137132996)

[Time-use-diary analyses 13](#_Toc137132997)

[APPENDIX-D. Differential effect of social media on cigarette and e-cigarette use: effect modification and interaction analyses 16](#_Toc137132998)

[Effect modification: association between social media use and cigarette and e-cigarette use, according to strata of parental education 16](#_Toc137132999)

[Interaction: risk of cigarette and e-cigarette use according to ‘combinations’ of social media use and parental education 16](#_Toc137133000)

[Additional/sensitivity analyses 16](#_Toc137133001)

[APPENDIX-E. Variables included within imputation models 18](#_Toc137133002)

[APPENDIX-F. Characteristics of imputed and complete case samples 21](#_Toc137133003)

[APPENDIX-G. Effect of social media use on cigarette, e-cigarette and dual use 27](#_Toc137133004)

[APPENDIX-H. Differential effect of social media use on cigarette and e-cigarette use by socioeconomic circumstance 41](#_Toc137133005)

[Assessment on the additive scale using risk differences (RDs) 41](#_Toc137133006)

[Assessment on the multiplicative scale using risk ratios (RRs) 49](#_Toc137133007)

[REFERENCES 57](#_Toc137133008)

# APPENDIX-A. Deviations from the published statistical analysis plan

We planned to create a continuous variable, representing the average time spent on social media across a weekday and weekend day (assessed via time-use-diary) adopting a fractional polynomial approach. Following creation of the fractional polynomial, hypothesis tests comparing the fractional polynomial models with the linear model showed no significant differences. When categorised, a dose-response relationship was observed, suggesting some benefit of adopting a categorical variable, thus this variable was treated as categorical in all analyses.

We planned to treat confounders number of siblings of participant in the household, age, and maternal age at participant birth, as continuous variables however due to the absence of a linear relationship with outcomes investigated, in some models these variables were treated as categorical.

For the investigation of effect measure modification and interaction, we anticipated using ‘1 to <30 minutes and high parental education’ as the reference category. However, following assessment, the stratum with the lowest risk of outcomes cigarette and e-cigarette use was ‘no social media use and high parental education’, therefore this was used as the reference category.

We anticipated creating a binary variable to represent highest parental education in the household where National Vocational Qualification (NVQ) level 1, other academic qualifications (incl. Overseas), and none would be classified as ‘low parental education’ and NVQ levels 2,3,4 and 5, ‘high parental education’. Due to low frequencies, the following categorisation was used: low parental education (NVQ level 2, NVQ level 1, other academic qualifications (incl. Overseas), and none) and high parental education (NVQ levels 3,4 and 5).

# APPENDIX-B. Variables used in analysis

Table-B1. Variables used in analysis

| **Variable** | **MCS  sweep (participant age)** | **Ascertainment**  **Data item/survey question** | **Answer/category choices** | **Treatment in current study** |
| --- | --- | --- | --- | --- |
| Time spent on social media on a normal weekday | MCS 6  (14-years) | Self-completion time-use-diary  Time spent browsing and updating social networking sites (e.g., Twitter, Facebook, BBM, Snapchat) on a normal weekday | Activity code  Browsing and updating social networking sites (e.g., Twitter, Facebook, BBM, Snapchat) on a normal weekday | 5-category variable  On an assigned weekday, each participant recorded what they did from 04:00 to 04:00 the following day in 10-minute time slots (144 activity slots within 24 hours). The primary activity for each time slot was selected from a list of 44 predefined activity codes nested within 12 categories. The number of 10-minute activity slots recording social networking site activity on a weekday was summed to give total time (minutes) browsing and updating social networking sites on a normal weekday. The resulting continuous variable was collapsed into no social media use, 1-<30 mins, 30 mins-<60 mins, 1 hr-<2 hrs, and ≥2 hrs social media use. Adopting a similar approach to Atkin et al.^1^, diaries with ≥5 10-minute activity slots with no activity indicated were excluded from the analysis as these were deemed to be unreliable accounts of a complete day's activity. |
| Average time spent on social media across a normal weekday and weekend day | MCS 6  (14-years) | Self-completion time-use-diary  1. Time spent browsing and updating social networking sites (e.g., Twitter, Facebook, BBM, Snapchat) on a normal weekday 2. Time spent browsing and updating social networking sites (e.g., Twitter, Facebook, BBM, Snapchat) on a normal weekend day | Activity code  Browsing and updating social networking sites (e.g., Twitter, Facebook, BBM, Snapchat) on a normal weekday and weekend day | 5-category variable  On an assigned weekday and weekend day, each participant recorded what they did from 04:00 to 04:00 the following day in 10-minute time slots (144 activity slots within 24 hours). The primary activity for each time slot was selected from a list of 44 predefined activity codes nested within 12 categories. The number of 10-minute activity slots recording social networking site activity on a weekday and weekend day was summed to give total time (minutes) browsing and updating social networking; this was then divided by two to give the average time spent across a weekday and weekend day. The resulting continuous variable was collapsed into no social media use, 1-<30 mins, 30 mins-<60 mins, 1 hr-<2 hrs, and ≥2 hrs social media use. Participants with missing data on 1 or both days were classified as missing. Adopting a similar approach to Atkin et al.^1^, diaries with ≥5 10-minute activity slots with no activity indicated were excluded from the analysis as these were deemed to be unreliable accounts of a complete day's activity. |
| Time spent on social media on a normal weekday during term time | MCS 6 (14-years) | Self-completion online questionnaire  Time spent on social media on a normal weekday during term time | No social media use/1-<30 mins/30-<60 mins/1 hr-<2 hrs/2 hrs-<3 hrs/3 hrs-<5 hrs/5 hrs-<7 hrs/≥7 hrs social media use/Don’t know/Don’t wish to answer/Not applicable | 5-category variable  Don't know, don't wish to answer and not applicable responses were coded as missing. Categories 2 hrs-<3 hrs, 3 hrs-<5 hrs, 5 hrs-<7 and ≥7 hrs were collapsed to give ≥2 hrs social media use, with no changes made to the remaining categories. 4-category variable  Don't know, don't wish to answer, and not applicable responses were coded as missing. Categories 1-<30 mins and 30-<60 mins were collapsed to give 1 min-<1 hr social media use, and categories 2 hrs-<3 hrs, 3 hrs-<5 hrs, 5 hrs-<7 hrs and ≥7 hrs were collapsed to give ≥2 hrs social media use, with no changes made to the remaining categories. |
| Cigarette use | MCS 7 (17-years) | Self-completion online questionnaire  Please read the following statements carefully and decide which one best describes you. Do not include electronic cigarettes (e-cigarettes) | I have never smoked cigarettes/I have only ever tried smoking cigarettes once/I used to smoke sometimes but I never smoke a cigarette now/I sometimes smoke cigarettes now, but I don’t smoke as many as 1 a week/I usually smoke between 1 and 6 cigarettes a week/I usually smoke more than 6 cigarettes a week/Don’t want to answer/Don’t know/No answer | Binary variable  Don't know, don't wish to answer, and no answer responses were coded as missing. Variable was dichotomised into never smoked or tried cigarettes once (I have never smoked cigarettes OR I have only ever tried smoking cigarettes once) and current or former cigarette use (I used to smoke sometimes but I never smoke a cigarette now OR I sometimes smoke cigarettes now, but I don’t smoke as many as 1 a week OR I usually smoke between 1 and 6 cigarettes a week OR I usually smoke more than 6 cigarettes a week). 3-category variable  Don't know, don't wish to answer, and no answer responses were coded as missing. Categories were collapsed to give never smoked or tried cigarettes once (I have never smoked cigarettes OR I have only ever tried smoking cigarettes once), former cigarette use (I used to smoke sometimes but I never smoke a cigarette now), and current cigarette use (I sometimes smoke cigarettes now, but I don’t smoke as many as 1 a week OR I usually smoke between 1 and 6 cigarettes a week OR I usually smoke more than 6 cigarettes a week). |
| E-cigarette use | MCS 7 (17-years) | Self-completion online questionnaire  Please read the following statements carefully and decide which one best describes you | I have never tried an e-cigarette or vaping device/ I have only ever tried an e-cigarette or vaping device once/I used to use an e-cigarette or vaping device sometimes, but I never use an e-cigarette or vaping device now/I sometimes use an e-cigarette or vaping device now, but I don’t use an e-cigarette or vaping device as often as 1 a week/I usually use an e-cigarette or vaping device between 1 and 6 times a week/I usually use an e-cigarette or vaping device more than 6 times a week/Don’t want to answer/Don’t know/No answer | Binary variable  Don't know, don't wish to answer and no answer responses were coded as missing. Variable was dichotomised into never used an e-cigarette or tried once (I have never tried an e-cigarette or vaping device OR I have only ever tried an e-cigarette or vaping device once) and current or former e-cigarette use (I used to use an e-cigarette or vaping device sometimes, but I never use an e-cigarette or vaping device now OR I sometimes use an e-cigarette or vaping device now, but I don’t use an e-cigarette or vaping device as often as 1 a week OR I usually use an e-cigarette or vaping device between 1 and 6 times a week OR I usually use an e-cigarette or vaping device more than 6 times a week).  3-category variable  Don't know, don't wish to answer, and no answer responses were coded as missing. Categories were collapsed to give never used an e-cigarette or tried once (I have never tried an e-cigarette or vaping device OR I have only ever tried an e-cigarette or vaping device once), former e-cigarette use (I used to use an e-cigarette or vaping device sometimes, but I never use an e-cigarette or vaping device now) and current e-cigarette use (I sometimes use an e-cigarette or vaping device now, but I don’t use an e-cigarette or vaping device as often as 1 a week OR I usually use an e-cigarette or vaping device between 1 and 6 times a week OR I usually use an e-cigarette or vaping device more than 6 times a week). |
| Dual use of cigarettes and e-cigarettes | MCS 7 (17-years) | See above | See above | 3-category composite variable  Don't know, don't wish to answer, and no answer responses were coded as missing. Cigarette and e-cigarette use variables were collapsed into never used cigarette or e-cigarette or tried once (I have never tried an e-cigarette or vaping device OR I have only ever tried an e-cigarette or vaping device once) AND (I have never smoked cigarettes OR I have only ever tried smoking cigarettes once), current or former cigarette or e-cigarette use (I used to smoke sometimes but I never smoke a cigarette now OR I sometimes smoke cigarettes now but I don’t smoke as many as 1 a week OR I usually smoke between 1 and 6 cigarettes a week OR I usually smoke more than 6 cigarettes a week OR I used to use an e-cigarette or vaping device sometimes, but I never use an e-cigarette or vaping device now OR I sometimes use an e-cigarette or vaping device now, but I don’t use an e-cigarette or vaping device as often as 1 a week OR I usually use an e-cigarette or vaping device between 1 and 6 times a week ORI usually use an e-cigarette or vaping device more than 6 times a week) and dual use (I sometimes use an e-cigarette or vaping device now but I don’t use an e-cigarette or vaping device as often as 1 a week OR I usually use an e-cigarette or vaping device between 1 and 6 times a week OR I usually use an e-cigarette or vaping device more than 6 times a week) & (I sometimes smoke cigarettes now but I don’t smoke as many as 1 a week OR I usually smoke between 1 and 6 cigarettes a week OR I usually smoke more than 6 cigarettes a week) (Cronbach alpha=0.84). |
| Maternal age at participant birth | MCS 1 (9 months)/ MCS 2 (3-years) | Parental interview 1. Respondent age at birth of participant (categorical) 2. Respondent age at birth of participant (continuous) 3. Respondent ID and interview status | Variable 1: 11 to 19/20 to 29/30 to 39/40 plus years/Not applicable/Not known | 4-category variable (used in complete case analyses) Variable 1, not applicable and not known responses, were coded as missing, variable 3 was used to identify mother of participant and a categorical variable was then created representing maternal age at birth of participant. Continuous variable (used in imputed analyses) Variable 1, not applicable and not known responses, were coded as missing, variable 3 was used to identify mother of participant and a continuous variable was then created representing maternal age at birth of participant. |
| Sex | MCS 1 (9 months)/ MCS 2 (3-years) | Parental interview  Participant sex | Male/Female | No alterations made to the existing variable. |
| Age | MCS 6  (14-years) | Parental interview  Participant age at last birthday | 13/14/15 years | No alterations made to the existing variable. |
| Ethnic group | MCS 1 (9 months)/ MCS 2 (3-years)/ MCS 3 (5-years) | Parental interview  Participant ethnic group (6 category Census class) | White/Mixed/Indian/ Pakistani & Bangladeshi/Black or Black British/Other ethnic group (incl. Chinese, Other)/Refusal/Don’t know/Not applicable | 6-category variable (used in complete case questionnaire analysis) Refusal, don’t know, and not applicable responses were coded as missing. No further alterations were made to the existing variable. Binary variable (used in remaining analyses)  Refusal, don’t know, and not applicable responses were coded as missing. Categories were collapsed to give White and Other (Mixed, Indian, Pakistani & Bangladeshi, Black or Black British, or Other ethnic group). |
| SDQ total difficulties | MCS 5 (11-years) | Parental interview  Participant mental health problems (SDQ total difficulties) | Continuous score | Continuous variable  Calculated the total difficulties score made up of 20 items from the conduct disorders, hyperactivity/inattention, emotional and peer problem subscales of the SDQ. For each subscale variable (e.g., conduct disorders), if >2 items were completed by the parent, the scale average was imputed. Participants classified as missing are those who had some subscales completed where ≤2 items were completed or who had no SDQ information. |
| Previous cigarette use (additionally used as proxy for previous e-cigarette use) | MCS 5 (11-years) | Self-completion online questionnaire  Participant ever regularly smoked tobacco products | Yes/No/Refused/Don’t know/Not applicable | Binary variable  Refused, don’t know, and not applicable responses were coded as missing. No further alterations were made to the existing variable. |
| Previous alcohol use | MCS 5 (11-years) | Self-completion online questionnaire  Participant ever had an alcoholic drink | Yes/No/No answer/Not applicable | Binary variable  No answer and not applicable responses were coded as missing. No further alterations were made to the existing variable. |
| Average days/week of in-person activity | MCS 5 (11-years) | Parental interview  1. Days per week participant attends a club or class to do sport or any other physical activity like swimming, gymnastics, football, or dancing 2. Days per week participant attends non club/class physical activities with friends/siblings (not including walking to school) | Variable 1 and 2: ≥5 days a week/4 days a week/ 3 days a week/2 days a week/1 day a week/Less often than once a week/Not at all/Don't know/Refused/Not applicable | Continuous composite variable  Don't know, refused, and not applicable responses were coded as missing. Variables 1 and 2 were recoded to represent number of days of activities per week. For each variable, categories not at all and less often than once a week were combined, as engagement less than once a week would imply no engagement in a week (=0 days), 1 day a week (=1 day), 2 days a week (=2 days), 3 days a week (=3 days), 4 days a week (=4 days). As per Twenge et al.^2^ and following observation of variable distributions, ≥5 days a week was coded as 6 days (the average of 5, 6, and 7 days). The sum of both frequency variables was calculated to create a composite variable representing weekly frequency of in-person activities, with no requirement to standardise as both variables were on the same scale. Cronbach alpha=0.31 (as we were not measuring the same activity explicitly and instead generalising to any in-person activity, a high alpha statistic was not expected). Participants were classified as missing if they had missing data on 1/both variables used to create the composite variable. |
| Cognitive ability | MCS 5 (11-years) | Participant assessment  BAS II Verbal Similarities-verbal reasoning and verbal knowledge | Standardised score (adjusted for age and ability) | No alterations made to the existing variable. |
| Risk-taking | MCS 5 (11-years) | Participant assessment  CGT-risk-taking | Continuous score | No alterations made to the existing variable. |
| Anti-social behaviour | MCS 5 (11-years) | Self-completion online questionnaire  1. Participant ever taken something from a shop without paying for it 2. Participant ever written things or sprayed paint on a building 3. Participant ever been noisy or rude in a public place 4. Participant ever purposely damaged anything in a public place | Variables 1-4:  Yes/No/No answer/Not applicable | Binary composite variable  No answer and not applicable responses were coded as missing. Variables 1-4 were combined to create a composite variable with categories engagement in any anti-social behaviour and engagement in no anti-social behaviours (Cronbach alpha=0.95). Participants with missing data in ≥1 variables used to create the composite variable were coded as missing. |
| Number of siblings of participant in the household | MCS 5 (11-years) | Parental interview Participant siblings in the household | Continuous | 4-category variable (used in time-use-diary imputed analysis*)* Continuous variable categorised into 0,1,2-3, and 4-10 siblings.  5-category variable (used in remaining analyses)  Following observation of variable distribution, variable was categorised into 0,1,2-3,4-5 and 6-10 siblings. |
| Parental cigarette use (additionally used as proxy for parental e-cigarette use) | MCS 5 (11-years) | Parental interview  Parent current use of tobacco products | Yes/No/Not applicable | Binary composite variable  Not applicable responses were coded as missing. Using both main parental respondent and partner variable information (where relevant), a binary variable was created representing smoking habits across both parents in the household with categories 1+ smoker parents and non-smoker parents. For lone parents, their smoking status was captured. For 2 parent HHs if 1 parent was missing smoking status, the present parent’s smoking status was captured. If both parents were missing smoking status, the variable was classified as missing. If a lone parent was missing smoking status, the variable was classified as missing. |
| Parenting style | MCS 5 (11-years) | Parental interview  1. Whether parent has rules for how early or late participant may watch TV & films, use a computer, access the internet, or play electronic games 2. Whether parent has rules about the kinds of programmes or films participant can watch, electronic games they may play, or internet sites they can access | Yes/No/Don't know/Refused/Not applicable | Binary composite variable  Don’t know, refused, and not applicable responses were coded as missing. Variables 1 and 2 were collapsed to generate a binary composite variable with categories parent has rules for how early or late participant can watch TV & films, use a computer, access the internet, or play electronic games or rules about the kinds of programmes or films, electronic games, internet sites they can watch, or access and parent does not have rules for either (Cronbach alpha=0.61). |
| Urbanicity | MCS 5 (11-years) | Parental interview  1. ONS Rural Urban Classification (2005) England & Wales 2. Scottish Executive Urban Rural Classification 2005/6 (2005) Scotland 3. ONS Rural Urban Classification (2005) Northern Ireland | Variable 1: Urban > 10k– sparse/Urban > 10k-less sparse/Town and fringe–sparse/Village, hamlet, and isolated dwellings–sparse/Town and fringe-less sparse/Village, hamlet, and isolated dwellings-less sparse  Variables 2-3: Urban/Rural | Binary composite variable  Collapsed variable 1 into urban and rural and combined variables 1, 2, and 3 to create a binary variable with categories urban and rural. |
| Household income | MCS 5 (11-years) | Parental interview  Household income (OECD Income Equivalised Quintiles- UK Whole) | Bottom/Second/Third/ Fourth/Top quintile | 5-category variable  No alterations made to the existing variable. |
| Family structure | MCS 5 (11-years) | Parental interview  Family structure | Both natural parents/ Natural mother and step-parent/ Natural mother and other parent or carer/Natural mother and adoptive parent/Natural father and step-parent/Natural father and other parent or carer/Adoptive parents (x2)/Foster parents (x2)/ Grandparents (x2)/ Grandmother and other parent or carer/Other parents (x2)/Natural mother only/Natural father only/Adoptive mother only/Adoptive father only/Step-mother only/Other parent or carer only (foster/sibling/ relative)/Step-father only/ Grandfather only/Adoptive mother and step-parent | 3-category variable  Categories collapsed into natural parents, reconstituted and lone parent. |
| Highest parental occupation in the household | MCS 5 (11-years) | Parental interview  1. NS-SEC 5 category 2. Whether respondent is in work or not | Variable 1: Managerial and professional/Intermediate/Small employers and self-employed/Lower supervisory and technical/Semi routine and routine/Not applicable Variable 2: Respondent is in work or on leave/Respondent is not in work nor on leave/Not applicable | 6-category composite variable  Variable 1 and 2 not applicable responses were coded as missing. Variable 1 and variable 2 were combined to give NS-SEC 5 category variable with an additional category representing unemployed respondents for both the main parental respondent and partner respondent (where applicable). The highest occupation of both parents in the household (where relevant) was then used for analysis. For lone parents, their occupation was captured. For 2 parent HHs if 1 parent was missing occupation, the present parent’s occupation was captured. If both parents were missing occupation, the variable was classified as missing. If a lone parent was missing occupation, the variable was classified as missing. |
| Highest parental education in the household | MCS 5 (11-years) | Parental interview  NVQ equivalent of highest academic level across sweeps | NVQ level 5: higher degree/NVQ level 4: first degree, diplomas in higher education/NVQ level 3: A/AS/S levels/NVQ level 2: O level/GCSE grades A-C/ NVQ level 1: GCSE grades D-G/Other academic qualifications (incl. Overseas)/None of these/Not applicable | 7-category variable (used in complete case analyses)  Not applicable responses were coded as missing. The highest academic education level of both parents (where relevant) was used for analysis. For lone parents, their academic qualification was captured. For 2 parent HHs, if 1 parent was missing academic qualifications, the present parent’s academic qualification was captured. If both parents were missing academic qualification, the variable was classified as missing. If a lone parent was missing academic qualification, the variable was classified as missing  Binary variable (used in imputed analyses)  The above 7-category variable was dichotomised into high parental education (NVQ level 5, level 4, and level 3) and low parental education (NVQ level 2, level 1, Other academic qualifications (incl. Overseas), and None of these). |
| Area-level deprivation | MCS 5 (11-years) | Parental interview  1. IMD 2004 Overall Decile England  2. WIMD 2005 Overall Decile Wales  3. SIMD 2004 Overall Decile Scotland  4. IMD 2004 Overall Decile Northern Ireland  5. Country at interview | Variables 1-4: Most deprived/ 10-<20%/20-<30%/30-<40%/40-<50%/50-<60%/60-<70%/70-<80%/80-<90%/ Least deprived  Variable 5: England/Wales/Scotland/ Northern Ireland | 10-category composite variable  Variables 1-4 were combined, and variable 5 was used to create indicator variables representing country. |
| Baseline cigarette use (used for baseline adjustment) | MCS 6 (14-years) | Self-completion online questionnaire  How often participant smokes cigarettes | I have never smoked cigarettes/I have only ever tried smoking cigarettes once/I used to smoke sometimes but I never smoke a cigarette now/I sometimes smoke cigarettes now, but I don’t smoke as many as 1 a week/I usually smoke between 1 and 6 cigarettes a week/I usually smoke more than 6 cigarettes a week/Do not know/I do not wish to answer/No answer | 5-category variable  Do not know, I do not wish to answer, and no answer responses were coded as missing. Categories I usually smoke between 1 and 6 cigarettes a week and I usually smoke more than 6 cigarettes a week were combined to give I usually smoke 1 or more cigarettes a week. No alterations were made to the remaining categories. |
| Baseline  e-cigarette use (used for baseline adjustment) | MCS 6 (14-years) | Self-completion online questionnaire  How often participant smokes e-cigarettes | I've never used or tried electronic cigarettes (e-cigarettes)/I have used e-cigarettes but don't at all now/I now smoke e-cigarettes occasionally but not every day/I smoke e-cigarettes every day/Don't want to answer/Don't know/Not applicable | 4-category variable  Don’t want to answer, don't know and not applicable responses were coded as missing. No further alterations were made to the existing variable. |
| Previous social media use | MCS 5 (11-years) | Self-completion online questionnaire  How often participant visits a social networking website on the internet? | Most days/At least once a week/At least once a month/Less often than once a month/Never/No answer/Not applicable | 5-category variable (used in questionnaire imputed analyses)  No answer and not applicable responses were coded as missing. No further alterations were made to the existing variable. 4-category variable (used in time-use-diary imputed analyses)  No answer and not applicable responses were coded as missing. Categories at least once a month or less often than once a month were combined to give at least once a month or less than once a month. No alterations were made to the remaining categories. |

**Legend:** Main parental respondent refers to the main parent of the participant completing the interview and partner refers to the partner of the main respondent. Where variable structures differ between analyses (e.g., between complete case and imputed, or between questionnaire and time-use-diary) this was due to issues regarding imputation model convergence, thus variable structures were amended as required ensuring appropriate/meaningful categorisation. Abbreviations: BAS = British Ability Scales; BBM = Blackberry Messenger; CGT = Cambridge Gambling Task; Hrs = Hours; IMD = Indices of Multiple Deprivation; MCS = Millennium Cohort Study; NS-SEC = The National Statistics Socio-economic Classification; NVQ = National Vocational Qualification; SDQ = Strengths and Difficulties Questionnaire; OECD = Organisation for Economic Co-operation and Development, and ONS = Office for National Statistics.

# APPENDIX-C. Weights used in analyses

The Millennium Cohort Study (MCS) provides sample design weights to correct for cases having unequal probabilities of selection (resulting from the stratified cluster design) and non-response weights.^3^ The sample weights to be used depend on whether the analysis is confined to data relating to a single country (MCS sweep 7: GOVWT1), or whether the analysis covers all countries of the UK (MCS sweep 7: GOVWT2).

## Questionnaire analyses

Within the primary analysis models which include area-level deprivation as a confounder (and associated UK country level indicator variables), the single country sample design weight was used. Whilst in effect modification and interaction analysis models, where area-level deprivation (and associated country level indicator variables) were not included in the analysis models, the whole UK sample design weight was used.

## Time-use-diary analyses

For the time-use-diary analysis models, as the MCS does not provide a weight to make the time-use-diary sample representative of the entire sample, we created time-use-diary specific weights, which additionally helped to account for participants excluded due to having ≥5 ‘no activity’ slots recorded (thus deemed as having unreliable diary entries) and participants who did not complete the time-use-diary at all.

To create the weights, a logistic regression approach to weighting was used.^4^ We identified the characteristics which may have influenced a participants ability to complete the time-use-diary on both the weekday and weekend day as well as complete it reliably. This was achieved by entering all identified characteristics as independent variables into a logistic regression model with the binary dependent variable 1=completed the diary on both days (and reliably thus did not have ≥5 slots recorded as ‘no activity’) and 0=did not complete the diary on both days reliably or did not complete at all. Following this, we removed characteristics not identified as significant predictors of our dependent binary variable in a sequential manner, until left with a model with only significant predictors (listed below):

- Sex
- Mental health (assessed via Strengths and Difficulties Questionnaire)
- Cognitive ability (assessed via British Ability Scale II Verbal Similarities)
- Risk-taking (assessed via Cambridge Gambling Task)
- Anti-social behaviour
- Parental smoking
- Household income (assessed via Organisation for Economic Co-operation and Development Income Equivalised Quintiles)
- England country indicator variable

In line with the predictors used by the MCS when creating non-response weights, ethnicity, age, highest parental education in the household and family structure were additionally added to the model.^3,5^

In the final model, the predicted probabilities were obtained, and the inverse of the predicted probabilities calculated to obtain the time-use-diary weight. The time-use-diary weight was then multiplied by both the single country analysis weight (time-use-diary weight*GOVWT1) and whole UK analysis weight (time-use-diary weight*GOVWT2) to obtain the final weights to be used in the respective analyses (see above).

# APPENDIX-D. Differential effect of social media on cigarette and e-cigarette use: effect modification and interaction analyses

## Effect modification: association between social media use and cigarette and e-cigarette use, according to strata of parental education

We estimated risk differences (RDs) representing the absolute difference in cigarette/e-cigarette use by social media use, within the low and high parental education groups. Measures of effect modification on the additive scale represent the size of the absolute difference between RDs for cigarette/e-cigarette use by social media use between high and low parental education groups (baseline: high parental education). A measure greater (or less) than zero indicates the presence of a positive (or negative) additive interaction.

## Interaction: risk of cigarette and e-cigarette use according to ‘combinations’ of social media use and parental education

We estimated RDs for cigarette/e-cigarette use according to the combination of social media use and parental education (baseline: high parental education and no social media use (stratum with the lowest risk of cigarette/e-cigarette use)).^6^ The measure of interaction represents the size of the difference between the RD in participants with (e.g.) low parental education and 1-<30 minutes social media use, compared with the RD for participants with low parental education and no social media use, plus the RD for those with high parental education and 1-<30 minutes social media use.

## Additional/sensitivity analyses

Interaction should be examined alongside effect modification, when the effect modifier is a potential cause of the outcome, as is likely for parental education and participants social media use.^7^ A key assumption in analysis of effect modification is that the exposure (in this case, social media use) is not a cause of the effect modifier (parental education). We theorise that participant social media use does not influence parental education.^8^

In line with epidemiological recommendations, we report results on both additive and multiplicative scales.^7^

# APPENDIX-E. Variables included within imputation models

To account for the non-normal distribution of continuous variables standard regression was not used, instead, as recommended, predictive mean matching was used.^9^ Predictive mean matching was also used for nominal categorical variables where multinomial and ordered logit did not facilitate model convergence, and for binary variables where logistic did not facilitate model convergence.^9^

Table-G1 details the variables included within each of the imputation models.

Table-G1: Variables included within the imputation models

| **Variable** | **Questionnaire imputed sample (n=8,987)** | | **Questionnaire imputed sample (n=8,954)** | | **Time-use-diary imputed sample (n=2,520)** | | **Time-use-diary imputed sample (n=2,520)** | |
| --- | --- | --- | --- | --- | --- | --- | --- | --- |
|  | **Primary analysis** | | **Effect modification and interaction analysis** | | **Primary analysis** | | **Effect modification and interaction analysis** | |
|  | **Regression model** | **n (%)**  **imputed** | **Regression model** | **n (%)**  **imputed** | **Regression model** | **n (%)**  **imputed** | **Regression model** | **n (%)**  **imputed** |
| Time spent on social media on a normal weekday (time-use diary) | - | - | - | - | Complete | 0 (0) | Complete | 0 (0) |
| Average time spent on social media across a normal weekday and weekend day (time-use diary) | - | - | - | - | Complete | 0 (0) | Complete | 0 (0) |
| Time spent on social media on a normal weekday (questionnaire) | Ordered logit | 105 (1.2) | Ordered logit | 105 (1.2) | - | - | - | - |
| Cigarette use^a^ | Multinomial logit | 216 (2.4) | Multinomial logit | 215 (2.4) | Multinomial logit | 27 (1.1) | Multinomial logit | 27 (1.1) |
| E-cigarette use^a^ | Multinomial logit | 220 (2.4) | Multinomial logit | 219 (2.4) | Multinomial logit | 31 (1.2) | Multinomial logit | 31 (1.2) |
| Maternal age at participant birth | Predictive mean matching | 15 (0.2) | Predictive mean matching | 13 (0.1) | Predictive mean matching | 5 (0.2) | Predictive mean matching | 5 (0.2) |
| Mental health | Predictive mean matching | 336 (3.7) | Predictive mean matching | 322 (0.0) | Complete | 0 (0) | Complete | 0 (0) |
| Previous cigarette use (also used as proxy for previous e-cigarette use) | Logistic | 261 (2.9) | Logistic | 256 (2.9) | Logistic | 13 (0.5) | Predictive mean matching | 13 (0.5) |
| Previous alcohol use | Logistic | 346 (3.9) | Logistic | 340 (3.8) | Logistic | 33 (1.3) | Predictive mean matching | 33 (1.3) |
| In-person activities | Predictive mean matching | 48 (0.5) | Predictive mean matching | 34 (0.4) | Predictive mean matching | 4 (0.2) | Predictive mean matching | 4 (0.2) |
| Cognitive ability | Predictive mean matching | 121 (1.3) | Predictive mean matching | 120 (1.3) | Complete | 0 (0) | Complete | 0 (0) |
| Risk-taking | Predictive mean matching | 420 (4.7) | Predictive mean matching | 418 (4.7) | Complete | 0 (0) | Complete | 0 (0) |
| Anti-social behaviour | Logistic | 314 (3.5) | Logistic | 312 (3.5) | Complete | 0 (0) | Complete | 0 (0) |
| Parental cigarette use (also used as a proxy for parental e-cigarette use) | Logistic | 28 (0.3) | Logistic | 14 (0.2) | Complete | 0 (0) | Complete | 0 (0) |
| Parenting style | Logistic | 46 (0.5) | Logistic | 32 (0.4) | Logistic | 3 (0.1) | Predictive mean matching | 3 (0.1) |
| Urbanicity | Logistic | 1,476 (16.4) | Logistic | 1,470 (16.4) | Logistic | 320 (12.7) | Logistic | 320 (12.7) |
| Highest parental occupation in the household | Ordered logit | 121 (1.3) | Ordered logit | 107 (1.2) | Ordered logit | 24 (1.0) | Ordered logit | 24 (1.0) |
| Highest parental education in the household | Logistic | 33 (0.4) | Complete: interaction by() | 0 (0)^b^ | Complete | 0 (0) | Complete: interaction by() | 0 (0) |
| Area-level deprivation | Ordered logit | 5 (0.1) | Ordered logit | 5 (0.1) | Ordered logit | 1 (0.0) | Ordered logit | 1 (0.0) |
| Baseline cigarette use | Predictive mean matching | 256 (2.8) | Predictive mean matching | 254 (2.8) | Predictive mean matching | 21 (0.8) | Predictive mean matching | 21 (0.8) |
| Baseline e-cigarette use | Predictive mean matching | 247 (2.7) | Predictive mean matching | 245 (2.7) | Predictive mean matching | 20 (0.7) | Predictive mean matching | 20 (0.7) |
| Previous social media use | Ordered logit | 202 (2.2) | Ordered logit | 201 (2.2) | Ordered logit | 18 (0.7) | Ordered logit | 18 (0.7) |
| Sex | Complete | 0 (0) | Complete | 0 (0) | Complete | 0 (0) | Complete | 0 (0) |
| Age | Complete | 0 (0) | Complete | 0 (0) | Complete | 0 (0) | Complete | 0 (0) |
| Ethnic group | Complete | 0 (0) | Complete | 0 (0) | Complete | 0 (0) | Complete | 0 (0) |
| Number of siblings in the household | Complete | 0 (0) | Complete | 0 (0) | Complete | 0 (0) | Complete | 0 (0) |
| Household income | Complete | 0 (0) | Complete | 0 (0) | Complete | 0 (0) | Complete | 0 (0) |
| Family structure | Complete | 0 (0) | Complete | 0 (0) | Complete | 0 (0) | Complete | 0 (0) |
| UK country indicator variable | Complete | 0 (0) | Complete | 0 (0) | Complete | 0 (0) | Complete | 0 (0) |
| GOVWT1 (MCS single country weight) | Complete | 0 (0) | - | - | - | - | - | - |
| GOVWT2 (MCS whole UK country weight) | - | - | Complete | 0 (0) | - | - | - | - |
| Time-use diary weight*GOVWT1 (MCS single country weight) | - | - | - | - | Complete | 0 (0) | - | - |
| Time-use diary weight*GOVWT2 (MCS whole UK country weight) | - | - | - | - | - | - | Complete | 0 (0) |

**Legend:** ^a^ Three category variables imputed, and binary variables and composite variable representing dual use created post imputation. ^b^ To facilitate inclusion of interaction between time spent on social media and highest parental education in the imputation model for effect modification and interaction analyses, n = 33 with missing data on highest parental education excluded prior to imputation. Abbreviations: HH = Household.

# APPENDIX-F. Characteristics of imputed and complete case samples

Table-F1. Characteristics of imputed and complete case samples

|  | **Questionnaire complete case sample (n = 6,234)** | | **Questionnaire imputed sample  (n = 8,987)** | | **Time-use-diary complete case sample (n = 2,109)** | | **Time-use-diary imputed sample (n = 2,520)** | |
| --- | --- | --- | --- | --- | --- | --- | --- | --- |
| **Characteristic** | **n/mean & SD** | **Weighted % (95% CI)/mean & SD^a^** | **n/mean & SD** | **Weighted % (95% CI)/mean & SD^a^** | **n/mean & SD** | **Weighted % (95% CI)/mean & SD^b^** | **n/mean & SD** | **Weighted % (95% CI)/mean & SD^b^** |
| **Time spent on social media on a normal weekday (questionnaire)** | | | | | | | | |
| No social media use | 456 | 7.5 (6.7 to 8.4) | 734 | 8.4 (7.6 to 9.1) | - | - | - | - |
| 1 - <30 mins social media use | 798 | 13.0 (12.0 to 13.9) | 1,147 | 12.8 (12.0 to 13.6) | - | - | - | - |
| 30 mins - <1 hr social media use | 939 | 15.2 (14.2 to 16.2) | 1,330 | 14.9 (13.9 to 15.8) | - | - | - | - |
| 1 - <2 hrs social media use | 1,087 | 17.4 (16.3 to 18.6) | 1,562 | 17.3 (16.4 to 18.3) | - | - | - | - |
| ≥ 2 hrs social media use | 2,954 | 46.9 (45.4 to 48.5) | 4,214 | 46.6 (45.4 to 47.8) | - | - | - | - |
| **Time spent on social media on a normal weekday (time-use-diary)** | | | | | | | | |
| No social media use | - | - | - | - | 1,288 | 63.0 (60.6 to 65.3) | 1,548 | 63.8 (61.7 to 65.9) |
| 1 - <30 mins social media use | - | - | - | - | 197 | 8.7 (7.5 to 10.0) | 230 | 8.3 (7.2 to 9.5) |
| 30 mins - <1 hr social media use | - | - | - | - | 230 | 9.9 (8.6 to 11.4) | 269 | 9.9 (8.6 to 11.1) |
| 1 - <2 hrs social media use | - | - | - | - | 228 | 10.3 (8.9 to 11.8) | 277 | 10.2 (8.9 to 11.5) |
| ≥ 2 hrs social media use | - | - | - | - | 166 | 8.2 (6.7 to 9.9) | 196 | 7.8 (6.5 to 9.1) |
| **Average time spent on social media across a normal weekday and weekend day (time-use-diary)** | | | | | | | | |
| No social media use | - | - | - | - | 978 | 48.2 (45.8 to 50.6) | 1,178 | 49.0 (46.8 to 51.2) |
| 1 - <30 mins social media use | - | - | - | - | 416 | 18.8 (16.9 to 20.9) | 497 | 18.8 (17.1 to 20.6) |
| 30 mins - <1 hr social media use | - | - | - | - | 331 | 14.2 (12.7 to 15.9) | 390 | 14.3 (12.7 to 15.8) |
| 1 - <2 hrs social media use | - | - | - | - | 231 | 11.2 (9.7 to 13.1) | 276 | 10.7 (9.2 to 12.2) |
| ≥ 2 hrs social media use | - | - | - | - | 153 | 7.5 (6.2 to 9.1) | 179 | 7.2 (5.9 to 8.5) |
| **Cigarette use** | | | | | | | | |
| Never used cigarettes or tried once | 4,504 | 70.8 (69.3 to 72.2) | 6,603 | 71.1 (69.7 to 72.5) | 1,634 | 74.7 (72.2 to 77.1) | 1,960 | 74.6 (72.4 to 76.9) |
| Former cigarette use | 421 | 7.0 (6.3 to 7.7) | 604 | 7.0 (6.4 to 7.6) | 114 | 6.2 (5.0 to 7.7) | 139 | 7.0 (5.5 to 8.5) |
| Current cigarette use | 1,309 | 22.2 (21.0 to 23.6) | 1,780 | 21.9 (20.7 to 23.1) | 361 | 19.0 (17.0 to 21.2) | 421 | 18.3 (16.4 to 20.2) |
| **E-cigarette use** | | | | | | | | |
| Never used e-cigarettes or tried once | 4,845 | 77.1 (75.7 to 78.4) | 6,929 | 76.2 (74.9 to 77.5) | 1,742 | 79.6 (77.1 to 81.9) | 2,063 | 79.0 (76.8 to 81.2) |
| Former e-cigarette use | 688 | 11.2 (10.3 to 12.1) | 1,030 | 11.7 (10.8 to 12.7) | 181 | 9.7 (8.3 to 11.4) | 219 | 10.0 (8.5 to 11.5) |
| Current e-cigarette use | 701 | 11.7 (10.8 to 12.7) | 1,028 | 12.0 (11.1 to 13.0) | 186 | 10.7 (9.0 to 12.7) | 238 | 11.0 (9.3 to 12.7) |
| **Current dual use of cigarettes and e-cigarettes** | | | | | | | | |
| Never used both cigarettes or e-cigarettes or tried once | 4,137 | 65.0 (63.4 to 66.6) | 6,000 | 64.8 (63.4 to 66.2) | 1,536 | 69.3 (66.5 to 72.0) | 1,831 | 68.9 (66.4 to 71.4) |
| Current or former cigarette or e-cigarette use | 1,618 | 26.9 (25.44 to 28.3) | 2,315 | 27.0 (25.7 to 28.2) | 447 | 23.4 (21.1 to 25.9) | 535 | 23.9 (21.6 to 26.2) |
| Current dual use | 479 | 8.1 (7.4 to 8.9) | 672 | 8.2 (7.5 to 9.0) | 126 | 7.3 (6.0 to 8.8) | 153 | 7.2 (6.0 to 8.4) |
| **Confounding variables** |  |  |  |  |  |  |  |  |
| **Maternal age at participant birth** | | | | | | | | |
| 11 to 19 years | 310 | 4.9 (4.3 to 5.7) | - | - | 69 | 4.2 (3.1 to 5.6) | - | - |
| 20 to 29 years | 2,569 | 40.0 (37.9 to 42.2) | - | - | 821 | 40.7 (38.0 to 43.5) | - | - |
| 30 to 39 years | 3,179 | 52.2 (50.0 to 54.4) | - | - | 1,158 | 52.4 (49.6 to 55.2) | - | - |
| 40+ years | 176 | 2.8 (2.4 to 3.2) | - | - | 61 | 2.7 (2.1 to 3.4) | - | - |
| Mean (SD) | - | - | 29.3 (5.69) | 29.4 (5.67) | - | - | 30.0 (5.29) | 29.7 (5.46) |
| **Sex** | **Sex** | **Sex** | **Sex** | **Sex** | **Sex** | **Sex** | **Sex** | **Sex** |
| Male | Male | Male | Male | Male | Male | Male | Male | Male |
| Female | Female | Female | Female | Female | Female | Female | Female | Female |
| **Age** |  |  |  |  |  |  |  |  |
| 13-years | 1,586 | 25.3 (24.0 to 26.7) | 2,282 | 25.1 (23.9 to 26.2) | 552 | 25.7 (23.4 to 28.2) | 658 | 26.2 (23.9 to 28.5) |
| 14-years | 4,584 | 73.4 (72.0 to 74.7) | 6,610 | 73.7 (72.5 to 74.8) | 1,541 | 73.6 (71.1 to 75.9) | 1,843 | 73.0 (70.7 to 75.4) |
| 15-years | 64 | 1.3 (0.9 to 1.8) | 95 | 1.3 (0.9 to 1.6) | 16 | 0.7 (0.4 to 1.2) | 19 | 0.8 (0.4 to 1.2) |
| **Ethnic group** |  |  |  |  |  |  |  |  |
| White | 5,467 | 93.2 (91.7 to 94.5) | 7,346 | 91.0 (89.3 to 92.6) | 1,915 | 93.3 (91.1 to 94.9) | 2,228 | 92.1 (90.2 to 94.0) |
| Mixed | 160 | 2.2 (1.7 to 2.8) | 1,641 | 9.0 (7.4 to 10.7) | 194 | 6.7 (5.1 to 8.9) | 292 | 7.9 (6.0 to 9.8) |
| Indian | 141 | 1.1 (0.8 to 1.5) |  |  |  |  |  |  |
| Pakistani & Bangladeshi | 214 | 1.4 (1.0 to 2.1) |  |  |  |  |  |  |
| Black/Black British | 171 | 1.5 (1.0 to 2.1) |  |  |  |  |  |  |
| Other ethnic group (including Chinese, Other) | 81 | 0.7 (0.5 to 1.0) |  |  |  |  |  |  |
| **Mental health (SDQ total difficulties)** | | | | | | | | |
| Mean (SD) | 7.05 (5.44) | 7.06 (5.58) | 7.42 (5.67) | 7.46 (5.89) | 6.11 (4.86) | 7.01 (5.62) | 6.23 (4.93) | 7.22 (5.81) |
| **Previous cigarette use (also used as proxy for previous e-cigarette use)** | | | | | | | | |
| No | 6,117 | 98.1 (97.7 to 98.5) | 8,800 | 97.8 (97.4 to 98.3) | 2,087 | 98.4 (97.4 to 99.0) | 2,495 | 98.4 (97.7 to 99.2) |
| Yes | 117 | 1.9 (1.5 to 2.3) | 187 | 2.2 (1.7 to 2.6) | 22 | 1.6 (1.0 to 2.6) | 26 | 1.6 (0.8 to 2.3) |
| **Previous alcohol use** |  |  |  |  |  |  |  |  |
| No | 5,521 | 87.9 (86.9 to 88.8) | 8,001 | 88.1 (87.2 to 89.0) | 1,901 | 89.2 (87.4 to 90.7) | 2,282 | 89.4 (87.8 to 91.0) |
| Yes | 713 | 12.1 (11.2 to 13.1) | 986 | 11.9 (11.0 to 12.8) | 208 | 10.8 (9.3 to 12.6) | 238 | 10.6 (9.0 to 12.2) |
| **Average days/week of in-person activities** | | | | | | | | |
| Mean (SD) | 3.04 (1.53) | 3.12 (1.51) | 2.93 (1.56) | 3.04 (1.54) | 3.11 (1.52) | 3.12 (1.50) | 3.07 (1.53) | 3.07 (1.53) |
| **Cognitive ability** |  |  |  |  |  |  |  |  |
| Mean (SD) | 60.3 (9.39) | 60.2 (9.39) | 59.4 (10.0) | 59.6 (9.87) | 61.5 (8.75) | 60.4 (8.82) | 61.3 (8.84) | 60.1 (9.02) |
| **Risk-taking** |  |  |  |  |  |  |  |  |
| Mean (SD) | 0.52 (0.17) | 0.52 (0.17) | 0.52 (0.17) | 0.52 (0.17) | 0.50 (0.17) | 0.52 (0.17) | 0.50 (0.17) | 0.52 (0.17) |
| **Anti-social behaviour** |  |  |  |  |  |  |  |  |
| No | 4,997 | 80.1 (78.8 to 81.3) | 7,158 | 79.7 (78.6 to 80.8) | 1,784 | 80.7 (78.3 to 82.8) | 2,131 | 80.4 (78.1 to 82.6) |
| Yes | 1,237 | 19.9 (18.7 to 21.2) | 1,829 | 20.3 (19.2 to 21.4) | 325 | 19.3 (17.2 to 21.7) | 389 | 19.6 (17.4 to 21.9) |
| **Number of siblings of participant in the household** | | | | | | | | |
| 0 | 670 | 11.2 (10.2 to 12.1) | 984 | 11.7 (10.9 to 12.5) | 205 | 10.0 (8.6 to 11.5) | 266 | 11.5 (9.9 to 13.1) |
| 1 | 2,914 | 47.8 (46.4 to 49.3) | 4,018 | 46.9 (45.5 to 48.3) | 1,054 | 47.3 (44.8 to 49.7) | 1,237 | 46.3 (44.0 to 48.5) |
| 2-3 | 2,401 | 37.1 (35.7 to 38.6) | 3,490 | 37.0 (35.8 to 38.3) | 793 | 39.0 (36.6 to 41.5) | 929 | 38.1 (35.8 to 40.5) |
| 4-5 | 220 | 3.5 (2.9 to 4.1) | 435 | 3.9 (3.3 to 4.5) | 49 | 3.1 (2.1 to 4.1) | 88 | 4.1 (2.9 to 5.3) |
| 6-10 | 29 | 0.4 (0.3 to 0.7) | 60 | 0.5 (0.3 to 0.6) | 8 | 0.6 (0.3 to 1.6) |  |  |
| **Parental cigarette use (also used as proxy for parental e-cigarette use)** | | | | | | | | |
| Non-smoker parents | 4,495 | 73.0 (71.5 to 74.5) | 6,416 | 72.1 (70.7 to 73.5) | 1,655 | 73.3 (70.7 to 75.7) | 1,949 | 72.1 (69.7 to 74.5) |
| 1+ smoker parents | 1,739 | 27.0 (25.5 to 28.5) | 2,571 | 27.9 (26.5 to 29.3) | 454 | 26.7 (24.3 to 29.3) | 571 | 27.9 (25.5 to 30.3) |
| **Parenting style** |  |  |  |  |  |  |  |  |
| Parent has rules | 6,081 | 97.3 (96.6 to 97.9) | 8,726 | 97.1 (96.6 to 97.6) | 2,056 | 97.6 (96.7 to 98.3) | 2,453 | 97.5 (96.8 to 98.2) |
| Parent does not have rules | 153 | 2.7 (2.1 to 3.4) | 261 | 2.9 (2.4 to 3.4) | 53 | 2.4 (1.7 to 3.3) | 67 | 2.5 (1.8 to 3.2) |
| **Urbanicity** |  |  |  |  |  |  |  |  |
| Urban | 4,579 | 69.3 (65.4 to 73.0) | 6,818 | 71.0 (67.8 to 74.2) | 1,496 | 69.2 (64.6 to 73.4) | 1,825 | 70.7 (66.7 to 74.7) |
| Rural | 1,655 | 30.7 (27.0 to 34.6) | 2,169 | 29.0 (25.8 to 32.2) | 613 | 30.8 (26.6 to 35.4) | 695 | 29.3 (25.3 to 33.3) |
| **Household income** |  |  |  |  |  |  |  |  |
| Top quintile | 1,623 | 29.2 (26.9 to 31.5) | 2,075 | 27.1 (25.0 to 29.1) | 677 | 29.1 (26.6 to 31.7) | 776 | 28.0 (25.6 to 30.4) |
| Fourth quintile | 1,551 | 26.1 (24.7 to 27.7) | 2,062 | 25.0 (23.6 to 26.4) | 599 | 26.5 (24.1 to 29.0) | 658 | 25.6 (23.4 to 27.8) |
| Third quintile | 1,372 | 21.0 (19.8 to 22.3) | 1,883 | 20.6 (19.4 to 21.8) | 446 | 21.6 (19.8 to 23.6) | 533 | 21.6 (19.7 to 23.5) |
| Second quintile | 998 | 14.6 (13.5 to 15.9) | 1,514 | 15.4 (14.4 to 16.5) | 260 | 14.6 (12.6 to 16.9) | 312 | 14.5 (12.6 to 16.4) |
| Bottom quintile | 690 | 9.1 (8.0 to 10.3) | 1,453 | 11.9 (10.5 to 13.3) | 127 | 8.1 (6.4 to 10.2) | 214 | 10.4 (8.4 to 12.3) |
| **Family structure** |  |  |  |  |  |  |  |  |
| Natural parents | 4,502 | 70.6 (68.8 to 72.3) | 6,397 | 69.2 (67.6 to 70.8) | 1,668 | 71.1 (68.3 to 73.7) | 1,969 | 69.3 (66.8 to 71.9) |
| Reconstituted | 561 | 9.6 (8.6 to 10.7) | 810 | 10.0 (9.1 to 10.9) | 148 | 8.9 (7.3 to 10.8) | 178 | 9.1 (7.4 to 10.8) |
| Lone parent | 1,171 | 19.8 (18.5 to 21.1) | 1,780 | 20.9 (19.7 to 22.1) | 293 | 20.0 (17.8 to 22.5) | 373 | 21.6 (19.2 to 23.9) |
| **Highest parental occupation in the household** | | | | | | | | |
| Managerial and professional | 3,157 | 52.0 (49.8 to 54.2) | 4,166 | 49.0 (46.9 to 51.1) | 1,188 | 50.7 (47.4 to 54.0) | 1,382 | 49.3 (46.3 to 52.4) |
| Intermediate | 753 | 12.4 (11.4 to 13.4) | 1,069 | 12.6 (11.7 to 13.5) | 275 | 13.7 (12.0 to 15.6) | 329 | 13.8 (12.1 to 15.5) |
| Small employers and self-employed | 566 | 9.0 (8.2 to 9.9) | 873 | 9.2 (8.4 to 10.0) | 188 | 9.5 (8.0 to 11.2) | 231 | 9.3 (7.8 to 10.8) |
| Lower supervisory and technical | 243 | 3.7 (3.1 to 4.3) | 370 | 3.9 (3.4 to 4.4) | 62 | 3.5 (2.5 to 4.9) | 79 | 3.5 (2.5 to 4.5) |
| Semi routine and routine | 724 | 11.6 (10.5 to 12.8) | 1,129 | 12.0 (10.9 to 13.0) | 221 | 11.9 (10.1 to 13.9) | 262 | 11.5 (9.8 to 13.3) |
| Unemployed | 791 | 11.4 (10.3 to 12.5) | 1,380 | 13.4 (12.2 to 14.5) | 175 | 10.7 (9.0 to 12.8) | 237 | 12.6 (10.6 to 14.5) |
| **Highest parental education in the household** | | | | | | | | |
| NVQ level 5- higher degree | 1,031 | 16.7 (15.3 to 18.3) | 5,168 | 59.1 (56.9 to 61.4) | 400 | 15.6 (13.7 to 17.7) | 1,712 | 61.6 (58.5 to 64.7) |
| NVQ level 4- first degree, diplomas in higher education | 2,283 | 36.9 (35.2 to 38.6) |  |  | 865 | 38.1 (35.4 to 40.8) |  |  |
| NVQ level 3- A/AS/S levels | 546 | 9.0 (8.2 to 9.9) |  |  | 211 | 10.3 (8.8 to 11.9) |  |  |
| NVQ level 2- O level/GCSE grades A-C | 1,580 | 25.9 (24.1 to 27.8) | 3,819 | 40.9 (38.6 to 43.1) | 460 | 25.6 (23.0 to 28.3) | 808 | 38.4 (35.3 to 41.5) |
| NVQ level 1- GCSE grades D-G | 337 | 5.2 (4.5 to 6.0) |  |  | 85 | 4.9 (3.9 to 6.1) |  |  |
| Other academic qualifications (incl. Overseas) | 119 | 1.5 (1.1 to 1.9) |  |  | 30 | 1.6 (1.1 to 2.4) |  |  |
| None of the above | 338 | 4.9 (4.2 to 5.7) |  |  | 58 | 4.0 (2.8 to 5.5) |  |  |
| **Area-level deprivation** |  |  |  |  |  |  |  |  |
| Least deprived | 841 | 15.9 (13.3 to 19.0) | 1,037 | 14.2 (11.8 to 16.7) | 332 | 16.3 (13.3 to 19.7) | 353 | 14.5 (11.7 to 17.3) |
| 80 - <90% | 749 | 14.2 (12.5 to 16.1) | 929 | 13.0 (11.3 to 14.6) | 282 | 14.9 (12.6 to 17.5) | 306 | 13.4 (11.2 to 15.5) |
| 70 - <80% | 623 | 11.2 (9.7 to 12.9) | 795 | 10.2 (8.8 to 11.6) | 231 | 11.2 (9.3 to 13.4) | 270 | 10.9 (8.9 to 12.8) |
| 60 - <70% | 615 | 10.6 (9.4 to 12.0) | 809 | 10.3 (9.0 to 11.6) | 231 | 11.0 (9.3 to 13.0) | 275 | 11.4 (9.7 to 13.2) |
| 50 - <60% | 636 | 11.0 (9.4 to 12.8) | 866 | 10.8 (9.3 to 12.3) | 205 | 10.4 (8.4 to 12.7) | 247 | 10.3 (8.4 to 12.2) |
| 40 - <50% | 617 | 9.2 (7.9 to 10.6) | 833 | 9.2 (8.0 to 10.4) | 225 | 10.4 (8.4 to 12.8) | 260 | 10.1 (8.2 to 12.1) |
| 30 - <40% | 580 | 8.1 (7.0 to 9.3) | 822 | 8.3 (7.3 to 9.3) | 193 | 8.5 (7.0 to 10.4) | 227 | 8.9 (7.2 to 10.6) |
| 20 - <30% | 572 | 7.9 (6.8 to 9.2) | 901 | 8.6 (7.4 to 9.7) | 169 | 7.8 (6.3 to 9.5) | 213 | 8.5 (6.9 to 10.1) |
| 10 - <20% | 613 | 7.4 (6.3 to 8.6) | 999 | 8.4 (7.3 to 9.4) | 154 | 6.1 (4.9 to 7.6) | 201 | 6.7 (5.4 to 7.9) |
| Most deprived | 388 | 4.5 (3.6 to 5.6) | 996 | 7.0 (5.7 to 8.4) | 87 | 3.4 (2.5 to 4.6) | 167 | 5.3 (3.8 to 6.8) |

**Legend:** ^a^ Weighted to account attrition and sample design at the MCS 7 (17-year survey). ^b^ Weighted to account for time-use-diary non-response at MCS 6 (14-year survey) and for attrition and sample design at the MCS 7 (17-year survey). Values may not add up due to rounding. Abbreviations: CI = Confidence interval; GCSE = General Certificate in Secondary Education; n = Number of participants; NVQ = National Vocational Qualifications; SD = Standard deviation, and SDQ = Strengths and Difficulties Questionnaire.

# APPENDIX-G. Effect of social media use on cigarette, e-cigarette and dual use

Table-G1 Time spent on social media on a normal weekday on risk of (A) cigarette, (B) e-cigarette, and (C) dual use within the questionnaire imputed sample, stratified by sex

|  |  | **Total population (n = 8,987)** | | | **Males (n = 4,383)** | | | **Females (n = 4,604)** | | |
| --- | --- | --- | --- | --- | --- | --- | --- | --- | --- | --- |
|  |  | **Weighted prevalence % (observed n with outcome)** | **OR (95% CI)** | **AOR (95% CI)^a^** | **Weighted prevalence % (observed n with outcome)** | **OR (95% CI)** | **AOR (95% CI)^a^** | **Weighted prevalence % (observed n with outcome)** | **OR (95% CI)** | **AOR (95% CI)^a^** |
| **Time spent on social media on a normal weekday (questionnaire)** | | | | | | | | | | |
| **A. Current or former cigarette use (ref: never used a cigarette or tried once)** | | | | | | | | | | |
| No social media use | | 14.9 (94) | 0.83 (0.56 to 1.22) | 0.82 (0.57 to 1.18) | 15.7 (66) | 0.85 (0.52 to 1.38) | 0.86 (0.54 to 1.36) | 13.2 (28) | 0.77 (0.43 to 1.36) | 0.81 (0.46 to 1.42) |
| 1 - <30 mins | | 17.4 (175) | 1.00 | 1.00 | 17.9 (119) | 1.00 | 1.00 | 16.5 (56) | 1.00 | 1.00 |
| 30 mins - <1 hr | | 22.9 (275) | 1.41 (1.05 to 1.89) | 1.48 (1.11 to 1.97) | 25.2 (184) | 1.55 (1.06 to 2.25) | 1.56 (1.10 to 2.23) | 19.3 (91) | 1.21 (0.77 to 1.89) | 1.39 (0.89 to 2.17) |
| 1 - <2 hrs | | 26.9 (395) | 1.74 (1.35 to 2.26) | 1.78 (1.38 to 2.29) | 28.2 (219) | 1.80 (1.29 to 2.53) | 1.75 (1.27 to 2.40) | 25.5 (176) | 1.73 (1.15 to 2.58) | 1.84 (1.24 to 2.72) |
| ≥ 2 hrs | | 37.2 (1,445) | 2.80 (2.23 to 3.52) | 2.76 (2.19 to 3.48) | 39.0 (545) | 2.93 (2.16 3.99) | 2.76 (2.04 to 3.75) | 36.1 (900) | 2.86 (1.96 to 4.16) | 2.84 (1.97 to 4.09) |
| **B. Current or former e-cigarette use (ref: never used e-cigarette or tried once)** | | | | | | | | | | |
| No social media use | | 13.9 (88) | 1.01 (0.66 to 1.53) | 0.94 (0.63 to 1.39) | 16.4 (69) | 0.99 (0.61 to 1.60) | 0.95 (0.61 to 1.49) | 7.8 (19) | 0.92 (0.42 to 1.99) | 0.96 (0.45 to 2.08) |
| 1 - <30 mins | | 13.8 (157) | 1.00 | 1.00 | 16.5 (125) | 1.00 | 1.00 | 8.4 (32) | 1.00 | 1.00 |
| 30 mins - <1 hr | | 20.9 (262) | 1.65 (1.24 to 2.20) | 1.79 (1.34 to 2.39) | 25.8 (200) | 1.76 (1.25 to 2.48) | 1.76 (1.26 to 2.47) | 13.0 (61) | 1.62 (0.99 to 2.64) | 1.89 (1.14 to 3.16) |
| 1 - <2 hrs | | 22.4 (351) | 1.80 (1.42 to 2.29) | 2.06 (1.61 to 2.64) | 29.2 (245) | 2.09 (1.57 to 2.78) | 2.07 (1.55 to 2.75) | 14.9 (106) | 1.90 (1.21 to 2.99) | 2.02 (1.26 to 3.22) |
| ≥ 2 hrs | | 29.8 (1,200) | 2.65 (2.14 to 3.29) | 3.24 (2.59 to 4.05) | 41.0 (585) | 3.52 (2.71 to 4.57) | 3.23 (2.47 to 4.21) | 23.3 (615) | 3.31 (2.21 to 4.95) | 3.25 (2.12 to 4.98) |
|  |  | **Weighted prevalence % (observed n with outcome)** | **RRR (95% CI)** | **ARRR (95% CI)^a^** | **Weighted prevalence % (observed n with outcome)** | **RRR (95% CI)** | **ARRR (95% CI)^a^** | **Weighted prevalence % (observed n with outcome)** | **RRR (95% CI)** | **ARRR (95% CI)^a^** |
| **C. Current or former cigarette or e-cigarette use (ref: never used cigarette or e-cigarette or tried once)** | | | | | | | | | | |
| No social media use | | 15.0 (95) | 0.82 (0.56 to 1.19) | 0.78 (0.55 to 1.12) | 15.9 (64) | 0.80 (0.49 to 1.30) | 0.76 (0.48 to 1.20) | 12.3 (31) | 0.82 (0.46 to 1.49) | 0.83 (0.46 to 1.48) |
| 1 - <30 mins | | 17.7 (186) | 1.00 | 1.00 | 19.1 (133) | 1.00 | 1.00 | 15.2 (52) | 1.00 | 1.00 |
| 30 mins - <1 hr | | 23.8 (302) | 1.49 (1.13 to 1.97) | 1.58 (1.21 to 2.07) | 27.5 (211) | 1.67 (1.18 to 2.37) | 1.70 (1.22 to 2.36) | 18.1 (90) | 1.25 (0.79 to 1.99) | 1.41 (0.90 to 2.24) |
| 1 - <2 hrs | | 24.8 (383) | 1.63 (1.27 to 2.10) | 1.74 (1.36 to 2.22) | 26.7 (229) | 1.72 (1.25 to 2.37) | 1.73 (1.27 to 2.36) | 22.7 (154) | 1.71 (1.11 to 2.61) | 1.77 (1.18 to 2.67) |
| ≥ 2 hrs | | 33.5 (1,350) | 2.63 (2.11 to 3.28) | 2.79 (2.23 to 3.48) | 35.6 (529) | 2.87 (2.16 to 3.81) | 2.74 (2.09 to 3.58) | 32.3 (830) | 2.91 (1.95 to 4.34) | 2.89 (1.97 to 4.25) |
| **C. Current dual use (ref: never used cigarette or e-cigarette or tried once)** | | | | | | | | | | |
| No social media use | | 4.2 (29) | 0.93 (0.46 to 1.88) | 0.88 (0.45 to 1.72) | 5.1 (24) | 0.99 (0.44 to 2.22) | 1.00 (0.47 to 2.13) | 2.2 (4) | 0.62 (0.18 to 2.16) | 0.71 (0.20 to 2.55) |
| 1 - <30 mins | | 4.3 (46) | 1.00 | 1.00 | 4.8 (34) | 1.00 | 1.00 | 3.3 (11) | 1.00 | 1.00 |
| 30 mins - <1 hr | | 6.0 (68) | 1.52 (0.92 to 2.52) | 1.69 (1.03 to 2.77) | 6.9 (50) | 1.66 (0.91 to 3.03) | 1.73 (0.96 to 3.12) | 4.4 (18) | 1.38 (0.61 to 3.10) | 1.67 (0.72 to 3.86) |
| 1 - <2 hrs | | 8.7 (125) | 2.35 (1.54 to 3.59) | 2.72 (1.79 to 4.13) | 11.4 (83) | 2.89 (1.71 to 4.88) | 2.87 (1.73 to 4.76) | 5.8 (41) | 1.97 (0.95 to 4.06) | 2.22 (1.03 to 4.80) |
| ≥ 2 hrs | | 10.5 (405) | 3.39 (2.31 to 4.98) | 4.11 (2.77 to 6.08) | 14.8 (204) | 4.71 (2.95 to 7.51) | 4.41 (2.73 to 7.11) | 8.1 (201) | 3.32 (1.70 to 6.50) | 3.42 (1.67 to 6.99) |

**Legend:** Questionnaire imputed sample: n = 8,987 (weighted sample: n = 6,175). ^a^ Adjusted for sex, ethnicity, parental cigarette use, parental e-cigarette use, parenting style, previous cigarette use, previous e-cigarette use, anti-social behaviour, previous alcohol use, urbanicity, age, number of siblings in household, maternal age at participant birth, in-person activities, cognitive ability, mental health, risk-taking, and socioeconomic circumstances (family structure, household income, highest parental education in household, highest parental occupation in household, and area-level deprivation). Total population estimates adjusted for sex; sex stratified estimates are not. Values may not add up due to rounding. Abbreviations: AOR = Adjusted odds ratio; ARRR = Adjusted relative risk ratio; CI = Confidence interval; Hr/s = Hour/s; Min/s = Minute/s; n = Number of participants; OR = Odds ratio; Ref = Reference category, and RRR = Relative risk ratio.

Table-G2. Average time spent on social media across a normal weekday and weekend day on risk of (A) cigarette , (B) e-cigarette, and (C) dual use within the time-use-diary imputed sample, stratified by sex

|  |  | **Total population (n = 2,520)** | | | **Males (n = 1,123)** | | | **Females (n = 1,397)** | | |
| --- | --- | --- | --- | --- | --- | --- | --- | --- | --- | --- |
|  |  | **Weighted prevalence % (observed n with outcome)** | **OR (95% CI)** | **AOR (95% CI)^a^** | **Weighted prevalence %  (observed n with outcome)** | **OR (95% CI)** | **AOR (95% CI)^a^** | **Weighted prevalence % (observed n with outcome)** | **OR (95% CI)** | **AOR (95% CI)^a^** |
| **Average time spent on social media across a normal weekday and weekend day (time-use-diary)** | | | | | | | | | | |
| **A. Current or former cigarette use (ref: never used cigarette or tried once)** | | | | | | | | | | |
| No social media use | | 23.5 (239) | 1.28 (0.93 to 1.76) | 1.15 (0.83 to 1.60) | 24.2 (146) | 0.94 (0.61 to 1.45) | 0.96 (0.61 to 1.51) | 22.4 (93) | 1.65 (1.07 to 2.54) | 1.48 (0.93 to 2.36) |
| 1 - <30 mins | | 19.3 (94) | 1.00 | 1.00 | 25.3 (48) | 1.00 | 1.00 | 14.9 (46) | 1.00 | 1.00 |
| 30 mins - <1 hr | | 27.8 (95) | 1.60 (1.09 to 2.35) | 1.78 (1.22 to 2.60) | 28.1 (36) | 1.15 (0.66 to 2.01) | 1.29 (0.70 to 2.37) | 27.6 (60) | 2.18 (1.30 to 3.65) | 2.20 (1.33 to 3.65) |
| 1 - <2 hrs | | 31.8 (74) | 1.95 (1.29 to 2.94) | 1.87 (1.23 to 2.84) | 38.9 (27) | 1.88 (0.96 to 3.72) | 1.83 (0.93 to 3.59) | 28.0 (47) | 2.23 (1.33 to 3.73) | 2.15 (1.24 to 3.71) |
| ≥ 2 hrs | | 39.1 (58) | 2.67 (1.68 to 4.27) | 2.63 (1.68 to 4.12) | 40.0 (9) | 1.97 (0.67 to 5.73) | 1.69 (0.63 to 4.51) | 38.8 (49) | 3.64 (2.15 to 6.14) | 3.62 (2.13 to 6.18) |
| **B. Current or former e-cigarette use (ref: never used e-cigarette or tried once)** | | | | | | | | | | |
| No social media use | | 20.4 (204) | 1.19 (0.80 to 1.79) | 1.04 (0.71 to 1.51) | 22.8 (142) | 0.81 (0.47 to 1.40) | 0.86 (0.53 to 1.40) | 16.0 (62) | 1.55 (0.91 to 2.64) | 1.51 (0.88 to 2.60) |
| 1 - <30 mins | | 17.7 (73) | 1.00 | 1.00 | 26.6 (43) | 1.00 | 1.00 | 10.9 (30) | 1.00 | 1.00 |
| 30 mins - <1 hr | | 21.5 (72) | 1.28 (0.80 to 2.03) | 1.54 (1.00 to 2.38) | 29.2 (36) | 1.14 (0.56 to 2.30) | 1.31 (0.66 to 2.59) | 17.5 (36) | 1.73 (0.96 to 3.09) | 1.78 (0.99 to 3.22) |
| 1 - <2 hrs | | 25.2 (62) | 1.57 (0.98 to 2.50) | 1.56 (1.01 to 2.40) | 38.9 (29) | 1.76 (0.84 to 3.68) | 1.72 (0.85 to 3.51) | 17.8 (33) | 1.76 (0.94 to 3.30) | 1.69 (0.89 to 3.20) |
| ≥ 2 hrs | | 27.0 (46) | 1.72 (1.00 to 2.98) | 1.77 (1.07 to 2.93) | 40.6 (11) | 1.89 (0.63 to 5.64) | 1.50 (0.58 to 3.88) | 23.3 (35) | 2.48 (1.35 to 4.56) | 2.21 (1.21 to 4.05) |
|  |  | **Weighted prevalence % (observed n with outcome)** | **RRR (95% CI)** | **ARRR (95% CI)^a^** | **Weighted prevalence % (observed n with outcome)** | **RRR (95% CI)** | **ARRR (95% CI)^a^** | **Weighted prevalence % (observed n with outcome)** | **RRR (95% CI)** | **ARRR (95% CI)^a^** |
| **C. Current or former cigarette or e-cigarette use (ref: never used cigarette or e-cigarette or tried once)** | | | | | | | | | | |
| No social media use | | 22.1 (232) | 1.21 (0.83 to 1.74) | 1.05 (0.75 to 1.46) | 23.9 (150) | 0.85 (0.50 to 1.45) | 0.89 (0.58 to 1.35) | 18.8 (82) | 1.50 (0.95 to 2.35) | 1.36 (0.85 to 2.17) |
| 1 - <30 mins | | 19.3 (83) | 1.00 | 1.00 | 26.6 (40) | 1.00 | 1.00 | 13.8 (42) | 1.00 | 1.00 |
| 30 mins - <1 hr | | 28.4 (101) | 1.68 (1.09 to 2.60) | 1.96 (1.31 to 2.93) | 33.9 (42) | 1.44(0.71 to 2.91) | 1.68 (0.86 to 3.26) | 25.5 (59) | 2.18 (1.31 to 3.64) | 2.23 (1.32 to 3.76) |
| 1 - <2 hrs | | 28.7 (71) | 1.82 (1.11 to 2.99) | 1.75 (1.10 to 2.78) | 35.8 (27) | 1.84 (0.77 to 4.37) | 1.67 (0.80 to 3.50) | 24.9 (44) | 2.17 (1.24 to 3.77) | 2.11 (1.17 to 3.82) |
| ≥ 2 hrs | | 32.5 (49) | 2.19 (1.28 to 3.77) | 2.17 (1.34 to 3.52) | 41.4 (10) | 2.03 (0.66 to 6.23) | 1.67 (0.66 to 4.22) | 30.1 (39) | 3.00 (1.68 to 5.35) | 2.85 (1.65 to 4.93) |
| **C. Current dual use (ref: never used cigarette or e-cigarette or tried once)** | | | | | | | | | | |
| No social media use | | 6.9 (67) | 1.28 (0.78 to 2.10) | 1.19 (0.68 to 2.09) | 6.8 (41) | 0.89 (0.47 to 1.70) | 0.97 (0.42 to 2.25) | 7.3 (26) | 1.75 (0.82 to 3.73) | 1.64 (0.70 to 3.83) |
| 1 - <30 mins | | 5.7 (28) | 1.00 | 1.00 | 7.3 (16) | 1.00 | 1.00 | 4.6 (12) | 1.00 | 1.00 |
| 30 mins - <1 hr | | 6.3 (19) | 1.25 (0.63 to 2.47) | 1.42 (0.71 to 2.86) | 7.7 (9) | 1.19 (0.46 to 3.08) | 1.27 (0.41 to 3.97) | 5.5 (9) | 1.42 (0.52 to 3.88) | 1.38 (0.53 to 3.60) |
| 1 - <2 hrs | | 10.3 (22) | 2.20 (1.21 to 3.99) | 2.24 (1.14 to 4.41) | 15.8 (11) | 2.95 (1.15 to 7.55) | 3.60 (1.24 to 10.4) | 7.3 (11) | 1.92 (0.76 to 4.87) | 1.71 (0.67 to 4.34) |
| ≥ 2 hrs | | 10.0 (17) | 2.26 (1.17 to 4.36) | 2.37 (1.18 to 4.76) | 8.0 (3) | 1.44 (0.37 to 5.53) | 1.16 (0.29 to 4.70) | 10.5 (14) | 3.15 (1.34 to 7.38) | 2.92 (1.21 to 7.08) |

**Legend:** Time-use-diary imputed sample: n = 2,520 (weighted sample: n = 5,005). ^a^ Adjusted for sex, ethnicity, parental cigarette use, parental e-cigarette use, parenting style, previous cigarette use, previous e-cigarette use, anti-social behaviour, previous alcohol use, urbanicity, age, number of siblings in household, maternal age at participant birth, in-person activities, cognitive ability, mental health, risk-taking, and socioeconomic circumstances (family structure, household income, highest parental education in household, highest parental occupation in household, and area-level deprivation). Total population estimates adjusted for sex; sex stratified estimates are not. Values may not add up due to rounding. Abbreviations: AOR = Adjusted odds ratio; ARRR = Adjusted relative risk ratio; CI = Confidence interval; Hr/s = Hour/s; Min/s = Minute/s; n = Number of participants; OR = Odds ratio; Ref = Reference category, and RRR = Relative risk ratio.

Table-G3. Time spent on social media on a normal weekday on risk of (A) cigarette, (B) e-cigarette, and (C) dual use within the questionnaire complete case sample

|  | **Questionnaire complete case sample (n = 6,234)** | | |
| --- | --- | --- | --- |
|  | **Weighted prevalence % (observed n with outcome)** | **OR (95% CI)** | **AOR (95% CI)^a^** |
| **Time spent on social media on a normal weekday (questionnaire)** | | | |
| **A. Current or former cigarette use (ref: never used cigarette or tried once)** | | | |
| No social media use | 15.2 (62) | 0.93 (0.63 to 1.36) | 0.90 (0.62 to 1.32) |
| 1 - <30 min | 16.2 (122) | 1.00 | 1.00 |
| 30 min - <1 hr | 23.8 (214) | 1.61 (1.22 to 2.12) | 1.66 (1.25 to 2.21) |
| 1 - <2 hrs | 26.6 (286) | 1.87 (1.45 to 2.42) | 1.92 (1.47 to 2.50) |
| ≥ 2 hrs | 37.8 (1,046) | 3.14 (2.52 to 3.91) | 3.08 (2.42 to 3.91) |
| **B. Current or former e-cigarette use (ref: never used e-cigarette or tried once)** | | | |
| No social media use | 12.2 (49) | 0.94 (0.59 to 1.49) | 0.84 (0.55 to 1.26) |
| 1 - <30 min | 12.9 (101) | 1.00 | 1.00 |
| 30 min - <1 hr | 20.5 (186) | 1.74 (1.31 to 2.31) | 1.91 (1.42 to 2.58) |
| 1 - <2 hrs | 21.0 (237) | 1.78 (1.36 to 2.33) | 2.04 (1.53 to 2.73) |
| ≥ 2 hrs | 28.8 (816) | 2.72 (2.16 to 3.43) | 3.35 (2.61 to 4.30) |
|  | **Weighted prevalence % (observed n with outcome)** | **RRR (95% CI)** | **ARRR (95% CI)^a^** |
| **C. Current or former cigarette or e-cigarette use (ref: never used cigarette or e-cigarette or tried once)** | | | |
| No social media use | 14.7 (57) | 0.90 (0.58 to 1.38) | 0.84 (0.57 to 1.25) |
| 1 - <30 min | 16.1 (124) | 1.00 | 1.00 |
| 30 min - <1 hr | 23.8 (217) | 1.69 (1.29 to 2.22) | 1.78 (1.35 to 2.33) |
| 1 - <2 hrs | 24.5 (271) | 1.80 (1.40 to 2.31) | 1.88 (1.46 to 2.43) |
| ≥ 2 hrs | 33.6 (949) | 2.98 (2.39 to 3.72) | 3.12 (2.48 to 3.93) |
| **C. Current dual use (ref: never used cigarette or e-cigarette or tried once)** | | | |
| No social media use | 3.9 (18) | 0.91 (0.47 to 1.77) | 0.84 (0.41 to 1.69) |
| 1 - <30 min | 4.2 (31) | 1.00 | 1.00 |
| 30 min - <1 hr | 6.3 (55) | 1.73 (1.07 to 2.78) | 1.92 (1.16 to 3.17) |
| 1 - <2 hrs | 8.2 (86) | 2.32 (1.53 to 3.51) | 2.71 (1.75 to 4.21) |
| ≥ 2 hrs | 10.5 (298) | 3.58 (2.44 to 5.25) | 4.27 (2.81 to 6.50) |

**Legend:** Questionnaire complete case sample: n = 6,234 (weighted sample: n = 4,484). ^a^ Adjusted for sex, ethnicity, parental cigarette use, parental e-cigarette use, parenting style, previous cigarette use, previous e-cigarette use, anti-social behaviour, previous alcohol use, urbanicity, age, number of siblings in household, maternal age at participant birth, in-person activities, cognitive ability, mental health, risk-taking, and socioeconomic circumstances (family structure, household income, highest parental education in household, highest parental occupation in household, and area-level deprivation). Values may not add up due to rounding. Abbreviations: AOR = Adjusted odds ratio; ARRR = Adjusted relative risk ratio; CI = Confidence interval; Hr/s = Hour/s; Min/s = Minute/s; n = Number of participants; OR = Odds ratio, Ref = Reference category, and RRR = Relative risk ratio.

Table-G4 Average time spent on social media across a normal weekday and weekend day on risk of (A) cigarette, (B) e-cigarette, and (C) dual use within the time-use-diary complete case sample

|  | | **Time-use-diary complete case sample (n = 2,109)** | | |
| --- | --- | --- | --- | --- |
|  | | **Weighted prevalence % (observed n with outcome)** | **OR (95% CI)** | **AOR (95% CI)^a^** |
| **Average time spent on social media across a normal weekday and weekend day (time-use-diary)** | | | | |
| **A. Current or former cigarette use (ref: never used cigarette or tried once)** | | | | |
| No social media use | | 23.3 (202) | 1.27 (0.92 to 1.77) | 1.18 (0.84 to 1.65) |
| 1 - <30 mins | | 19.2 (78) | 1.00 | 1.00 |
| 30 mins - <1 hr | | 24.0 (74) | 1.33 (0.89 to 1.97) | 1.47 (0.99 to 2.18) |
| 1 - <2 hrs | | 34.1 (66) | 2.17 (1.40 to 3.38) | 2.02 (1.28 to 3.18) |
| ≥ 2 hrs | | 42.6 (55) | 3.12 (1.92 to 5.06) | 3.08 (1.95 to 4.85) |
| **B. Current or former e-cigarette use (ref: never used e-cigarette or tried once)** | | | | |
| No social media use | | 19.7 (164) | 1.26 (0.80 to 2.00) | 1.19 (0.79 to 1.79) |
| 1 - <30 mins | | 16.3 (56) | 1.00 | 1.00 |
| 30 mins - <1 hr | | 20.8 (57) | 1.35 (0.79 to 2.29) | 1.66 (1.02 to 2.70) |
| 1 - <2 hrs | | 25.6 (51) | 1.77 (1.05 to 2.98) | 1.78 (1.12 to 2.82) |
| ≥ 2 hrs | | 27.0 (39) | 1.91 (1.02 to 3.55) | 1.86 (1.06 to 3.26) |
|  |  | **Weighted prevalence % (observed n with outcome)** | **RRR (95% CI)** | **ARRR (95% CI)^a^** |
| **C. Current or former cigarette or e-cigarette use (ref: never used cigarette or e-cigarette or tried once)** | | | | |
| No social media use | | 21.5 (196) | 1.23 (0.82 to 1.86) | 1.11 (0.78 to 1.59) |
| 1 - <30 mins | | 18.5 (65) | 1.00 | 1.00 |
| 30 mins - <1 hr | | 26.8 (85) | 1.62 (1.01 to 2.58) | 1.86 (1.22 to 2.82) |
| 1 - <2 hrs | | 29.4 (59) | 2.04 (1.18 to 3.50) | 1.88 (1.16 to 3.06) |
| ≥ 2 hrs | | 32.9 (42) | 2.40 (1.30 to 4.40) | 2.30 (1.35 to 3.91) |
| **C. Current dual use (ref: never used cigarette or e-cigarette or tried once)** | | | | |
| No social media use | | 7.0 (53) | 1.28 (0.74 to 2.20) | 1.21 (0.65 to 2.28) |
| 1 - <30 mins | | 5.8 (24) | 1.00 | 1.00 |
| 30 mins - <1 hr | | 5.0 (12) | 0.96 (0.42 to 2.19) | 1.09 (0.46 to 2.59) |
| 1 - <2 hrs | | 11.3 (21) | 2.47 (1.31 to 4.64) | 2.50 (1.17 to 5.31) |
| ≥ 2 hrs | | 10.7 (16) | 2.47 (1.25 to 4.86) | 2.58 (1.19 to 5.58) |

Legend: Time-use-diary complete case sample: n = 2,109 (weighted sample: n = 4,199). ^a^ Adjusted for sex, ethnicity, parental cigarette use, parental e-cigarette use, parenting style, previous cigarette use, previous e-cigarette use, anti-social behaviour, previous alcohol use, urbanicity, age, number of siblings in household, maternal age at participant birth, in-person activities, cognitive ability, mental health, risk-taking, and socioeconomic circumstances (family structure, household income, highest parental education in household, highest parental occupation in household, and area-level deprivation). Values may not add up due to rounding. Abbreviations: AOR = Adjusted odds ratio; ARRR = Adjusted relative risk ratio; CI = Confidence interval; Hr/s = Hour/s; Min/s = Minute/s; n = Number of participants; OR = Odds ratio; Ref = Reference category, and RRR = Relative risk ratio

Table-G5. Time spent on social media on a normal weekday on risk of (A) former cigarette use, (B) current cigarette use, (C) former e-cigarette use, and (D) current e-cigarette use, within the questionnaire imputed sample

|  |  | **Questionnaire imputed sample (n = 8,987)** | | |
| --- | --- | --- | --- | --- |
|  |  | **Weighted prevalence % (observed n with outcome)** | **OR (95% CI)** | **AOR (95% CI)^a^** |
| **Time spent on social media on a normal weekday (questionnaire)** | | | | |
| **A. Former cigarette use (ref: never used cigarette or tried once)** | | | | |
| No social media use | | 3.3 (26) | 0.59 (0.33 to 1.04) | 0.58 (0.32 to 1.03) |
| 1 min - <1 hr | | 5.2 (119) | 1.00 | 1.00 |
| 1 - <2 hrs | | 4.8 (83) | 1.00 (0.71 to 1.40) | 0.96 (0.68 to 1.35) |
| ≥ 2 hrs | | 9.6 (376) | 2.31 (1.78 to 3.01) | 2.08 (1.59 to 2.72) |
| **B. Current cigarette use (ref: never used cigarette or tried once)** | | | | |
| No social media use | | 11.6 (68) | 0.72 (0.47 to 1.09) | 0.69 (0.46 to 1.02) |
| 1 min - <1 hr | | 15.2 (330) | 1.00 | 1.00 |
| 1 - <2 hrs | | 22.1 (312) | 1.59 (1.30 to 1.95) | 1.59 (1.30 to 1.95) |
| ≥ 2 hrs | | 27.6 (1,070) | 2.31 (1.95 to 2.74) | 2.25 (1.88 to 2.70) |
| **C. Former e-cigarette use (ref: never used e-cigarette or tried once)** | | | | |
| No social media use | | 6.5 (39) | 0.69 (0.40 to 1.17) | 0.62 (0.37 to 1.04) |
| 1 min - <1 hr | | 9.0 (218) | 1.00 | 1.00 |
| 1 - <2 hrs | | 11.0 (175) | 1.30 (1.00 to 1.70) | 1.39 (1.05 to 1.83) |
| ≥ 2 hrs | | 14.6 (599) | 1.92 (1.53 to 2.40) | 2.14 (1.69 to 2.72) |
| **D. Current e-cigarette use (ref: never used e-cigarette or tried once)** | | | | |
| No social media use | | 7.4 (49) | 0.82 (0.49 to 1.36) | 0.73 (0.46 to 1.15) |
| 1 min - <1 hr | | 8.6 (200) | 1.00 | 1.00 |
| 1 - <2 hrs | | 11.4 (177) | 1.40 (1.10 to 1.79) | 1.56 (1.23 to 1.99) |
| ≥ 2 hrs | | 15.1 (602) | 2.06 (1.68 to 2.52) | 2.50 (2.03 to 3.07) |

**Legend:** Questionnaire imputed sample: n = 8,987 (weighted sample: n = 6,175). ^a^ Adjusted for sex, ethnicity, parental cigarette use, parental e-cigarette use, parenting style, previous cigarette use, previous e-cigarette use, anti-social behaviour, previous alcohol use, urbanicity, age, number of siblings in household, maternal age at participant birth, in-person activities, cognitive ability, mental health, risk-taking, and socioeconomic circumstances (family structure, household income, highest parental education in household, highest parental occupation in household, and area-level deprivation). Values may not add up due to rounding. Abbreviations: AOR = Adjusted odds ratio; CI = Confidence interval; Hr/s = Hour/s; Min/s = Minute/s; n = Number of participants; OR = Odds ratio, and Ref = Reference category.

Table-G6. Comparison of estimates for time spent on social media on a normal weekday on risk of (A) cigarette, (B) e-cigarette, and (C) dual use within the questionnaire and time-use-diary imputed samples

|  | **Questionnaire imputed sample (n = 8,987)** | | | **Time-use-diary imputed sample (n = 2,520)** | | |
| --- | --- | --- | --- | --- | --- | --- |
|  | **Weighted prevalence % (observed n with outcome)** | **OR (95% CI)** | **AOR (95% CI)^a^** | **Weighted prevalence % (observed n with outcome)** | **OR (95% CI)** | **AOR (95% CI)^a^** |
| **Time spent on social media on a normal weekday** | | | | | | |
| **A. Current or former cigarette use (ref: never used cigarette or tried once)** | | | | | | |
| No social media use | 14.9 (94) | 0.83 (0.56 to 1.22) | 0.82 (0.57 to 1.18) | 24.0 (324) | 1.34 (0.86 to 2.08) | 1.29 (0.81 to 2.07) |
| 1 - <30 mins | 17.4 (175) | 1.00 | 1.00 | 19.1 (41) | 1.00 | 1.00 |
| 30 mins - <1 hr | 22.9 (275) | 1.41 (1.05 to 1.89) | 1.48 (1.11 to 1.97) | 23.9 (56) | 1.33 (0.78 to 2.26) | 1.25 (0.75, 2.10) |
| 1 - <2 hrs | 26.9 (395) | 1.74 (1.35 to 2.26) | 1.78 (1.38 to 2.29) | 33.1 (82) | 2.09 (1.28 to 3.43) | 2.21 (1.32 to 3.70) |
| ≥ 2 hrs | 37.2 (1,445) | 2.80 (2.23 to 3.52) | 2.76 (2.19 to 3.48) | 34.9 (58) | 2.67 (1.33 to 3.87) | 2.30 (1.31 to 3.95) |
| **B. Current or former e-cigarette use (ref: never used e-cigarette or tried once)** | | | | | | |
| No social media use | 13.9 (88) | 1.01 (0.66 to 1.53) | 0.94 (0.63 to 1.39) | 21.3 (284) | 1.47 (0.87 to 2.49) | 1.24 (0.73 to 2.13) |
| 1 - <30 min | 13.8 (157) | 1.00 | 1.00 | 15.5 (29) | 1.00 | 1.00 |
| 30 mins - <1 hr | 20.9 (262) | 1.65 (1.24 to 2.20) | 1.79 (1.34 to 2.39) | 15.8 (37) | 1.02 (0.54 to 1.94) | 0.91 (0.49 to 1.69) |
| 1 - <2 hrs | 22.4 (351) | 1.80 (1.42 to 2.29) | 2.06 (1.61 to 2.64) | 25.1 (60) | 1.82 (0.99 to 3.34) | 1.80 (1.00 to 3.27) |
| ≥ 2 hrs | 29.8 (1,200) | 2.65 (2.14 to 3.29) | 3.24 (2.59 to 4.05) | 26.0 (47) | 1.91 (1.05 to 3.47) | 1.78 (1.01 to 3.17) |
|  | **Weighted prevalence % (observed n with outcome)** | **RRR (95% CI)** | **ARRR (95% CI)^a^** | **Weighted prevalence % (observed n with outcome)** | **RRR (95% CI)** | **ARRR (95% CI)^a^** |
| **C. Current or former cigarette or e-cigarette use (ref: never used cigarette or e-cigarette or tried once)** | | | | | | |
| No social media use | 15.0 (95) | 0.82 (0.56 to 1.19) | 0.78 (0.55 to 1.12) | 23.2 (313) | 1.39 (0.89 to 2.17) | 1.20 (0.74 to 1.94) |
| 1 - <30 min | 17.7 (186) | 1.00 | 1.00 | 18.3 (37) | 1.00 | 1.00 |
| 30 mins - <1 hr | 23.8 (302) | 1.49 (1.13 to 1.97) | 1.58 (1.21 to 2.07) | 21.3 (50) | 1.20 (0.68 to 2.12) | 1.10 (0.64 to 1.89) |
| 1 - <2 hrs | 24.8 (383) | 1.63 (1.27 to 2.10) | 1.74 (1.36 to 2.22) | 29.8 (79) | 2.04 (1.19 to 3.50) | 2.03 (1.18 to 3.51) |
| ≥ 2 hrs | 33.5 (1,350) | 2.63 (2.11 to 3.28) | 2.79 (2.23 to 3.48) | 31.6 (56) | 2.21 (1.27 to 3.84) | 2.02 (1.14 to 3.56) |
| **C. Current dual use (ref: never used cigarette or e-cigarette or tried once)** | | | | | | |
| No social media use | 4.2 (29) | 0.93 (0.46 to 1.88) | 0.88 (0.45 to 1.72) | 7.3 (95) | 1.52 (0.78 to 2.95) | 1.46 (0.68 to 3.11) |
| 1 - <30 min | 4.3 (46) | 1.00 | 1.00 | 5.3 (11) | 1.00 | 1.00 |
| 30 mins - <1 hr | 6.0 (68) | 1.52 (0.92 to 2.52) | 1.69 (1.03 to 2.77) | 4.8 (12) | 0.93 (0.39 to 2.26) | 0.91 (0.34 to 2.41) |
| 1 - <2 hrs | 8.7 (125) | 2.35 (1.54 to 3.59) | 2.72 (1.79 to 4.13) | 9.3 (19) | 2.22 (0.99 to 5.00) | 2.38 (1.00 to 5.66) |
| ≥ 2 hrs | 10.5 (405) | 3.39 (2.31 to 4.98) | 4.11 (2.77 to 6.08) | 9.0 (15) | 2.18 (0.94 to 5.04) | 2.27 (0.95 to 5.43) |

**Legend:** Questionnaire imputed sample n = 8,987 (weighted sample: n = 6,175). Time-use-diary imputed sample: n = 2,520 (weighted sample: n = 5,005). ^a^ Adjusted for sex, ethnicity, parental cigarette use, parental e-cigarette use, parenting style, previous cigarette use, previous e-cigarette use, anti-social behaviour, previous alcohol use, urbanicity, age, number of siblings in household, maternal age at participant birth, in-person activities, cognitive ability, mental health, risk-taking, and socioeconomic circumstances (family structure, household income, highest parental education in household, highest parental occupation in household, and area-level deprivation). Values may not add up due to rounding. Abbreviations: AOR = Adjusted odds ratio; ARRR = Adjusted relative risk ratio; CI = Confidence interval; Hr/s = Hour/s; Min/s = Minute/s; n = Number of participants; OR = Odds ratio; Ref = Reference category, and RRR = Relative risk ratio.

Table-G7. Time spent on social media on a normal weekday on risk of (A) cigarette, (B) e-cigarette, and (C) dual use within the questionnaire imputed sample with additional adjustment for baseline outcome measures and previous social media use

|  | **Questionnaire imputed sample (n = 8,987)** | | | | |
| --- | --- | --- | --- | --- | --- |
|  | **Weighted prevalence % (observed n with outcome)** | **OR (95% CI)** | **AOR (95% CI)^a^** | **AOR (95% CI)^b^** | **AOR (95% CI)^c^** |
| **Time spent on social media on a normal weekday (questionnaire)** | | | | | |
| **A. Current or former cigarette use (ref: never used cigarette or tried once)** | | | | | |
| No social media use | 14.9 (94) | 0.83 (0.56 to 1.22) | 0.82 (0.57 to 1.18) | 0.79 (0.54 to 1.15) | 0.86 (0.60 to 1.23) |
| 1 - <30 mins | 17.4 (175) | 1.00 | 1.00 | 1.00 | 1.00 |
| 30 mins - <1 hr | 22.9 (275) | 1.41 (1.05 to 1.89) | 1.48 (1.11 to 1.97) | 1.42 (1.06 to 1.90) | 1.46 (1.10 to 1.94) |
| 1 - <2 hrs | 26.9 (395) | 1.74 (1.35 to 2.26) | 1.78 (1.38 to 2.29) | 1.71 (1.32 to 2.22) | 1.75 (1.35 to 2.25) |
| ≥ 2 hrs | 37.2 (1,445) | 2.80 (2.23 to 3.52) | 2.76 (2.19 to 3.48) | 2.24 (1.77 to 2.84) | 2.66 (2.11 to 3.34) |
| **B. Current or former e-cigarette use (ref: never used e-cigarette or tried once)** | | | | | |
| No social media use | 13.9 (88) | 1.01 (0.66 to 1.53) | 0.94 (0.63 to 1.39) | 0.95 (0.64 to 1.40) | 0.99 (0.67 to 1.47) |
| 1 - <30 mins | 13.8 (157) | 1.00 | 1.00 | 1.00 | 1.00 |
| 30 mins - <1 hr | 20.9 (262) | 1.65 (1.24 to 2.20) | 1.79 (1.34 to 2.39) | 1.61 (1.22 to 2.13) | 1.76 (1.32 to 2.34) |
| 1 - <2 hrs | 22.4 (351) | 1.80 (1.42 to 2.29) | 2.06 (1.61 to 2.64) | 1.86 (1.46 to 2.38) | 2.02 (1.57 to 2.58) |
| ≥ 2 hrs | 29.8 (1,200) | 2.65 (2.14 to 3.29) | 3.24 (2.59 to 4.05) | 2.54 (2.03 to 3.18) | 3.10 (2.49 to 3.88) |
|  | **Weighted prevalence % (observed n with outcome)** | **RRR (95% CI)** | **ARRR (95% CI)^a^** | **ARRR (95% CI)^b^** | **ARRR (95% CI)^c^** |
| **C. Current or former cigarette or e-cigarette use (ref: never used cigarette or e-cigarette or tried once)** | | | | | |
| No social media use | 15.0 (95) | 0.82 (0.56 to 1.19) | 0.78 (0.55 to 1.12) | 0.78 (0.54 to 1.12) | 0.81 (0.56 to 1.15) |
| 1 - <30 mins | 17.7 (186) | 1.00 | 1.00 | 1.00 | 1.00 |
| 30 mins - <1 hr | 23.8 (302) | 1.49 (1.13 to 1.97) | 1.58 (1.21 to 2.07) | 1.47 (1.11 to 1.94) | 1.56 (1.19 to 2.05) |
| 1 - <2 hrs | 24.8 (383) | 1.63 (1.27 to 2.10) | 1.74 (1.36 to 2.22) | 1.63 (1.27 to 2.10) | 1.71 (1.34 to 2.19) |
| ≥ 2 hrs | 33.5 (1,350) | 2.63 (2.11 to 3.28) | 2.79 (2.23 to 3.48) | 2.16 (1.72 to 2.71) | 2.70 (2.17 to 3.37) |
| **C. Current dual use (ref: never used cigarette or e-cigarette or tried once)** | | | | | |
| No social media use | 4.2 (29) | 0.93 (0.46 to 1.88) | 0.88 (0.45 to 1.72) | 0.88 (0.44 to 1.77) | 0.97 (0.50 to 1.90) |
| 1 - <30 mins | 4.3 (46) | 1.00 | 1.00 | 1.00 | 1.00 |
| 30 mins - <1 hr | 6.0 (68) | 1.52 (0.92 to 2.52) | 1.69 (1.03 to 2.77) | 1.47 (0.91 to 2.40) | 1.63 (0.99 to 2.67) |
| 1 - <2 hrs | 8.7 (125) | 2.35 (1.54 to 3.59) | 2.72 (1.79 to 4.13) | 2.45 (1.61 to 3.74) | 2.59 (1.70 to 3.96) |
| ≥ 2 hrs | 10.5 (405) | 3.39 (2.31 to 4.98) | 4.11 (2.77 to 6.08) | 2.92 (1.97 to 4.33) | 3.80 (2.56 to 5.65) |

**Legend:** Questionnaire imputed sample n = 8,987 (weighted sample: n = 6,175). ^a^ Adjusted for sex, ethnicity, parental cigarette use, parental e-cigarette use, parenting style, previous cigarette use, previous e-cigarette use, anti-social behaviour, previous alcohol use, urbanicity, age, number of siblings in household, maternal age at participant birth, in-person activities, cognitive ability, mental health, risk-taking, and socioeconomic circumstances (family structure, household income, highest parental education in household, highest parental occupation in household, and area-level deprivation). ^b^ Additional adjustment for baseline cigarette use (age 14-years) in model A; additional adjustment for baseline e-cigarette use (age 14-years) in model B, and additional adjustment for both baseline cigarette and e-cigarette use (age 14-years) in model C. ^c^ Additional adjustment for previous social media use (age 11-years) in all models. Values may not add up due to rounding. Abbreviations: AOR = Adjusted odds ratio; ARRR = Adjusted relative risk ratio; CI = Confidence interval; Hr/s = Hour/s; Min/s = Minute/s; n = Number of participants; OR = Odds ratio; Ref = Reference category, and RRR = Relative risk ratio.

Table-G8. Average time spent on social media across a normal weekday and weekend day on risk of (A) cigarette, (B) e-cigarette, and (C) dual use within the time-use-diary imputed sample with additional adjustment for baseline outcome measures and previous social media use

|  |  | **Time-use-diary imputed sample (n = 2,520)** | | | | |
| --- | --- | --- | --- | --- | --- | --- |
|  |  | **Weighted prevalence % (observed n with outcome)** | **OR (95% CI)** | **AOR (95% CI)^a^** | **AOR (95% CI)^b^** | **AOR (95% CI)^c^** |
| **Average time spent on social media across a normal weekday and weekend day (time-use-diary)** | | | | |  |  |
| **A. Current or former cigarette use (ref: never used cigarette or tried once)** | | | | |  |  |
| No social media use | | 23.5 (239) | 1.28 (0.93 to 1.76) | 1.15 (0.83 to 1.60) | 1.12 (0.78 to 1.60) | 1.18 (0.85 to 1.63) |
| 1 - <30 mins | | 19.3 (94) | 1.00 | 1.00 | 1.00 | 1.00 |
| 30 mins - <1 hr | | 27.8 (95) | 1.60 (1.09 to 2.35) | 1.78 (1.22 to 2.60) | 1.81 (1.22 to 2.67) | 1.74 (1.20 to 2.52) |
| 1 - <2 hrs | | 31.8 (74) | 1.95 (1.29 to 2.94) | 1.87 (1.23 to 2.84) | 1.52 (0.97 to 2.37) | 1.88 (1.25 to 2.85) |
| ≥ 2 hrs | | 39.1 (58) | 2.67 (1.68 to 4.27) | 2.63 (1.68 to 4.12) | 2.31 (1.45 to 3.70) | 2.44 (1.56 to 3.82) |
| **B. Current or former e-cigarette use (ref: never used e-cigarette or tried once)** | | | | | | |
| No social media use | | 20.4 (204) | 1.19 (0.80 to 1.79) | 1.04 (0.71 to 1.51) | 0.96 (0.65 to 1.44) | 1.08 (0.75 to 1.56) |
| 1 - <30 mins | | 17.7 (73) | 1.00 | 1.00 | 1.00 | 1.00 |
| 30 mins - <1 hr | | 21.5 (72) | 1.28 (0.80 to 2.03) | 1.54 (1.00 to 2.38) | 1.58 (1.02 to 2.46) | 1.53 (0.99 to 2.37) |
| 1 - <2 hrs | | 25.2 (62) | 1.57 (0.98 to 2.50) | 1.56 (1.01 to 2.40) | 1.29 (0.81 to 2.05) | 1.59 (1.04 to 2.43) |
| ≥ 2 hrs | | 27.0 (46) | 1.72 (1.00 to 2.98) | 1.77 (1.07 to 2.93) | 1.58 (0.95 to 2.62) | 1.67 (1.01 to 2.77) |
|  |  | **Weighted prevalence % (observed n with outcome)** | **RRR (95% CI)** | **ARRR (95% CI)^a^** | **ARRR (95% CI)^b^** | **ARRR (95% CI)^c^** |
| **C. Current or former cigarette or e-cigarette use (ref: never used cigarette or e-cigarette or tried once)** | | | | | | |
| No social media use | | 22.1 (232) | 1.21 (0.83 to 1.74) | 1.05 (0.75 to 1.46) | 1.01 (0.70 to 1.44) | 1.06 (0.77 to 1.49) |
| 1 - <30 mins | | 19.3 (83) | 1.00 | 1.00 | 1.00 | 1.00 |
| 30 mins - <1 hr | | 28.4 (101) | 1.68 (1.09 to 2.60) | 1.96 (1.31 to 2.93) | 2.00 (1.33 to 3.02) | 1.92 (1.30 to 2.84) |
| 1 - <2 hrs | | 28.7 (71) | 1.82 (1.11 to 2.99) | 1.75 (1.10 to 2.78) | 1.43 (0.89 to 2.29) | 1.76 (1.11 to 2.78) |
| ≥ 2 hrs | | 32.5 (49) | 2.19 (1.28 to 3.77) | 2.17 (1.34 to 3.52) | 1.79 (1.09 to 2.95) | 2.00 (1.24 to 3.25) |
| **C. Current dual use (ref: never used cigarette or e-cigarette or tried once)** | | | | | | |
| No social media use | | 6.9 (67) | 1.28 (0.78 to 2.10) | 1.19 (0.68 to 2.09) | 1.07 (0.59 to 1.95) | 1.24 (0.70 to 2.18) |
| 1 - <30 mins | | 5.7 (28) | 1.00 | 1.00 | 1.00 | 1.00 |
| 30 mins - <1 hr | | 6.3 (19) | 1.25 (0.63 to 2.47) | 1.42 (0.71 to 2.86) | 1.53 (0.76 to 3.10) | 1.42 (0.70 to 2.87) |
| 1 - <2 hrs | | 10.3 (22) | 2.20 (1.21 to 3.99) | 2.24 (1.14 to 4.41) | 1.80 (0.89 to 3.63) | 2.31 (1.20 to 4.47) |
| ≥ 2 hrs | | 10.0 (17) | 2.26 (1.17 to 4.36) | 2.37 (1.18 to 4.76) | 2.23 (1.08 to 4.64) | 2.29 (1.13 to 4.63) |

**Legend:** Time-use-diary imputed sample: n = 2,520 (weighted sample: n = 5,005). ^a^ Adjusted for sex, ethnicity, parental cigarette use, parental e-cigarette use, parenting style, previous cigarette use, previous e-cigarette use, anti-social behaviour, previous alcohol use, urbanicity, age, number of siblings in household, maternal age at participant birth, in-person activities, cognitive ability, mental health, risk-taking, and socioeconomic circumstances (family structure, household income, highest parental education in household, highest parental occupation in household, and area-level deprivation). ^b^ Additional adjustment for baseline cigarette use (age 14-years) in model A; additional adjustment for baseline e-cigarette use (age 14-years) in model B, and additional adjustment for both baseline cigarette and e-cigarette use (age 14-years) in model C. ^c^ Additional adjustment for previous social media use (age 11-years) in all models. Values may not add up due to rounding. Abbreviations: AOR = Adjusted odds ratio; ARRR = Adjusted relative risk ratio; CI = Confidence interval; Hr/s = Hour/s; Min/s = Minute/s; n = Number of participants; OR = Odds ratio; Ref = Reference category, and RRR = Relative risk ratio.

Table-G9. Time spent on social media on a normal weekday on risk of (A) cigarette, (B) e-cigarette, and (C) dual use within the questionnaire imputed sample replacing '1-<30 minutes' reference category with 'no social media use'

|  |  | **Questionnaire imputed sample (n = 8,987)** | | |
| --- | --- | --- | --- | --- |
|  |  | **Weighted prevalence % (observed n with outcome)** | **OR (95% CI)** | **AOR (95% CI)^a^** |
| **Time spent on social media on a normal weekday (questionnaire)** | | | | |
| **A. Current or former cigarette use (ref: never used cigarette or tried once)** | | | | |
| No social media use | | 14.9 (94) | 1.00 | 1.00 |
| 1 - <30 min | | 17.4 (175) | 1.21 (0.82 to 1.77) | 1.22 (0.85 to 1.75) |
| 30 min - <1 hr | | 22.9 (275) | 1.70 (1.15 to 2.52) | 1.81 (1.23 to 2.64) |
| 1 - <2 hrs | | 26.9 (395) | 2.10 (1.47 to 2.99) | 2.17 (1.53 to 3.08) |
| ≥ 2 hrs | | 37.2 (1,445) | 3.38 (2.42 to 4.72) | 3.36 (2.41 to 4.69) |
| **B. Current or former e-cigarette use (ref: never used e-cigarette or tried once)** | | | | |
| No social media use | | 13.9 (88) | 1.00 | 1.00 |
| 1 - <30 min | | 13.8 (157) | 0.99 (0.65 to 1.51) | 1.06 (0.72 to 1.58) |
| 30 min - <1 hr | | 20.9 (262) | 1.64 (1.10 to 2.46) | 1.91 (1.30 to 2.80) |
| 1 - <2 hrs | | 22.4 (351) | 1.79 (1.22 to 2.64) | 2.20 (1.53 to 3.15) |
| ≥ 2 hrs | | 29.8 (1,200) | 2.64 (1.85 to 3.77) | 3.45 (2.47 to 4.83) |
|  |  | **Weighted prevalence % (observed n with outcome)** | **RRR (95% CI)** | **ARRR (95% CI)^a^** |
| **C. Current or former cigarette or e-cigarette use (ref: never used cigarette or e-cigarette or tried once)** | | | | |
| No social media use | | 15.0 (95) | 1.00 | 1.00 |
| 1 - <30 min | | 17.7 (186) | 1.22 (0.84 to 1.79) | 1.28 (0.89 to 1.83) |
| 30 min - <1 hr | | 23.8 (302) | 1.83 (1.26 to 2.65) | 2.02 (1.42 to 2.89) |
| 1 - <2 hrs | | 24.8 (383) | 2.00 (1.41 to 2.83) | 2.22 (1.60 to 3.09) |
| ≥ 2 hrs | | 33.5 (1,350) | 3.22 (2.31 to 4.50) | 3.56 (2.59 to 4.90) |
| **C. Current dual use (ref: never used cigarette or e-cigarette or tried once)** | | | | |
| No social media use | | 4.2 (29) | 1.00 | 1.00 |
| 1 - <30 min | | 4.3 (46) | 1.08 (0.53 to 2.18) | 1.13 (0.58 to 2.21) |
| 30 min - <1 hr | | 6.0 (68) | 1.64 (0.82 to 3.27) | 1.91 (0.99 to 3.69) |
| 1 - <2 hrs | | 8.7 (125) | 2.53 (1.33 to 4.78) | 3.07 (1.66 to 5.69) |
| ≥ 2 hrs | | 10.5 (405) | 3.65 (2.04 to 6.53) | 4.65 (2.65 to 8.16) |

**Legend:** Questionnaire imputed sample: n = 8,987 (weighted sample: n = 6,175). ^a^ Adjusted for sex, ethnicity, parental cigarette use, parental e-cigarette use, parenting style, previous cigarette use, previous e-cigarette use, anti-social behaviour, previous alcohol use, urbanicity, age, number of siblings in household, maternal age at participant birth, in-person activities, cognitive ability, mental health, risk-taking, and socioeconomic circumstances (family structure, household income, highest parental education in household, highest parental occupation in household, and area-level deprivation). Values may not add up due to rounding. Abbreviations: AOR = Adjusted odds ratio; ARRR = Adjusted relative risk ratio; CI = Confidence interval; Hr/s = Hour/s; Min/s = Minute/s; n = Number of participants; OR = Odds ratio; Ref = Reference category, and RRR = Relative risk ratio.

# APPENDIX-H. Differential effect of social media use on cigarette and e-cigarette use by socioeconomic circumstance

## Assessment on the additive scale using risk differences (RDs)

Table-H1. Participant cigarette use according to time spent on social media, within strata of parental education and according to ‘combinations’ of time spent on social media and parental education within the questionnaire and time-use-diary imputed samples (condensed table shown in manuscript)

|  | **Questionnaire imputed sample (n = 8,954)** | | **Time-use-diary imputed sample (n = 2,520)** | |
| --- | --- | --- | --- | --- |
|  | **High parental education** | **Low parental education** | **High parental education** | **Low parental education** |
| **Weighted prevalence % (observed n with outcome/without outcome)** | | | | |
| No social media use | 10.1 (43/391) | 22.1 (49/246) | 20.3 (149/666) | 30.4 (90/273) |
| 1 - <30 mins | 13.0 (95/617) | 23.4 (78/352) | 25.4 (75/279) | 11.7 (19/124) |
| 30 mins - <1 hr | 23.7 (173/651) | 22.8 (99/402) | 26.6 (66/199) | 30.9 (29/96) |
| 1 - <2 hrs | 26.8 (237/701) | 27.0 (154/465) | 25.7 (39/134) | 36.2 (34/68) |
| ≥2 hrs | 36.8 (768/1,484) | 38.6 (677/1,272)) | 31.0 (34/70) | 44.3 (24/51) |
| **Unadjusted RD (95% CI; *p-value*) for time spent on social media within strata of parental education** | | | | |
| No social media use | Ref | Ref | Ref | Ref |
| 1 - <30 mins | 2.9 (-1.9 to 7.8; 0.239) | 1.3 (-12.1 to 14.7; 0.848) | 5.1 (-0.7 to 10.9; 0.082) | -18.8 (-28.7 to -8.8; <0.0001) |
| 30 mins - <1 hr | 13.6 (7.7 to 19.4; <0.0001) | 0.6 (-11.8 to 13.0; 0.921) | 6.3 (-0.5 to 13.1; 0.071) | 0.5 (-16.6 to 17.5; 0.957) |
| 1 - <2 hrs | 16.7 (12.1 to 21.3; <0.0001) | 4.9 (-7.6 to 17.4; 0.442) | 5.4 (-5.2 to 16.0; 0.318) | 5.8 (-8.3 to 19.9; 0.423) |
| ≥2 hrs | 26.7 (22.0 to 31.4; <0.0001) | 16.4 (5.0 to 27.8; 0.005) | 10.7 (-1.1 to 22.5; 0.074) | 13.8 (-4.0 to 31.6; 0.127) |
| **Unadjusted RD (95% CI; *p-value*) for time spent on social media and parental education** | | | | |
| No social media use | Ref | 12.0 (0.4 to 23.7; 0.043) | Ref | 10.1 (2.3 to 18.0; 0.012) |
| 1 - <30 mins | 2.9 (-1.9 to 7.8; 0.239) | 13.3 (5.0 to 21.6; 0.002) | 5.1 (-0.7 to 10.9; 0.082) | -8.6 (-15.9 to -1.4; 0.020) |
| No social media use | Ref | 12.0 (0.4 to 23.7; 0.043) | Ref | 10.1 (2.3 to 18.0; 0.012) |
| 30 mins - <1 hr | 13.6 (7.7 to 19.4; <0.0001) | 12.6 (5.8 t19.5; <0.0001) | 6.3 (-0.5 to 13.1; 0.071) | 10.6 (-4.9 to 26.1; 0.179) |
| No social media use | Ref | 12.0 (0.4 to 23.7; 0.043) | Ref | 10.1 (2.3 to 18.0; 0.012) |
| 1 - <2 hrs | 16.7 (12.1 to 21.3; <0.0001) | 16.9 (10.3 to 23.5; <0.0001) | 5.4 (-5.2 to 16.0; 0.318) | 15.9 (2.7 to 29.1; 0.018) |
| No social media use | Ref | 12.0 (0.4 to 23.7; 0.043) | Ref | 10.1 (2.3 to 18.0; 0.012) |
| ≥2 hrs | 26.7 (22.0 to 31.4; <0.0001) | 28.4 (23.6 to 33.3; <0.0001) | 10.7 (-1.1 to 22.5; 0.074) | 24.0 (7.2 to 40.7; 0.005) |
| **Unadjusted measure of additive effect modification^a^ and interaction^b^ (95% CI; *p-value*)** | | | | |
| No social media use | Ref | | Ref | |
| 1 - <30 mins | -1.6 (-15.8 to 12.6; 0.823) | | -23.9 (-34.6 to -13.1; <0.0001) | |
| 30 mins - <1 hr | -12.9 (-26.3 to 0.5; 0.059) | | -5.8 (-24.2 to 12.6; 0.534) | |
| 1 - <2 hrs | -11.8 (-25.1 to 1.4; 0.081) | | 0.4 (-17.4 to 18.2; 0.967) | |
| ≥2 hrs | -10.3 (-22.4 to 1.9; 0.097) | | 3.1 (-18.0 to 24.2; 0.770) | |
| **Adjusted^c^ RD (95% CI; *p-value*) for time spent on social media within strata of parental education** | | | | |
| No social media use | Ref | Ref | Ref | Ref |
| 1 - <30 mins | 2.0 (-2.6 to 6.6; 0.386) | 2.6 (-8.4 to 13.5; 0.646) | 5.5 (-0.4 to 11.4; 0.067) | -14.9 (-24.7 to -5.2; 0.003) |
| 30 mins - <1 hr | 12.4 (6.9 to 18.0; <0.0001) | 1.4 (-9.2 to 11.9; 0.799) | 6.0 (-0.9 to 13.0; 0.089) | 4.2 (-11.4 to 19.8; 0.598) |
| 1 - <2 hrs | 14.9 (10.5 to 19.3; <0.0001) | 3.4 (-7.5 to 14.3; 0.541) | 4.2 (-5.7 to 14.1; 0.405) | 4.7 (-8.2 to 17.5; 0.476) |
| ≥2 hrs | 24.6 (20.0 to 29.2; <0.0001) | 14.2 (3.9 to 24.5; 0.007) | 10.2 (-1.7 to 22.0; 0.092) | 13.9 (-1.2 to 29.1; 0.072) |
| **Adjusted^c^ RD (95% CI; *p-value*) for time spent on social media and parental education** | | | | |
| No social media use | Ref | 4.6 (-4.9 to 14.2; 0.340) | Ref | 5.4 (-0.9 to 11.7; 0.091) |
| 1 - <30 mins | 2.0 (-2.6 to 6.6; 0.386) | 7.2 (0.3 to 14.1; 0.041) | 5.5 (-0.4 to 11.4; 0.067) | -9.5 (-17.6 to -1.3; 0.023) |
| No social media use | Ref | 6.4 (-3.1 to 15.8; 0.188) | Ref | 3.4 (-2.8 to 9.6; 0.284) |
| 30 mins - <1 hr | 12.4 (6.9 to 18.0; <0.0001) | 7.7 (1.7 to 13.8; 0.013) | 6.0 (-0.9 to 13.0; 0.089) | 7.6 (-7.2 to 22.3; 0.313) |
| No social media use | Ref | 9.5 (-0.3 to 19.3; 0.057) | Ref | 4.0 (-2.3 to 10.4; 0.211) |
| 1 - <2 hrs | 14.9 (10.5 to 19.3; <0.0001) | 12.9 (6.7 to 19.2;<0.0001) | 4.2 (-5.7 to 14.2; 0.405) | 8.7 (-4.6 to 22.0; 0.198) |
| No social media use | Ref | 7.4 (-2.7 to 17.6; 0.149) | Ref | 3.5 (-2.8 to 9.8; 0.276) |
| ≥2 hrs | 24.6 (20.0 to 29.2; <0.0001) | 21.6 (17.1 to 26.1; <0.0001) | 10.2 (-1.7 to 22.0; 0.092) | 17.4 (3.5 to 31.3; 0.014) |
| **Adjusted^c^ measure of additive effect modification^a^ and interaction^b^ (95% CI; *p-value)*** | | | | |
| No social media use | Ref | | Ref | |
| 1 - <30 mins | 0.5 (-11.2 to 12.2; 0.929) | | -20.4 (-31.1 to -9.8; <0.0001) | |
| 30 mins - <1 hr | -11.1 (-22.7 to 0.5; 0.061) | | -1.9 (-18.7 to 15.0; 0.829) | |
| 1 - <2 hrs | -11.6 (-23.0 to -0.1; 0.048) | | 0.5 (-15.3 to 16.2; 0.953) | |
| ≥2 hrs | -10.5 (-21.3 to 0.3; 0.057) | | 3.7 (-14.8 to 22.3; 0.691) | |

**Legend:** Questionnaire imputed sample: n = 8,954 (weighted sample: n = 6,976). Time-use-diary imputed sample: n = 2,520 (weighted sample: n = 5,727). ^a^ Measure of effect modification on an additive scale represents the size of the absolute difference between the RDs for participant current or former cigarette use by time spent on social media, within the low parental education group compared with baseline (high parental education group).^b^ Measure of interaction on an additive scale represents the size of the difference between the RD in participants with: for example, low parental education and 1-<30 mins social media use compared with RD for participants with low parental education and no social media use plus the RD for those with high parental education and 1-<30 mins social media use. ^c^ Adjusted for: ethnicity, sex, parental cigarette use, parental e-cigarette use, parenting style, previous cigarette use, previous e-cigarette use, anti-social behaviour, previous alcohol use, urbanicity, age, number of siblings in household, maternal age at participant birth, in-person activities, cognitive ability, mental health, and risk-taking. Values may not add up due to rounding. Abbreviations: CI = Confidence interval; Hr/s = Hour/s; Min/s = Minute/s; n = Number of participants; RD = Risk differences, and Ref = Reference category.

Table-H2. Participant e-cigarette use according to time spent on social media, within strata of parental education and according to ‘combinations’ of time spent on social media and parental education within the questionnaire and time-use-diary imputed samples (condensed table shown in manuscript)

|  | **Questionnaire imputed sample (n = 8,954)** | | **Time-use-diary imputed sample (n = 2,520)** | |
| --- | --- | --- | --- | --- |
|  | **High parental education** | **Low parental education** | **High parental education** | **Low parental education** |
| **Weighted prevalence % (observed n with outcome/without outcome)** | | | | |
| No social media use | 9.4 (43/391) | 18.0 (42/253) | 16.1 (118/697) | 27.8 (86/277) |
| 1 - <30 mins | 9.6 (75/636) | 20.4 (81/349) | 17.4 (51/303) | 20.9 (22/121) |
| 30 mins - <1 hr | 19.3 (152/672) | 25.3 (109/393) | 21.5 (44/221) | 20.6 (27/98) |
| 1 - <2 hrs | 19.3 (188/751) | 25.1 (160/459) | 13.8 (31/143) | 30.1 (30/72) |
| ≥2 hrs | 27.8 (590/1,662) | 31.7 (607/1,343) | 15.7 (21/83) | 40.3 (25/50) |
| **Unadjusted RD (95% CI; *p-value*) for time spent on social media within strata of parental education** | | | | |
| No social media use | Ref | Ref | Ref | Ref |
| 1 - <30 mins | 0.3 (-4.3 to 4.8; 0.913) | 2.5 (-8.3 to 13.2; 0.652) | 1.3 (-4.8 to 7.5; 0.670) | -6.8 (-22.7 to 9.1; 0.400) |
| 30 mins - <1 hr | 9.9 (4.9 to 14.9; <0.0001) | 7.4 (-3.5 to 18.2; 0.182) | 5.4 (-2.5 to 13.3; 0.182) | -7.2 (-18.2 to 3.8; 0.201) |
| 1 - <2 hrs | 10.0 (5.2 to 14.7; <0.0001) | 7.2 (-3.9 to 18.3; 0.203) | -2.3 (-10.0 to 5.4; 0.559) | 2.4 (-10.5 to 15.3; 0.716) |
| ≥2 hrs | 18.5 (14.2 to 22.8; <0.0001) | 13.8 (3.6 to 23.9; 0.008) | -0.5 (-9.5 to 8.6; 0.922) | 12.6 (-6.0 to 31.1; 0.184) |
| **Unadjusted RD (95% CI; *p-value*) for time spent on social media and parental education** | | | | |
| No social media use | Ref | 8.6 (-1.7 to 18.9; 0.101) | Ref | 11.6 (3.5 to 19.8; 0.005) |
| 1 - <30 mins | 0.3 (-4.3 to 4.8; 0.913) | 11.1 (4.4 to 17.7; 0.001) | 1.3 (-4.8 to 7.5; 0.670) | 4.8 (-9.5 to 19.1; 0.507) |
| No social media use | Ref | 8.6 (-1.7 to 18.9; 0.101) | Ref | 11.6 (3.5 to 19.8; 0.005) |
| 30 mins - <1 hr | 9.9 (4.9 to 14.9; <0.0001) | 16.0 (9.1 to 22.8; <0.0001) | 5.4 (-2.5 to 13.3; 0.182) | 4.5 (-5.6 to 14.5; 0.384) |
| No social media use | Ref | 8.6 (-1.7 to 18.9; 0.101) | Ref | 11.6 (3.5 to 19.8; 0.005) |
| 1 - <2 hrs | 10.0 (5.2 to 14.7; <0.0001) | 15.8 (9.1 to 22.5; <0.0001) | -2.3 (-10.0 to 5.4; 0.559) | 14.0 (3.1 to 25.0; 0.012) |
| No social media use | Ref | 8.6 (-1.7 to 18.9; 0.101) | Ref | 11.6 (3.5 to 19.8; 0.005)) |
| ≥2 hrs | 18.5 (14.2 to 22.8; <0.0001) | 22.4 (17.5 to 27.3; <0.0001) | -0.5 (-9.5 to 8.6; 0.922) | 24.2 (7.0 to 41.4; 0.006) |
| **Unadjusted measure of additive effect modification ^a^ and interaction^b^ (95% CI; *p-value*)** | | | | |
| No social media use | Ref | | Ref | |
| 1 - <30 mins | 2.2 (-9.1 to 13.5; 0.701) | | -8.2 (-25.0 to 8.7; 0.343) | |
| 30 mins - <1 hr | -2.5 (-14.2 to 9.1; 0.669) | | -12.6 (-26.4 to 1.2; 0.074) | |
| 1 - <2 hrs | -2.8 (-14.7 to 9.2; 0.647) | | 4.7 (-11.7 to 21.0; 0.574) | |
| ≥2 hrs | -4.7 (-15.7 to 6.3; 0.399) | | 13.0 (-8.2 to 34.2; 0.228) | |
| **Adjusted^c^ RD (95% CI; *p-value*) for time spent on social media within strata of parental education** | | | | |
| No social media use | Ref | Ref | Ref | Ref |
| 1 - <30 mins | -0.6 (-5.0 to 3.7; 0.772) | 3.0 (-5.8 to 11.9; 0.501) | 2.6 (-3.7 to 8.9; 0.417) | -4.6 (-17.2 to 8.1; 0.477) |
| 30 mins - <1 hr | 9.6 (4.6 to 14.6; <0.0001) | 9.8 (0.4 to 19.3; 0.041) | 7.2 (-1.0 to 15.5; 0.085) | -0.4 (-10.5 to 9.7; 0.935) |
| 1 - <2 hrs | 10.4 (5.6 to 15.2; <0.0001) | 8.1 (-1.6 to 17.9; 0.101) | -1.0 (-9.2 to 7.1; 0.804) | 3.4 (-8.1 to 15.0; 0.556) |
| ≥2 hrs | 21.6 (17.4 to 25.8; <0.0001) | 18.0 (9.0 to 27.0; <0.0001) | 1.9 (-7.5 to 11.3; 0.689) | 13.2 (-3.4 to 29.7; 0.118) |
| **Adjusted^c^ RD (95% CI; *p-value*) for time spent on social media and parental education** | | | | |
| No social media use | Ref | 3.0 (-5.5 to 11.4; 0.490) | Ref | 7.7 (0.6 to 14.8; 0.035) |
| 1 - <30 mins | -0.6 (-5.0 to 3.7; 0.771) | 6.0 (0.1 to 11.9; 0.048) | 2.6 (-3.7 to 8.9; 0.417) | 3.1 (-8.3 to 14.4; 0.593) |
| No social media use | Ref | 2.3 (-5.8 to 10.4; 0.578) | Ref | 6.6 (-0.4 (13.7; 0.063) |
| 30 mins - <1 hr | 9.6 (4.6 to 14.6; <0.0001) | 12.1 (5.8 to 18.5; <0.0001) | 7.2 (-1.0 to 15.5; 0.085) | 6.2 (-3.7 to 16.1; 0.216) |
| No social media use | Ref | 4.3 (-4.4 to 12.9; 0.331) | Ref | 7.3 (0.3 to 14.3; 0.041) |
| 1 - <2 hrs | 10.4 (5.6 to 15.2; <0.0001) | 12.4 (6.4 to 18.4; <0.0001) | -1.0 (-9.2 to 7.1; 0.804) | 10.7 (0.1 to 21.4; 0.049) |
| No social media use | Ref | 3.1 (-5.6 to 11.7; 0.487) | Ref | 6.4 (-0.6 to 13.3; 0.072) |
| ≥2 hrs | 21.6 (17.4 to 25.8; <0.0001) | 21.1 (16.3 to 25.9; <0.0001) | 1.9 (-7.5 to 11.3; 0.689) | 19.5 (4.3 to 34.8; 0.012) |
| **Adjusted^c^ measure of additive effect modification^a^ and interaction^b^ (95% CI; *p-value*)** | | | |  |
| No social media use | Ref | | Ref | |
| 1 - <30 mins | 3.7 (-5.7 to 13.0; 0.441) | | -7.2 (-21.3 to 7.0; 0.319) | |
| 30 mins - <1 hr | 0.2 (-9.8 to 10.3; 0.965) | | -7.6 (-21.0 to 5.7; 0.260) | |
| 1 - <2 hrs | -2.3 (-12.4 to 7.9; 0.662) | | 4.5 (-10.0 to 19.0; 0.543) | |
| ≥2 hrs | -3.6 (-13.2 to 6.1; 0.463) | | 11.3 (-7.9 to 30.4; 0.248) | |

**Legend:** Questionnaire imputed sample: n = 8,954 (weighted sample: n = 6,976). Time-use-diary imputed sample: n = 2,520 (weighted sample: n = 5,727). ^a^ Measure of effect modification on an additive scale represents the size of the absolute difference between the RDs for participant current or former e-cigarette use by time spent on social media, within the low parental education group compared with baseline (high parental education group).^b^ Measure of interaction on an additive scale represents the size of the difference between the RD in participants with: for example, low parental education and 1-<30 mins social media use compared with RD for participants with low parental education and no social media use plus the RD for those with high parental education and 1-<30 mins social media use. ^c^ Adjusted for: ethnicity, sex, parental cigarette use, parental e-cigarette use, parenting style, previous cigarette use, previous e-cigarette use, anti-social behaviour, previous alcohol use, urbanicity, age, number of siblings in household, maternal age at participant birth, in-person activities, cognitive ability, mental health, and risk-taking. Values may not add up due to rounding. Abbreviations: CI = Confidence interval; Hr/s = Hour/s; Min/s = Minute/s; n = Number of participants; RD = Risk differences, and Ref = Reference category.

Table-H3. Participant cigarette use according to time spent on social media, within strata of parental education and according to ‘combinations’ of time spent on social media and parental education within the questionnaire and time-use-diary complete case samples

|  | **Questionnaire complete case sample (n = 6,234)** | | **Time-use-diary complete case sample (n = 2,109)** | |
| --- | --- | --- | --- | --- |
|  | **High parental education** | **Low parental education** | **High parental education** | **Low parental education** |
| **Weighted prevalence % (observed n with outcome/without outcome)** | | | | |
| No social media use | 13.0 (36/273) | 18.8 (26/121) | 20.3 (126/575) | 31.6 (76/201) |
| 1 - <30 mins | 14.2 (77/467) | 21.0 (45/209) | 25.5 (65/243) | 11.4 (13/95) |
| 30 mins - <1 hr | 23.9 (136/490) | 27.0 (78/235) | 24.0 (54/176) | 21.7 (20/81) |
| 1 - <2 hrs | 26.5 (178/522) | 26.8 (108/279) | 29.0 (37/112) | 40.1 (29/53) |
| ≥2 hrs | 37.7 (586/1,095) | 39.4 (460/813) | 33.3 (31/57) | 49.9 (24/41) |
| **Unadjusted RD (95% CI; *p-value*) for time spent on social media within strata of parental education** | | | | |
| No social media use | Ref | Ref | Ref | Ref |
| 1 - <30 mins | 1.2 (-4.9 to 7.4; 0.694) | 2.2 (-8.0 to 12.5; 0.667) | 5.2 (-1.1 to 11.5; 0.108) | -20.2 (-31.3 to -9.1; <0.0001) |
| 30 mins - <1 hr | 10.9 (3.5 to 18.2; 0.004) | 8.3 (-1.1 to 17.6; 0.082) | 3.7 (-3.7 to 11.1; 0.327) | -9.9 (-23.5 to 3.7; 0.153) |
| 1 - <2 hrs | 13.6 (7.4 to 19.7; <0.0001) | 8.1 (-1.8 to 18.0; 0.111) | 8.7 (-3.6 to 20.9; 0.164) | 8.4 (-6.2 to 23.1; 0.258) |
| ≥2 hrs | 24.8 (18.5 to 31.0; <0.0001) | 20.7 (12.1 to 29.3; <0.0001) | 13.0 (0.6 to 25.4; 0.041) | 18.2 (-1.0 to 37.5; 0.063) |
| **Unadjusted RD (95% CI; *p-value*) for time spent on social media and parental education** | | | | |
| No social media use | Ref | 5.8 (-3.3 to 14.8; 0.211) | Ref | 11.3 (3.1 to 19.5; 0.007) |
| 1 - <30 mins | 1.2 (-4.9 to 7.4; 0.694) | 8.0 (-0.4 to 16.4; 0.061) | 5.2 (-1.1 to 11.5; 0.108) | -8.9 )-17.6 to -0.3; 0.042) |
| No social media use | Ref | 5.8 (-3.3 to 14.8; 0.211) | Ref | 11.3 (3.1 to 19.5; 0.007) |
| 30 mins - <1 hr | 10.9 (3.5 to 18.2; 0.004) | 14.1 (6.5 to 21.6; <0.0001) | 3.7 (-3.7 to 11.1; 0.327) | 1.4 (-10.8 to 13.6; 0.824) |
| No social media use | Ref | 5.8 (-3.3 to 14.8; 0.211) | Ref | 11.3 (3.1 to 19.5; 0.007) |
| 1 - <2 hrs | 13.6 (7.4 to 19.7; <0.0001) | 13.8 (5.9 to 21.8; 0.001) | 8.7 (-3.6 to 20.9; 0.164) | 19.7 (4.3 to 35.2; 0.012) |
| No social media use | Ref | 5.8 (-3.3 to 14.8; 0.210) | Ref | 11.3 (3.1 to 19.5; 0.007) |
| ≥2 hrs | 24.8 (18.5 to 31.0; <0.0001) | 26.5 (20.2 to 32.8; <0.0001) | 13.0 (0.6 to 25.4; 0.041) | 29.5 (11.2 to 47.8; 0.002) |
| **Unadjusted measure of additive effect modification^a^ and interaction^b^ (95% CI; *p-value*)** | | | | |
| No social media use | Ref | | Ref | |
| 1 - <30 mins | 1.0 (-10.5 to 12.6; 0.864) | | -25.4 (-37.9 to -12.8; <0.0001) | |
| 30 mins - <1 hr | -2.6 (-14.1 to 8.9; 0.656) | | -13.6 (-29.2 to 2.0; 0.087) | |
| 1 - <2 hrs | -5.5 (-17.4 to 6.4; 0.365) | | -0.2 (-19.5 to 19.0; 0.982) | |
| ≥2 hrs | -4.0 (-14.5 to 6.5; 0.451) | | 5.2 (-18.0 to 28.4; 0.659) | |
| **Adjusted^c^ RD (95% CI; *p-value*) for time spent on social media within strata of parental education** | | | | |
| No social media use | Ref | Ref | Ref | Ref |
| 1 - <30 mins | 0.1 (-5.2 to 5.5; 0.965) | 4.2 (-5.2 to 13.7; 0.381) | 4.8 (-1.6 to 11.3; 0.143) | -16.6 (-27.3 to -5.9; 0.002) |
| 30 mins - <1 hr | 9.9 (3.4 to 16.5; 0.003) | 9.4 (0.3 to 18.4; 0.042) | 3.6 (-4.0 to 11.2; 0.355) | -5.8 (-18.4 to 6.9; 0.369) |
| 1 - <2 hrs | 12.2 (6.6 to 17.7; <0.0001) | 8.3 (-1.1 to 17.8; 0.084) | 4.3 (-7.3 to 16.0; 0.465) | 3.1 (-11.7 to 18.0; 0.678) |
| ≥2 hrs | 22.5 (16.4 to 28.5; <0.0001) | 17.7 (8.8 to 26.7; <0.0001) | 11.6 (-1.6 to 24.7; 0.084) | 18.0 (1.1 to 35.0; 0.037) |
| **Adjusted^c^ RD (95% CI; *p-value*) for time spent on social media and parental education** | | | | |
| No social media use | Ref | -1.1 (-9.6 to 7.4; 0.796) | Ref | 7.2 (-0.3 to 14.7; 0.060) |
| 1 - <30 mins | 0.1 (-5.2 to 5.5; 0.965) | 3.1 (-3.8 to 10.0; 0.377) | 4.8 (-1.6 to 11.3; 0.143) | -9.4 (-18.5 to -0.4; 0.041) |
| No social media use | Ref | -0.9 (-9.5 to 7.7; 0.833) | Ref | 5.5 (-1.9 to 12.9; 0.143) |
| 30 mins - <1 hr | 9.9 (3.4 to 16.5; 0.003) | 8.5 (1.1 to 15.8; 0.024) | 3.6 (-4.0 to 11.2; 0.355) | -0.3 (-11.9 to 11.4; 0.965) |
| No social media use | Ref | 2.5 (-6.2 to 11.2; 0.576) | Ref | 5.9 (-1.7 to 13.5; 0.125) |
| 1 - <2 hrs | 12.2 (6.6 to 17.7; <0.0001) | 10.8 (3.3 to 18.3; 0.005) | 4.3 (-7.3 to 16.0; 0.465) | 9.1 (-7.0 to 25.1; 0.267) |
| No social media use | Ref | 2.4 (-6.2 to 11.0; 0.585) | Ref | 5.3 (-2.2 to 12.9; 0.163) |
| ≥2 hrs | 22.5 (16.4 to 28.5; <0.0001) | 20.1 (14.0 to 26.; <0.0001) | 11.6 (-1.6 to 24.7; 0.084) | 23.4 (7.8 to 39.0; 0.003) |
| **Adjusted^c^ measure of additive effect modification^a^ and interaction^b^ (95% CI; *p-value*)** | | | | |
| No social media use | Ref | | Ref | |
| 1 - <30 mins | 4.1 (-6.3 to 14.5; 0.439) | | -21.4 (-33.9 to -9.0; 0.001) | |
| 30 mins - <1 hr | -0.5 (-11.2 to 10.1; 0.921) | | -9.4 (-23.7 to 4.9; 0.196) | |
| 1 - <2 hrs | -3.8 (-14.6 to 6.9; 0.483) | | -1.2 (-19.0 to 16.6; 0.894) | |
| ≥2 hrs | -4.7 (-14.4 to 4.9; 0.332) | | 6.4 (-14.8 to 27.7; 0.550) | |

**Legend:** Questionnaire complete case sample: n = 6,234 (weighted sample: n = 4,851). Time-use-diary complete case sample: n = 2,109 (weighted sample: n = 4,590). ^a^ Measure of effect modification on an additive scale represents the size of the absolute difference between the RDs for participant current or former cigarette use by time spent on social media, within the low parental education group compared with baseline (high parental education group).^b^ Measure of interaction on an additive scale represents the size of the difference between the RD in participants with: for example, low parental education and 1-<30 mins social media use compared with RD for participants with low parental education and no social media use plus the RD for those with high parental education and 1-<30 mins social media use. ^c^ Adjusted for: ethnicity, sex, parental cigarette use, parental e-cigarette use, parenting style, previous cigarette use, previous e-cigarette use, anti-social behaviour, previous alcohol use, urbanicity, age, number of siblings in household, maternal age at participant birth, in-person activities, cognitive ability, mental health, and risk-taking. Values may not add up due to rounding. Abbreviations: CI = Confidence interval; Hr/s = Hour/s; Min/s = Minute/s; n = Number of participants; RD = Risk differences, and Ref = Reference category.

Table-H4. Participant e-cigarette use according to time spent on social media, within strata of parental education and according to ‘combinations’ of time spent on social media and parental education within the questionnaire and time-use-diary complete case samples

|  | **Questionnaire complete case sample (n = 6,234)** | | **Time-use-diary complete case sample (n = 2,109)** | |
| --- | --- | --- | --- | --- |
|  | **High parental education** | **Low parental education** | **High parental education** | **Low parental education** |
| **Weighted prevalence % (observed n with outcome/without outcome)** | | | | |
| No social media use | 9.8 (28/281) | 12.2 (21/126) | 15.1 (92/609) | 29.7 (72/205) |
| 1 - <30 mins | 9.0 (55/489) | 19.2 (46/208) | 16.1 (42/266) | 20.0 (14/94) |
| 30 mins - <1 hr | 18.6 (115/511) | 27.0 (71/242) | 19.3 (36/194) | 21.6 (21/80) |
| 1 - <2 hrs | 18.2 (138/562) | 21.7 (99/288) | 12.9 (26/123) | 32.5 (25/57) |
| ≥2 hrs | 27.2 (434/1,247) | 29.6 (382/891) | 15.8 (18/70) | 39.0 (21/44) |
| **Unadjusted RD (95% CI; *p-value*) for time spent on social media within strata of parental education** | | | | |
| No social media use | Ref | Ref | Ref | Ref |
| 1 - <30 mins | -0.8 (-6.1 to 4.5; 0.770) | 6.9 (-1.4 to 15.2; 0.101) | 1.0 (-5.5 to 7.5; 0.759) | -9.7 (-29.3 to 9.9; 0.330) |
| 30 min - <1 hr | 8.9 (2.7 to 15.0; 0.005) | 14.8 (5.9 to 23.7; 0.001) | 4.3 (-4.1 to 12.6; 0.315) | -8.1 (-20.9 to 4.6; 0.210) |
| 1 - <2 hrs | 8.5 (2.6 to 14.4; 0.005) | 9.5 (1.3 to 17.7; 0.023) | -2.2 (-9.8 to 5.5; 0.581) | 2.7 (-11.2 to 16.7; 0.699) |
| ≥2 hrs | 17.4 (12.1 to 22.7; <0.0001) | 17.4 (10.3 to 24.5; <0.0001) | 0.7 (-8.1 to 9.4; 0.879) | 9.2 (-11.7 to 30.1; 0.387) |
| **Unadjusted RD (95% CI; *p-value*) for time spent on social media and parental education** | | | | |
| No social media use | Ref | 2.5 (-5.3 to 10.3; 0.535) | Ref | 14.7 (6.6 to 22.7; <0.0001) |
| 1 - <30 mins | -0.8 (-6.1 to 4.5; 0.770) | 9.4 (1.9 to 16.9; 0.014) | 1.0 (-5.5 to 7.5; 0.759) | 4.9 (-13.4 to 23.2; 0.596) |
| No social media use | Ref | 2.5 (-5.3 to 10.3; 0.535) | Ref | 14.7 (6.6 to 22.7; <0.0001) |
| 30 mins - <1 hr | 8.9 (2.7 to 15.0; 0.005) | 17.3 (9.0 to 25.6; <0.0001) | 4.3 (-4.1 to 12.6; 0.315) | 6.5 (-5.5 to 18.5; 0.289) |
| No social media use | Ref | 2.5 (-5.3 to 10.3; 0.535) | Ref | 14.7 (6.6 to 22.7; <0.0001) |
| 1 - <2 hrs | 8.5 (2.6 to 14.4; 0.005) | 12.0 (4.9 to 19.1; 0.001) | -2.2 (-9.8 to 5.5; 0.581) | 17.4 (5.0 to 29.8; 0.006) |
| No social media use | Ref | 2.5 (-5.3 to 10.3; 0.534) | Ref | 14.7 (6.6 to 22.7; <0.0001) |
| ≥2 hrs | 17.4 (12.1 to 22.7; <0.0001) | 19.8 (13.8 to 25.9; <0.0001) | 0.7 (-8.1 to 9.4; 0.879) | 23.9 (4.3 to 43.4; 0.017) |
| **Unadjusted measure of additive effect modification^a^ and interaction^b^ (95% CI; *p-value*)** | | | | |
| No social media use | Ref | | Ref | |
| 1 - <30 mins | 7.7 (-1.8 to 17.2; 0.110) | | -10.7 (-31.2 to 9.7; 0.303) | |
| 30 mins - <1 hr | 6.0 (-4.6 to 16.5; 0.267) | | -12.4 (-27.5 to 2.7; 0.107) | |
| 1 - <2 hrs | 1.0 (-8.8 to 10.8; 0.836) | | 4.9 (-12.6 to 22.4; 0.583) | |
| ≥2 hrs | -0.0 (-8.8 to 8.7; 0.992) | | 8.5 (-15.1 to 32.1; 0.477) | |
| **Adjusted^c^ RD (95% CI; *p-value*) for time spent on social media within strata of parental education** | | | | |
| No social media use | Ref | Ref | Ref | Ref |
| 1 - <30 mins | -1.3 (-5.9 to 3.3; 0.582) | 8.2 (0.6 to 15.8; 0.035) | 2.1 (-4.4 to 8.5; 0.531) | -9.6 (-23.0 to 3.8; 0.160) |
| 30 mins - <1 hr | 9.6 (4.0 to 15.3; 0.001) | 17.1 (8.8 to 25.3; <0.0001) | 6.7 (-1.9 to 15.3; 0.127) | -4.5 (-16.3 to 7.3; 0.451) |
| 1 - <2 hrs | 9.6 (3.8 to 15.3; 0.001) | 11.7 (3.7 to 19.7; 0.004) | -1.2 (-9.2 to 6.8; 0.774) | -1.0 (13.3 to 11.3; 0.871) |
| ≥2 hrs | 20.6 (15.9 to 25.3; <0.0001) | 21.2 (14.1 to 28.3; <0.0001) | 0.8 (-9.4 to 11.0; 0.878) | 8.0 (-11.0 to 26.8; 0.410) |
| **Adjusted^c^ RD (95% CI; *p-value*) for time spent on social media and parental education** | | | | |
| No social media use | Ref | -1.5 (-8.8 to 5.8; 0.682) | Ref | 11.0 (3.3 to 18.8; 0.005) |
| 1 - <30 mins | -1.3 (-5.9 to 3.3; 0.582) | 6.7 (0.2 to 13.1; 0.042) | 2.1 (-4.4 to 8.5; 0.531) | 1.4 (-10.4 to 13.3; 0.811) |
| No social media use | Ref | -3.4 (-10.4 to 3.6; 0.341) | Ref | 11.0 (3.3 to 18.6; 0.005) |
| 30 mins - <1 hr | 9.6 (4.0 to 15.3; 0.001) | 13.7 (5.7 to 21.7; 0.001) | 6.7 (-1.9 to 15.3; 0.127) | 6.4 (-5.0 to 17.9; 0.268) |
| No social media use | Ref | -1.3 (-8.6 to 6.1; 0.737) | Ref | 11.3 (3.6 to 19.0; 0.004) |
| 1 - <2 hrs | 9.6 (3.8 to 15.3; 0.001) | 10.4 (3.5 to 17.3; 0.003) | -1.2 (-9.2 to 6.8; 0.774) | 10.3 (-1.7 to 22.3; 0.091) |
| No social media use | Ref | -1.2 (-8.2 to 5.8; 0.732) | Ref | 9.6 (2.1 to 17.2; 0.013) |
| ≥2 hrs | 20.6 (15.9 to 25.3; <0.0001) | 20.0 (14.2 to 25.7; <0.0001) | 0.8 (-9.4 to 11.0; 0.878) | 17.6 (-0.1 to 35.2; 0.051) |
| **Adjusted^c^ measure of additive effect modification^a^ and interaction^b^ (95% CI; *p-value*)** | | | |  |
| No social media use | Ref | | Ref | |
| 1 - <30 mins | 9.5 (0.8 to 18.2; 0.033) | | -11.6 (-26.4 to 3.1; 0.122) | |
| 30 mins - <1 hr | 7.4 (-2.0 to 16.8; 0.120) | | -11.2 (-25.3 to 2.8; 0.117) | |
| 1 - <2 hrs | 2.1 (-7.0 to 11.2; 0.648) | | 0.2 (-14.5 to 14.9; 0.983) | |
| ≥2 hrs | 0.6 (-7.2 to 8.5; 0.877) | | 7.1 (-14.4 to 28.7; 0.516) | |

**Legend:** Questionnaire complete case sample: n = 6,234 (weighted sample: n = 4,851). Time-use-diary complete case sample: n = 2,109 (weighted sample: n = 4,590). ^a^ Measure of effect modification on an additive scale represents the size of the absolute difference between the RDs for participant current or former e-cigarette use by time spent on social media, within the low parental education group compared with baseline (high parental education group).^b^ Measure of interaction on an additive scale represents the size of the difference between the RD in participants with: for example, low parental education and 1-<30 mins social media use compared with RD for participants with low parental education and no social media use plus the RD for those with high parental education and 1-<30 mins social media use. ^c^ Adjusted for: ethnicity, sex, parental cigarette use, parental e-cigarette use, parenting style, previous cigarette use, previous e-cigarette use, anti-social behaviour, previous alcohol use, urbanicity, age, number of siblings in household, maternal age at participant birth, in-person activities, cognitive ability, mental health, and risk-taking. Values may not add up due to rounding. Abbreviations: CI = Confidence interval; Hr/s = Hour/s; Min/s = Minute/s; n = Number of participants; RD = Risk differences, and Ref = Reference category

## Assessment on the multiplicative scale using risk ratios (RRs)

Table-H5. Participant cigarette use according to time spent on social media, within strata of parental education and according to ‘combinations’ of time spent on social media and parental education within the questionnaire and time-use-diary imputed samples

|  | **Questionnaire imputed sample (n = 8,954)** | | **Time-use-diary imputed sample (n = 2,520)** | |
| --- | --- | --- | --- | --- |
|  | **High parental education** | **Low parental education** | **High parental education** | **Low parental education** |
| **Weighted prevalence % (observed n with outcome/without outcome)** | | | | |
| No social media use | 10.1 (43/391) | 22.1 (49/246) | 20.3 (149/666) | 30.4 (90/273) |
| 1 - <30 mins | 13.0 (95/617) | 23.4 (78/352) | 25.4 (75/279) | 11.7 (19/124) |
| 30 mins - <1 hr | 23.7 (173/651) | 22.8 (99/402) | 26.6 (66/199) | 30.9 (29/96) |
| 1 - <2 hrs | 26.8 (237/701) | 27.0 (154/465) | 25.7 (39/134) | 36.2 (34/68) |
| ≥2 hrs | 36.8 (768/1,484) | 38.6 (677/1,272)) | 31.0 (34/70) | 44.3 (24/51) |
| **Unadjusted RR (95% CI; *p-value*) for time spent on social media within strata of parental education** | | | | |
| No social media use | Ref | Ref | Ref | Ref |
| 1 - <30 mins | 1.29 (0.83 to 2.00; 0.262) | 1.06 (0.59 to 1.93; 0.835) | 1.25 (0.98 to 1.60; 0.072) | 0.38 (0.21 to 0.70; 0.002) |
| 30 mins - <1 hr | 2.34 (1.54 to 3.54; <0.0001) | 1.03 (0.59 to 1.80; 0.908) | 1.31 (1.00 to 1.72; 0.054) | 1.02 (0.58 to 1.77; 0.957) |
| 1 - <2 hrs | 2.65 (1.83 to 3.82; <0.0001) | 1.23 (0.71 to 2.12; 0.459) | 1.27 (0.84 to 1.91; 0.263) | 1.19 (0.79 to 1.79; 0.402) |
| ≥2 hrs | 3.63 (2.52 to 5.25; <0.0001) | 1.75 (1.06 to 2.90; 0.029) | 1.53 (1.02 to 2.29; 0.042) | 1.45 (0.94 to 2.25; 0.092) |
| **Unadjusted RR (95% CI; *p-value*) for time spent on social media and parental education** | | | | |
| No social media use | Ref | 2.17 (1.17 to 4.03; 0.014) | Ref | 1.50 (1.13 to 1.99; 0.006) |
| 1 - <30 mins | 1.29 (0.83 to 2.00; 0.262) | 2.31 (1.42 to 3.77; 0.001) | 1.25 (0.98 to 1.60; 0.072) | 0.57 (0.33 to 1.01; 0.056) |
| No social media use | Ref | 2.17 (1.17 to 4.02; 0.014) | Ref | 1.50 (1.13 to 1.99; 0.006) |
| 30 mins - <1 hr | 2.34 (1.54 to 3.54; <0.0001) | 2.42 (1.44 to 3.49; <0.0001) | 1.31 (1.00 to 1.72; 0.054) | 1.52 (0.91 to 2.55; 0.111) |
| No social media use | Ref | 2.17 (1.17 to 4.02; 0.014) | Ref | 1.50 (1.13 to 1.99; 0.006) |
| 1 - <2 hrs | 2.65 (1.83 to 3.82; <0.0001) | 2.67 (1.76 to 4.05; <0.0001) | 1.27 (0.84 to 1.91; 0.263) | 1.78 (1.21 to 2.62; 0.003) |
| No social media use | Ref | 2.17 (1.17 to 4.02; 0.014) | Ref | 1.50 (1.13 to 1.99; 0.006) |
| ≥2 hrs | 3.63 (2.52 to 5.25; <0.0001) | 3.81 (2.63 to 5.51; <0.0001) | 1.53 (1.02 to 2.29; 0.042) | 2.18 (1.45 to 3.28; <0.0001) |
| **Unadjusted measure of multiplicative effect modification and interaction (95% CI; *p-value*)** | | | | |
| No social media use | Ref | | Ref | |
| 1 - <30 mins | 0.83 (0.40 to 1.73; 0.612) | | 0.31 (0.16 to 0.57; <0.0001) | |
| 30 mins - <1 hr | 0.44 (0.22 to 0.87; 0.019) | | 0.78 (0.42 to 1.44; 0.418) | |
| 1 - <2 hrs | 0.46 (0.24 to 0.89; 0.021) | | 0.94 (0.52 to 1.69; 0.836) | |
| ≥2 hrs | 0.48 (0.26 to 0.90; 0.021) | | 0.95 (0.53 to 1.72; 0.874) | |
| **Adjusted^a^ RR (95% CI; *p-value*) for time spent on social media within strata of parental education** | | | | |
| No social media use | Ref | Ref | Ref | Ref |
| 1 - <30 mins | 1.28 (0.85 to 1.93; 0.240) | 1.10 (0.67 to 1.81; 0.699) | 1.27 (0.99 to 1.63; 0.061) | 0.44 (0.24 to 0.82; 0.010) |
| 30 mins - <1 hr | 2.30 (1.56 to 3.38; <0.0001) | 1.04 (0.64 to 1.69; 0.877) | 1.30 (0.99 to 1.72; 0.063) | 1.18 (0.70 to 1.98; 0.536) |
| 1 - <2 hrs | 2.52 (1.80 to 3.52; <0.0001) | 1.13 (0.70 to 1.83; 0.616) | 1.22 (0.83 to 1.80; 0.309) | 1.13 (0.77 to 1.66; 0.519) |
| ≥2 hrs | 3.47 (2.48 to 4.87; <0.0001) | 1.64 (1.03 to 2.62; 0.037) | 1.51 (0.99 to 2.30; 0.056) | 1.42 (0.97 to 2.10; 0.074) |
| **Adjusted^a^ RR (95% CI; *p-value*) for time spent on social media and parental education** | | | | |
| No social media use | Ref | 1.49 (0.87 to 2.56; 0.150) | Ref | 1.25 (0.97 to 1.60; 0.083) |
| 1 - <30 mins | 1.28 (0.85 to 1.93; 0.240) | 1.64 (1.07 to 2.52; 0.025) | 1.27 (0.99 to 1.63; 0.061) | 0.55 (0.30 to 1.00; 0.051) |
| No social media use | Ref | 1.74 (1.03 to 2.95; 0.039) | Ref | 1.16 (0.90 to 1.49; 0.256) |
| 30 mins - <1 hr | 2.30 (1.56 to 3.38; <0.0001) | 1.81 (1.21 to 2.71; 0.004) | 1.30 (0.99 to 1.71; 0.063) | 1.36 (0.82 to 2.27; 0.235) |
| No social media use | Ref | 2.02 (1.19 to 3.43; 0.009) | Ref | 1.20 (0.93 to 1.56; 0.165) |
| 1 - <2 hrs | 2.52 (1.80 to 3.52; <0.0001) | 2.28 (1.54 to 3.38; <0.0001) | 1.22 (0.83 to 1.80; 0.309) | 1.36 (0.91 to 2.05; 0.136) |
| No social media use | Ref | 1.93 (1.10 to 3.37; 0.022) | Ref | 1.17 (0.91 to 1.51; 0.223) |
| ≥2 hrs | 3.47 (2.48 to 4.87; <0.0001) | 3.16 (2.26 to 4.43; <0.0001) | 1.51 (0.99 to 2.30; 0.056) | 1.67 (1.17 to 2.39; 0.005) |
| **Adjusted^a^ measure of multiplicative effect modification and interaction (95% CI; *p-value*)** | | | | |
| No social media use | Ref | | Ref | |
| 1 - <30 mins | 0.86 (0.46 to 1.62; 0.645) | | 0.35 (0.18 to 0.65; 0.001) | |
| 30 mins - <1 hr | 0.45 (0.25 to 0.83; 0.010) | | 0.91 (0.51 to 1.61; 0.735) | |
| 1 - <2 hrs | 0.45 (0.26 to 0.79; 0.006) | | 0.93 (0.55 to 1.56; 0.774) | |
| ≥2 hrs | 0.47 (0.27 to 0.84; 0.010) | | 0.94 (0.55 to 1.63; 0.834) | |

**Legend:** Questionnaire imputed sample: n = 8,954 (weighted sample: n = 6,976). Time-use-diary imputed sample: n = 2,520 (weighted sample: n = 5,727). ^a^ Adjusted for: ethnicity, sex, parental cigarette use, parental e-cigarette use, parenting style, previous cigarette use, previous e-cigarette use, anti-social behaviour, previous alcohol use, urbanicity, age, number of siblings in household, maternal age at participant birth, in-person activities, cognitive ability, mental health, and risk-taking. Values may not add up due to rounding. Abbreviations: CI = Confidence interval; Hr/s = Hour/s; Min/s = Minute/s; n = Number of participants; Ref = Reference category, and RR = Risk ratio.

Table-H6. Participant e-cigarette use according to time spent on social media, within strata of parental education and according to ‘combinations’ of time spent on social media and parental education within the questionnaire and time-use-diary imputed samples

|  | **Questionnaire imputed sample (n = 8,954)** | | **Time-use-diary imputed sample (n = 2,520)** | |
| --- | --- | --- | --- | --- |
|  | **High parental education** | **Low parental education** | **High parental education** | **Low parental education** |
| **Weighted prevalence % (observed n with outcome/without outcome)** | | | | |
| No social media use | 9.4 (43/391) | 18.0 (42/253) | 16.1 (118/697) | 27.8 (86/277) |
| 1 - <30 mins | 9.6 (75/636) | 20.4 (81/349) | 17.4 (51/303) | 20.9 (22/121) |
| 30 mins - <1 hr | 19.3 (152/672) | 25.3 (109/393) | 21.5 (44/221) | 20.6 (27/98) |
| 1 - <2 hrs | 19.3 (188/751) | 25.1 (160/459) | 13.8 (31/143) | 30.1 (30/72) |
| ≥2 hrs | 27.8 (590/1,662) | 31.7 (607/1,343) | 15.7 (21/83) | 40.3 (25/50) |
| **Unadjusted RR (95% CI; *p-value*) for time spent on social media within strata of parental education** | | | | |
| No social media use | Ref | Ref | Ref | Ref |
| 1 - <30 mins | 1.03 (0.63 to 1.67; 0.913) | 1.15 (0.65 to 2.04; 0.633) | 1.08 (0.76 to 1.55; 0.665) | 0.75 (0.37 to 1.56; 0.445) |
| 30 mins - <1 hr | 2.06 (1.33 to 3.19; 0.001) | 1.43 (0.82 to 2.48; 0.209) | 1.33 (0.89 to 1.99; 0.159) | 0.74 (0.45 to 1.21; 0.233) |
| 1 - <2 hrs | 2.06 (1.35 to 3.15; 0.001) | 1.42 (0.80 to 2.50; 0.230) | 0.86 (0.50 to 1.47; 0.577) | 1.09 (0.70 to 1.69; 0.713) |
| ≥2 hrs | 2.98 (2.00 to 4.43; <0.0001) | 1.79 (1.05 to 3.05; 0.034) | 0.97 (0.55 to 1.72; 0.913) | 1.45 (0.88 to 2.40; 0.144) |
| **Unadjusted RR (95% CI; *p-value*) for time spent on social media and parental education** | | | | |
| No social media use | Ref | 1.90 (0.99 to 3.65; 0.054) | Ref | 1.72 (1.21 to 2.45; 0.003) |
| 1 - <30 mins | 1.03 (0.63 to 1.67; 0.913) | 2.18 (1.34 to 3.56; 0.002) | 1.08 (0.76 to 1.55; 0.665) | 1.30 (0.65 to 2.60; 0.458) |
| No social media use | Ref | 1.90 (0.99 to 3.65; 0.054) | Ref | 1.72 (1.21 to 2.45; 0.003) |
| 30 mins - <1 hr | 2.06 (1.33 to 3.19; 0.001) | 2.71 (1.71 to 4.28; <0.0001) | 1.33 (0.89 to 1.99; 0.159) | 1.28 (0.77 to 2.12; 0.346) |
| No social media use | Ref | 1.90 (0.99 to 3.65; 0.054) | Ref | 1.72 (1.21 to 2.45l 0.003) |
| 1 - <2 hrs | 2.06 (1.35 to 3.15; 0.001) | 2.69 (1.69 to 4.27; <0.0001) | 0.86 (0.50 to 1.47; 0.577) | 1.87 (1.25 to 2.80; 0.002) |
| No social media use | Ref | 1.90 (0.99 to 3.65; 0.054) | Ref | 1.72 (1.21 to 2.45; 0.003) |
| ≥2 hrs | 2.98 (2.00 to 4.43; <0.0001) | 3.39 (2.25 to 5.12; <0.0001) | 0.97 (0.55 to 1.72; 0.913) | 2.50 (1.55 to 4.04; <0.0001) |
| **Unadjusted measure of multiplicative effect modification and interaction (95% CI; *p-value*)** | | | | |
| No social media use | Ref | | Ref | |
| 1 - <30 mins | 1.12 (0.54 to 2.29; 0.760) | | 0.70 (0.31 to 1.55; 0.375) | |
| 30 mins - <1 hr | 0.69 (0.35 to 1.38; 0.295) | | 0.55 (0.29 to 1.06; 0.075) | |
| 1 - <2 hrs | 0.69 (0.34 to 1.38; 0.286) | | 1.27 (0.59 to 2.71; 0.543) | |
| ≥2 hrs | 0.60 (0.31 to 1.16; 0.130) | | 1.50 (0.68 to 3.31; 0.315) | |
| **Adjusted^a^ RR (95% CI; *p-value*) for time spent on social media within strata of parental education** | | | | |
| No social media use | Ref | Ref | Ref | Ref |
| 1 - <30 mins | 1.02 (0.65 to 1.61; 0.927) | 1.14 (0.70 to 1.85; 0.588) | 1.15 (0.80 to 1.66; 0.458) | 0.88 (0.49 to 1.57; 0.658) |
| 30 mins - <1 hr | 2.13 (1.40 to 3.23; <0.0001) | 1.59 (0.96 to 2.64; 0.071) | 1.45 (0.96 to 2.19; 0.077) | 1.01 (0.64 to 1.59; 0.979) |
| 1 - <2 hrs | 2.19 (1.47 to 3.27; <0.0001) | 1.48 (0.88 to 2.49; 0.138) | 0.92 (0.53 to 1.60; 0.772) | 1.13 (0.76 to 1.69; 0.534) |
| ≥2 hrs | 3.36 (2.34 to 4.84; <0.0001) | 2.05 (1.25 to 3.37; 0.005) | 1.09 (0.61 to 1.97; 0.762) | 1.50 (0.97 to 2.33; 0.067) |
| **Adjusted^a^ RR (95% CI; *p-value*) for time spent on social media and parental education** | | | | |
| No social media use | Ref | 1.41 (0.80 to 2.48; 0.235) | Ref | 1.41 (1.01 to 1.97; 0.041) |
| 1 - <30 mins | 1.02 (0.65 to 1.61; 0.927) | 1.61 (1.02 to 2.53; 0.040) | 1.15 (0.80 to 1.66; 0.458) | 1.24 (0.71 to 2.17; 0.449) |
| No social media use | Ref | 1.44 (0.83 to 2.50; 0.192) | Ref | 1.36 (0.98 to 1.89; 0.067) |
| 30 mins - <1 hr | 2.13 (1.40 to 3.23; <0.0001) | 2.29 (1.51 to 3.49; <0.0001) | 1.45 (0.96 to 2.19; 0.077) | 1.37 (0.84 to 2.24; 0.211) |
| No social media use | Ref | 1.56 (0.88 to 2.78; 0.125) | Ref | 1.40 (1.01 to 1.95; 0.041) |
| 1 - <2 hrs | 2.19 (1.47 to 3.27; <0.0001) | 2.32 (1.51 to 3.55; <0.0001) | 0.92 (0.53 to 1.60; 0.772) | 1.59 (1.05 to 2.42; 0.030) |
| No social media use | Ref | 1.61 (0.89 to 2.89; 0.113) | Ref | 1.35 (0.98 to 1.86; 0.068) |
| ≥2 hrs | 3.36 (2.33 to 4.84; <0.0001) | 3.30 (2.26 to 4.81; <0.0001) | 1.09 (0.61 to 1.97; 0.762) | 2.03 (1.33 to 3.11; 0.001) |
| **Adjusted^a^ measure of multiplicative effect modification and interaction (95% CI; *p-value*)** | | | |  |
| No social media use | Ref | | Ref | |
| 1 - <30 mins | 1.12 (0.60 to 2.07; 0.721) | | 0.76 (0.39 to 1.51; 0.438) | |
| 30 mins - <1 hr | 0.75 (0.40 to 1.39; 0.359) | | 0.69 (0.37 to 1.29; 0.249) | |
| 1 - <2 hrs | 0.68 (0.37 to 1.25; 0.210) | | 1.23 (0.61 to 2.48; 0.562) | |
| ≥2 hrs | 0.61 (0.33 to 1.12; 0.110) | | 1.37 (0.66 to 2.86; 0.393) | |

**Legend:** Questionnaire imputed sample: n = 8,954 (weighted sample: n = 6,976). Time-use-diary imputed sample: n = 2,520 (weighted sample: n = 5,727). ^a^ Adjusted for: ethnicity, sex, parental cigarette use, parental e-cigarette use, parenting style, previous cigarette use, previous e-cigarette use, anti-social behaviour, previous alcohol use, urbanicity, age, number of siblings in household, maternal age at participant birth, in-person activities, cognitive ability, mental health, and risk-taking. Values may not add up due to rounding. Abbreviations: CI = Confidence interval; Hr/s = Hour/s; Min/s = Minute/s; n = Number of participants; Ref = Reference category, and RR = Risk ratio.

Table-H7. Participant cigarette use according to time spent on social media, within strata of parental education and according to ‘combinations’ of time spent on social media and parental education within the questionnaire and time-use-diary complete case samples

|  | **Questionnaire complete case sample (n = 6,234)** | | **Time-use-diary complete case sample (n = 2,109)** | |
| --- | --- | --- | --- | --- |
|  | **High parental education** | **Low parental education** | **High parental education** | **Low parental education** |
| **Weighted prevalence % (observed n with outcome/without outcome)** | | | | |
| No social media use | 13.0 (36/273) | 18.8 (26/121) | 20.3 (126/575) | 31.6 (76/201) |
| 1 - <30 mins | 14.2 (77/467) | 21.0 (45/209) | 25.5 (65/243) | 11.4 (13/95) |
| 30 mins - <1 hr | 23.9 (136/490) | 27.0 (78/235) | 24.0 (54/176) | 21.7 (20/81) |
| 1 - <2 hrs | 26.5 (178/522) | 26.8 (108/279) | 29.0 (37/112) | 40.1 (29/53) |
| ≥2 hrs | 37.7 (586/1,095) | 39.4 (460/813) | 33.3 (31/57) | 49.9 (24/41) |
| **Unadjusted RR (95% CI; *p-value*) for time spent on social media within strata of parental education** | | | | |
| No social media use | Ref | Ref | Ref | Ref |
| 1 - <30 mins | 1.10 (0.69 to 1.74; 0.699) | 1.12 (0.66 to 1.89; 0.670) | 1.25 (0.96 to 1.64; 0.094) | 0.36 (0.17 to 0.75; 0.007) |
| 30 mins - <1 hr | 1.84 (1.17 to 2.90; 0.009) | 1.44 (0.92 to 2.26; 0.111) | 1.18 (0.86 to 1.63; 0.308) | 0.69 (0.38 to 1.23; 0.203) |
| 1 - <2 hrs | 2.04 (1.37 to 3.06; 0.001) | 1.43 (0.90 to 2.28; 0.133) | 1.43 (0.94 to 2.17; 0.097) | 1.27 (0.87 to 1.85; 0.222) |
| ≥2 hrs | 2.91 (1.95 to 4.34; <0.0001) | 2.10 (1.39 to 3.19; <0.0001) | 1.64 (1.09 to 2.47; 0.018) | 1.58 (1.03 to 2.42; 0.037) |
| **Unadjusted RR (95% CI; *p-value*) for time spent on social media and parental education** | | | | |
| No social media use | Ref | 1.44 (0.83 to 2.53; 0.197) | Ref | 1.56 (1.16 to 2.08; 0.003) |
| 1 - <30 mins | 1.10 (0.69 to 1.74; 0.699) | 1.62 (0.98 to 2.68; 0.061) | 1.25 (0.96 to 1.64; 0.094) | 0.56 (0.28 to 1.14; 0.109) |
| No social media use | Ref | 1.44 (0.83 to 2.53; 0.197) | Ref | 1.56 (1.16 to 2.08; 0.003) |
| 30 mins - <1 hr | 1.84 (1.17 to 2.90; 0.009) | 2.08 (1.33 to 3.25; 0.001) | 1.18 (0.86 to 1.63; 0.308) | 1.07 (0.61 to 1.88; 0.819) |
| No social media use | Ref | 1.44 (0.83 to 2.53; 0.197) | Ref | 1.56 (1.16 to 2.08; 0.003) |
| 1 - <2 hrs | 2.04 (1.37 to 3.06; 0.001) | 2.07 (1.32 to 3.23; 0.002) | 1.43 (0.94 to 2.17; 0.097) | 1.97 (1.30 to 2.99; 0.001) |
| No social media use | Ref | 1.44 (0.83 to 2.53; 0.197) | Ref | 1.56 (1.16 to 2.08; 0.003) |
| ≥2 hrs | 2.91 (1.95 to 4.34; <0.0001) | 3.04 (2.04 to 4.53; <0.0001) | 1.64 (1.09 to 2.47; 0.018) | 2.45 (1.63 to 3.68; <0.0001) |
| **Unadjusted measure of multiplicative effect modification and interaction (95% CI; *p-value*)** | | | | |
| No social media use | Ref | | Ref | |
| 1 - <30 mins | 1.02 (0.52 to 2.01; 0.948) | | 0.29 (0.13 to 0.62; 0.002) | |
| 30 mins - <1 hr | 0.78 (0.42 to 1.47; 0.446) | | 0.58 (0.30 to 1.13; 0.109) | |
| 1 - <2 hrs | 0.70 (0.37 to 1.31; 0.266) | | 0.89 (0.50 to 1.57; 0.683) | |
| ≥2 hrs | 0.72 (0.41 to 1.29; 0.274) | | 0.96 (0.53 to 1.75; 0.897) | |
| **Adjusted^a^ RR (95% CI; *p-value*) for time spent on social media within strata of parental education** | | | | |
| No social media use | Ref | Ref | Ref | Ref |
| 1 - <30 mins | 1.09 (0.72 to 1.63; 0.692) | 1.15 (0.70 to 1.88; 0.576) | 1.24 (0.95 to 1.63; 0.120) | 0.42 (0.20 to 0.87; 0.019) |
| 30 mins - <1 hr | 1.86 (1.24 to 2.78; 0.003) | 1.47 (0.94 to 2.31; 0.092) | 1.18 (0.85 to 1.64; 0.311) | 0.83 (0.47 to 1.45; 0.506) |
| 1 - <2 hrs | 1.97 (1.38 to 2.82; <0.0001) | 1.41 (0.90 to 2.21; 0.138) | 1.21 (0.79 to 1.84; 0.377) | 1.07 (0.71 to 1.61; 0.756) |
| ≥2 hrs | 2.77 (1.92 to 4.00; <0.0001) | 1.95 (1.29 to 2.95; 0.002) | 1.56 (0.99 to 2.47; 0.056) | 1.62 (1.09 to 2.40; 0.016) |
| **Adjusted^a^ RR (95% CI; *p-value*) for time spent on social media and parental education** | | | | |
| No social media use | Ref | 1.06 (0.63 to 1.78; 0.819) | Ref | 1.33 (1.00 to 1.78; 0.052) |
| 1 - <30 mins | 1.09 (0.72 to 1.63; 0.692) | 1.22 (0.80 to 1.86; 0.355) | 1.24 (0.95 to 1.63; 0.120) | 0.56 (0.27 to 1.14; 0.109) |
| No social media use | Ref | 1.18 (0.70 to 1.98; 0.537) | Ref | 1.23 (0.91 to 1.65; 0.177) |
| 30 mins - <1 hr | 1.86 (1.24 to 2.78; 0.003) | 1.74 (1.14 to 2.65; 0.011) | 1.18 (0.85 to 1.64; 0.311) | 1.01 (0.58 to 1.77; 0.959) |
| No social media use | Ref | 1.30 (0.78 to 2.17; 0.321) | Ref | 1.27 (0.94 to 1.73; 0.116) |
| 1 - <2 hrs | 1.97 (1.38 to 2.82; <0.0001) | 1.82 (1.21 to 2.76; 0.004) | 1.21 (0.79 to 1.84; 0.377) | 1.36 (0.83 to 2.22; 0.219) |
| No social media use | Ref | 1.33 (0.79 to 2.23; 0.282) | Ref | 1.24 (0.92 to 1.67; 0.155) |
| ≥2 hrs | 2.77 (1.92 to 4.00; <0.0001) | 2.59 (1.80 to 3.74; <0.0001) | 1.56 (0.99 to 2.47; 0.056) | 2.01 (1.41 to 2.87; <0.0001) |
| **Adjusted^a^ measure of multiplicative effect modification and interaction (95% CI; *p-value*)** | | | | |
| No social media use | Ref | | Ref | |
| 1 - <30 mins | 1.06 (0.57 to 1.97; 0.857) | | 0.34 (0.15 to 0.73; 0.006) | |
| 30 mins - <1 hr | 0.79 (0.45 to 1.41; 0.430) | | 0.70 (0.37 to 1.31; 0.260) | |
| 1 - <2 hrs | 0.71 (0.41 to 1.25; 0.238) | | 0.88 (0.51 to 1.51; 0.651) | |
| ≥2 hrs | 0.70 (0.41 to 1.20; 0.195) | | 1.04 (0.57 to 1.88; 0.905) | |

**Legend** Questionnaire complete case sample: n = 6,234 (weighted sample: n = 4,851). Time-use-diary complete case sample: n = 2,109 (weighted sample: n = 4,590). ^a^ Adjusted for: ethnicity, sex, parental cigarette use, parental e-cigarette use, parenting style, previous cigarette use, previous e-cigarette use, anti-social behaviour, previous alcohol use, urbanicity, age, number of siblings in household, maternal age at participant birth, in-person activities, cognitive ability, mental health, and risk-taking. Values may not add up due to rounding. Abbreviations: CI = Confidence interval; Hr/s = Hour/s; Min/s = Minute/s; n = Number of participants; Ref = Reference category, and RR = Risk ratio.

Table-H8. Participant e-cigarette use according to time spent on social media, within strata of parental education and according to ‘combinations’ of time spent on social media and parental education within the questionnaire and time-use-diary complete case samples

|  | **Questionnaire complete case sample (n = 6,234)** | | **Time-use-diary complete case sample (n = 2,109)** | |
| --- | --- | --- | --- | --- |
|  | **High parental education** | **Low parental education** | **High parental education** | **Low parental education** |
| **Weighted prevalence % (observed n with outcome/without outcome)** | | | | |
| No social media use | 9.8 (28/281) | 12.2 (21/126) | 15.1 (92/609) | 29.7 (72/205) |
| 1 - <30 mins | 9.0 (55/489) | 19.2 (46/208) | 16.1 (42/266) | 20.0 (14/94) |
| 30 mins - <1 hr | 18.6 (115/511) | 27.0 (71/242) | 19.3 (36/194) | 21.6 (21/80) |
| 1 - <2 hrs | 18.2 (138/562) | 21.7 (99/288) | 12.9 (26/123) | 32.5 (25/57) |
| ≥2 hrs | 27.2 (434/1,247) | 29.6 (382/891) | 15.8 (18/70) | 39.0 (21/44) |
| **Unadjusted RR (95% CI; *p-values*) for time spent on social media within strata of parental education** | | | | |
| No social media use | Ref | Ref | Ref | Ref |
| 1 - <30 mins | 0.92 (0.53 to 1.60; 0.765) | 1.57 (0.88 to 2.80; 0.128) | 1.07 (0.71 to 1.61; 0.756) | 0.67 (0.26 to 1.72; 0.407) |
| 30 mins - <1 hr | 1.91 (1.12 to 3.24; 0.017) | 2.21 (1.28 to 3.82; 0.005) | 1.28 (0.81 to 2.03; 0.286) | 0.73 (0.42 to 1.27; 0.258) |
| 1 - <2 hrs | 1.87 (1.11 to 3.14; 0.019) | 1.78 (1.01 to 3.12; 0.045) | 0.86 (0.48 to 1.53; 0.601) | 1.09 (0.70 to 1.70; 0.693) |
| ≥2 hrs | 2.78 (1.72 to 4.51; <0.0001) | 2.42 (1.45 to 4.05; 0.001) | 1.04 (0.60 to 1.83; 0.878) | 1.31 (0.75 to 2.30; 0.346) |
| **Unadjusted RR (95% CI; *p-value*) for time spent on social media and parental education** | | | | |
| No social media use | Ref | 1.25 (0.62 to 2.53; 0.529) | Ref | 1.97 (1.41 to 2.76; <0.0001) |
| 1 - <30 mins | 0.92 (0.53 to 1.60; 0.765) | 1.96 (1.10 to 3.49; 0.022) | 1.07 (0.71 to 1.61; 0.756) | 1.33 (0.53 to 3.35; 0.548) |
| No social media use | Ref | 1.25 (0.62 to 2.53; 0.529) | Ref | 1.97 (1.41 to 2.76; <0.0001) |
| 30 mins - <1 hr | 1.91 (1.12 to 3.24; 0.017) | 2.77 (1.61 to 4.77; <0.0001) | 1.28 (0.81 to 2.03; 0.286) | 1.43 (0.80 to 2.57; 0.227) |
| No social media use | Ref | 1.25 (0.62 to 2.53; 0.529) | Ref | 1.97 (1.41 to 2.76; <0.0001) |
| 1 - <2 hrs | 1.87 (1.11 to 3.14; 0.019) | 2.22 (1.29 to 3.84; 0.004) | 0.86 (0.48 to 1.53; 0.601) | 2.15 (1.41 to 3.30; <0.0001) |
| No social media use | Ref | 1.25 (0.62 to 2.52; 0.528) | Ref | 1.97 (1.41 to 2.76; <0.0001) |
| ≥2 hrs | 2.78 (1.72 to 4.51; <0.0001) | 3.03 (1.84 to 5.00; <0.0001) | 1.04 (0.60 to 1.83; 0.878) | 2.58 (1.49 to 4.47; 0.001) |
| **Unadjusted measure of multiplicative effect modification and interaction (95% CI; *p-value*)** | | | | |
| No social media use | Ref | | Ref | |
| 1 - <30 mins | 1.70 (0.78 to 3.70; 0.178) | | 0.63 (0.23 to 1.74; 0.371) | |
| 30 mins - <1 hr | 1.16 (0.55 to 2.46; 0.699) | | 0.57 (0.28 to 1.15; 0.116) | |
| 1 - <2 hrs | 0.95 (0.45 to 2.03; 0.898) | | 1.27 (0.57 to 2.87; 0.557) | |
| ≥2 hrs | 0.87 (0.43 to 1.77; 0.698) | | 1.25 (0.54 to 2.91; 0.598) | |
| **Adjusted^a^ RR (95% CI; *p-value*) for time spent on social media within strata of parental education** | | | | |
| No social media use | Ref | Ref | Ref | Ref |
| 1 - <30 mins | 0.93 (0.56 to 1.54; 0.781) | 1.57 (0.93 to 2.64; 0.093) | 1.11 (0.74 to 1.67; 0.599) | 0.69 (0.37 to 1.29; 0.244) |
| 30 mins - <1 hr | 2.14 (1.33 to 3.42; 0.002) | 2.51 (1.47 to 4.30; 0.001) | 1.44 (0.90 to 2.31; 0.132) | 0.85 (0.50 to 1.43; 0.536) |
| 1 - <2 hrs | 2.12 (1.31 to 3.45; 0.002) | 1.99 (1.15 to 3.44; 0.014) | 0.91 (0.52 to 1.60; 0.749) | 0.89 (0.58 to 1.36; 0.601) |
| ≥2 hrs | 3.21 (2.12 to 4.86; <0.0001) | 2.79 (1.71 to 4.55; <0.0001) | 1.04 (0.55 to 1.95; 0.904) | 1.25 (0.76 to 2.07; 0.384) |
| **Adjusted^a^ RR (95% CI; *p-value*) for time spent on social media and parental education** | | | | |
| No social media use | Ref | 1.06 (0.55 to 2.01; 0.869) | Ref | 1.65 (1.16 to 2.34; 0.005) |
| 1 - <30 mins | 0.93 (0.56 to 1.54; 0.781) | 1.65 (0.99 to 2.76; 0.055) | 1.11 (0.74 to 1.67; 0.599) | 1.13 (0.61 to 2.09; 0.688) |
| No social media use | Ref | 0.98 (0.52 to 1.85; 0.946) | Ref | 1.68 (1.20 to 2.34; 0.003) |
| 30 mins - <1 hr | 2.14 (1.33 to 3.42; 0.002) | 2.46 (1.50 to 4.04; <0.0001) | 1.44 (0.90 to 2.31; 0.132) | 1.42 (0.80 to 2.53; 0.233) |
| No social media use | Ref | 1.08 (0.57 to 2.02; 0.817) | Ref | 1.70 (1.21 to 2.38; 0.002) |
| 1 - <2 hrs | 2.12 (1.31 to 3.45; 0.002) | 2.14 (1.28 to 3.58; 0.004) | 0.91 (0.52 to 1.60; 0.749) | 1.52 (0.92 to 2.48; 0.099) |
| No social media use | Ref | 1.13 (0.60 to 2.10; 0.709) | Ref | 1.58 (1.13 to 2.21; 0.007) |
| ≥2 hrs | 3.21 (2.12 to 4.86; <0.0001) | 3.14 (2.03 to 4.87; <0.0001) | 1.04 (0.55 to 1.95; 0.904) | 1.98 (1.21 to 3.25; 0.007) |
| **Adjusted^a^ measure of multiplicative effect modification and interaction (95% CI; *p-values*)** | | | |  |
| No social media use | Ref | | Ref | |
| 1 - <30 mins | 1.68 (0.82 to 3.43; 0.153) | | 0.62 (0.29 to 1.30; 0.205) | |
| 30 mins - <1 hr | 1.18 (0.59 to 2.34; 0.640) | | 0.59 (0.31 to 1.14; 0.113) | |
| 1 - <2 hrs | 0.94 (0.47 to 1.87; 0.854) | | 0.98 (0.49 to 1.96; 0.950) | |
| ≥2 hrs | 0.87 (0.46 to 1.65; 0.669) | | 1.20 (0.53 to 2.72; 0.655) | |

**Legend:** Questionnaire complete case sample: n = 6,234 (weighted sample: n = 4,851). Time-use-diary complete case sample: n = 2,109 (weighted sample: n = 4,590). ^a^ Adjusted for: ethnicity, sex, parental cigarette use, parental e-cigarette use, parenting style, previous cigarette use, previous e-cigarette use, anti-social behaviour, previous alcohol use, urbanicity, age, number of siblings in household, maternal age at participant birth, in-person activities, cognitive ability, mental health, and risk-taking. Values may not add up due to rounding. Abbreviations: CI = Confidence interval; Hr/s = Hour/s; Min/s = Minute/s; n = Number of participants; Ref = Reference category, and RR = Risk ratio.

# REFERENCES

1. Atkin A, Dainty J, Dumuid D, et al. Adolescent time use and mental health: A cross-sectional, compositional analysis in the Millennium Cohort Study. *BMJ Open*. 2021;11(10):e047189. doi:10.1136/bmjopen-2020-047189

2. Twenge JM, Spitzberg BH, Campbell WK. Less in-person social interaction with peers among U.S. adolescents in the 21st century and links to loneliness. *J Soc Pers Relat*. 2019;36(6):1892-1913. doi:10.1177/0265407519836170

3. Fitzsimons E, Haselden L, Smith K, et al. Millennium Cohort Study: Age 17 sweep (MCS7) (second edition); 2020. https://cls.ucl.ac.uk/wp-content/uploads/2020/09/MCS7-user-guide-Age-17-ed1.pdf

4. Johnson DR. *Using weights in the analysis of survey data*; 2008. https://pages.nyu.edu/jackson/design.of.social.research/Readings/Johnson - Introduction to survey weights %28PRI version%29.pdf

5. Mostafa T, Ploubidis G. *Millennium Cohort Study: Sixth Survey 2015-2016 Technical Report on Response (Age 14)*; 2017. https://doc.ukdataservice.ac.uk/doc/8156/mrdoc/pdf/mcs6_report_on_response.pdf

6. Knol MJ, VanderWeele TJ, Groenwold RHH, Klungel OH, Rovers MM, Grobbee DE. Estimating measures of interaction on an additive scale for preventive exposures. *Eur J Epidemiol*. 2011;26(6):433-438. doi:10.1007/s10654-011-9554-9

7. Knol MJ, VanderWeele TJ. Recommendations for presenting analyses of effect modification and interaction. *Int J Epidemiol*. 2012;41(2):514-520. doi:10.1093/ije/dyr218

8. Vanderweele TJ. On the distinction between interaction and effect modification. *Epidemiology*. 2009;20(6):863-871. doi:10.1097/EDE.0b013e3181ba333c

9. Social Science Computing Operative. *Multiple imputation in Stata*.; 2013. https://www.ssc.wisc.edu/sscc/pubs/stata_mi_intro.htm
